# Total Synthesis of Synechoxanthin through Iterative Cross-Coupling**

**DOI:** 10.1002/anie.201102688

**Published:** 2011-06-16

**Authors:** Seiko Fujii, Stephanie Y Chang, Martin D Burke

**Affiliations:** Howard Hughes Medical Institute, Department of Chemistry, University of Illinois at Urbana-Champaign600 S. Mathews Ave, Urbana, IL 61801 (USA), Fax: (+1) 217-244-8024 E-mail: burke@scs.uiuc.edu Homepage: http://www.scs.illinois.edu/burke

**Keywords:** antioxidants, boronates, iterative cross-coupling, synechoxanthin, total synthesis

Deficiencies of human proteins that protect cells from lipid peroxidation have been linked to many prevalent diseases, including atherosclerosis, neurodegenerative disorders, and cancer.^1^ Remarkably, some species of bacteria have the ability to thrive in environments of extreme oxidative stress, which has been attributed to the presence of specialized carotenoids in their membranes.^2^ These natural products might therefore serve as valuable prototypes for understanding and optimizing the capacity for small molecules to serve as antilipoperoxidants in human cells. In this vein, a structurally unique aromatic dicarboxylate carotenoid, synechoxanthin (**1**), was isolated in 2008 from the exceptionally reactive oxygen species (ROS)-resistant cyanobacterium *Synechococcus* sp. strain PCC 7002.^3^ Knocking out **1** through genetic manipulation of its biosynthetic machinery substantially diminishes this ROS resistance.^4^ With the ultimate goal of understanding and optimizing the promising antioxidant activity of this natural product, we herein report its first total synthesis. This synthesis was achieved using only one reaction iteratively to assemble three simple and readily accessible building blocks in a completely stereocontrolled fashion. This route was enabled by a novel iterative cross-coupling (ICC) strategy, in which the polarity of the bifunctional building blocks is reversed to match the preferred polarity for cross-coupling. Moreover, a final one-pot boronate hydrolysis/two-directional double cross-coupling sequence enabled rapid assembly of the *C*_2_-symmetric carotenoid core in a highly convergent fashion. The efficient, completely stereocontrolled, and inherently flexible nature of this building block-based pathway has opened the door to systematic studies of the antioxidant functions of **1** and its derivatives.

The highly complex nonaene framework found in **1** and many other *C*_2_-symmetric carotenoids represents a substantial structural and stereochemical challenge. The most commonly employed strategy to access this motif involves a double Wittig olefination between a C_10_-trienedialdehyde and two C_15_-polyenylphosphonate salts, which typically leads to mixtures of olefin stereoisomers.^5^ This approach can be effective when combined with a highly optimized post-olefination isomerization protocol specifically tailored for each carotenoid target.^5^ However, if the goal is to gain unfettered access to structural derivatives, then this approach is quite limited.

The use of only stereospecific cross-coupling reactions to assemble stereochemically defined polyene building blocks represents an attractive alternative.^6^ Ideally, the building blocks and intermediates in such a pathway would be non-toxic, stable, and readily accessible. With these goals in mind, we recently introduced a simple, efficient, and flexible strategy for small-molecule synthesis that involves the ICC of haloboronic acids (Figure 1). In our original approach, nucleophilic sp^2^(B)-hybridized boronic acids are coupled to the halide termini of bifunctional building blocks having their boronic acid termini masked as the corresponding sp^3^(B)-hybridized *N*-methyliminodiacetic acid (MIDA) boronates (Figure 1 A).^7^, ^8^

**Figure 1:**
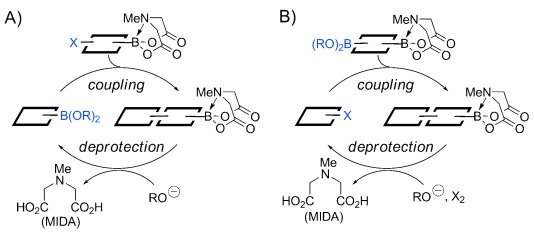
A) ICC with haloboronic acids in which the MIDA boronate serves as a masked boronic acid. B) A novel ICC strategy in which the polarity of the bifunctional building blocks is reversed and the MIDA boronate serves as a masked halide.

In the process of exploring the application of this strategy to a synthesis of **1**, we recognized an opportunity to achieve optimal intermediates for cross-coupling by alternatively starting with an electrophilic organohalide and reversing the polarity of the bifunctional building blocks employed in the ICC sequence (Figure 1 B).^9^ Specifically, **1** contains electron-withdrawing carboxylic acids at its termini. Electron-deficient boranes are, in general, poor cross-coupling partners due to an increased propensity for protodeboronation and homocoupling.^10^ In contrast, electron-deficient halides tend to be excellent intermediates, often cross-coupling under milder conditions and/or in higher yields than their electron-neutral and -rich counterparts.^11^, ^12^ Guided by this logic, we retrosynthesized **1** into three simple building blocks, **2**,^13^ **3**, and **4**^14^ using only Suzuki–Miyaura (SM) transforms that involve activated, electron-deficient halide intermediates (Scheme 1).

**Scheme 1:**
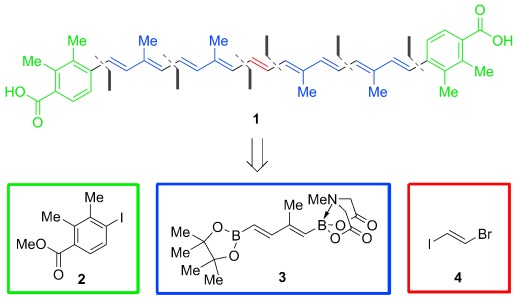
Retrosynthesis of **1** by ICC.

This plan required a new type of bifunctional building block containing a nucleophilic boron terminus and a protected electrophilic halide. Mild and general methods for halide masking are scarce,7f, 15 but it is known that nucleophilic vinylboronic acids can be transformed into electrophilic iodides with retention of stereochemistry.^16^ Thus, we pursued the development of bifunctional building block **3** in which the MIDA boronate motif serves a new role as a masked electrophile.

The capacity to carry MIDA boronates through multiple chemical transformations7c enabled facile preparation of **3** (Scheme 2). Specifically, transesterification of **5**7g afforded pinacol ester **6**, and trisubstituted olefin **7**7e underwent stereoretentive iododestannylation to afford vinyl iodide **8**. Subsequent Stille coupling between **6** and **8** afforded **3** as a stable, crystalline solid that can be stored for more than six months without any noticeable decomposition. Distinct hybridization states (sp^2^ and sp^3^) for the two boron atoms in **3** were confirmed unambiguously via single crystal X-ray analysis (Scheme 2).

**Scheme 2:**
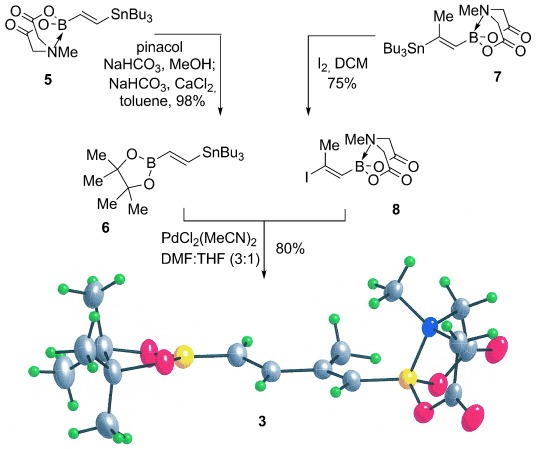
Synthesis of bisborylated diene **3**.

With these building blocks in hand, an efficient, polarity-reversed ICC-based synthesis of key intermediate **11** was achieved (Scheme 3). Specifically, SM coupling of activated, electron-deficient aryl iodide **2** with the sp^2^(B)-hybridized terminus of bisborylated building block **3**^17^ afforded **9** in very good yield as a single stereoisomer. MIDA boronate **9** was then halodeborylated in a single-pot operation using NaOMe and I_2_ to afford **10** in quantitative yield and with complete retention of stereochemistry. This transformation unmasked a new electron-deficient halide for a second iteration of SM coupling. Specifically, activated dienyl iodide **10** was smoothly coupled with another equivalent of **3** to afford stereochemically pure tetraenyl MIDA boronate **11**. Both of the complex polyenyl MIDA boronates **9** and **11** proved to be stable crystalline solids that are compatible with standard silica gel chromatography.

**Scheme 3:**
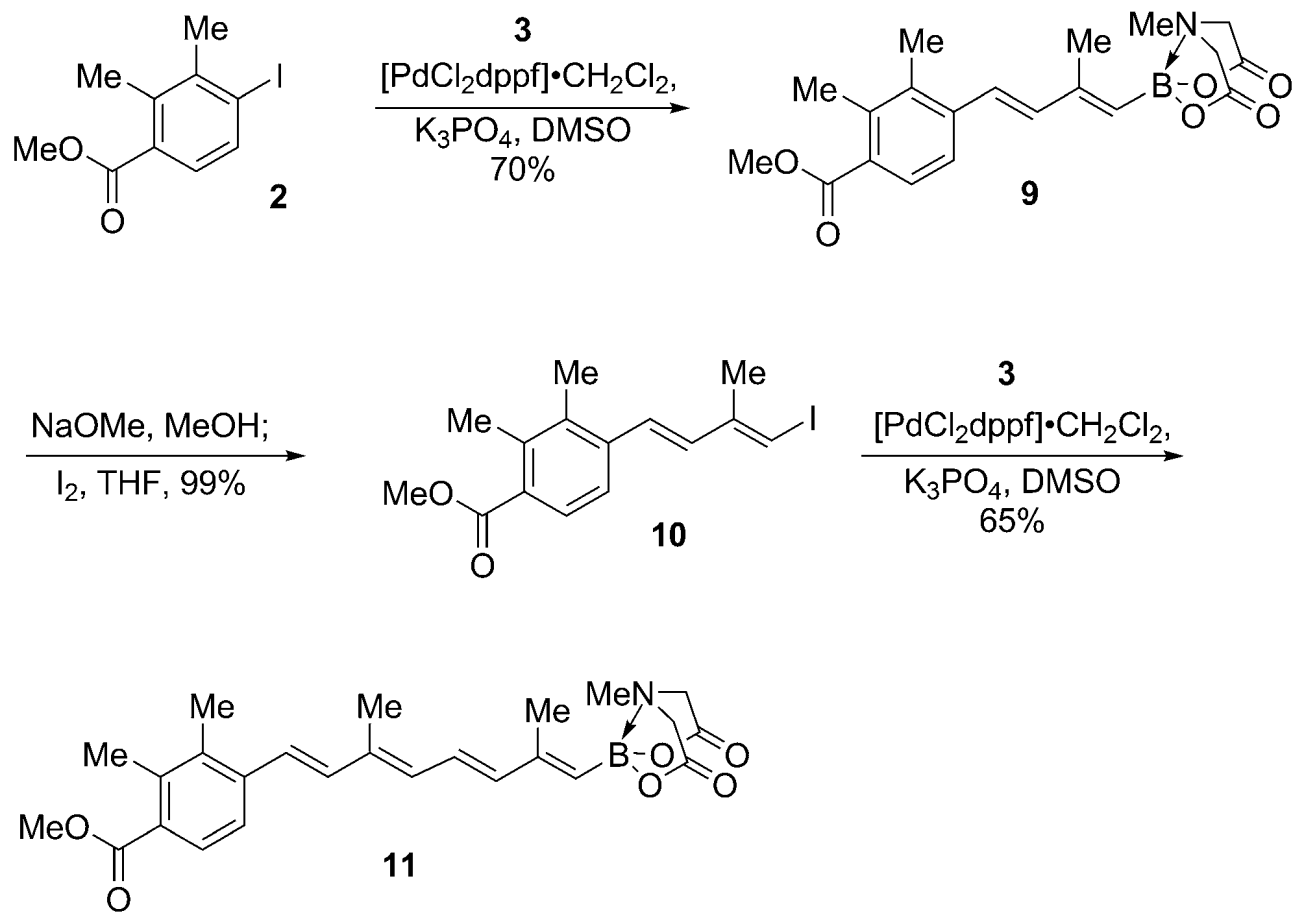
Synthesis of key intermediate **11** by ICC. dppf = 1,1′-bis(diphenylphosphino)ferrocene.

Finally, harnessing the capacity of the versatile MIDA boronate motif to also represent a masked boronic acid which can be released and coupled in situ and thereby obviate the isolation of unstable intermediates,7d a highly convergent and stereospecific assembly of the complete polyene framework of **1** was achieved (Scheme 4). Specifically, an in situ MIDA boronate hydrolysis/two-directional double cross-coupling sequence between two equivalents of **11** and electronically activated *trans*-1-iodo-2-bromoethylene **4** yielded synechoxanthin bismethylester **12** in an overall very efficient one-pot operation. To the best of our knowledge, **12** represents the longest polyene prepared to date through SM coupling. Concomitant hydrolysis of the terminal methyl esters completed the first total synthesis of **1**.

**Scheme 4:**
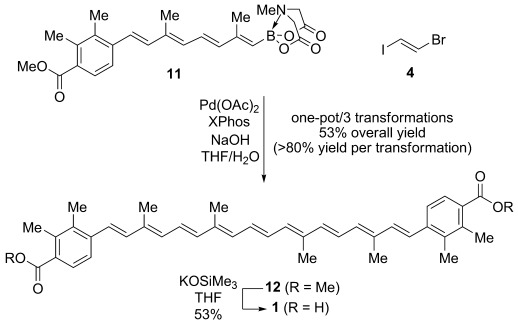
Highly convergent assembly of synechoxanthin (**1**).

The strategic advances achieved with this pathway have substantially expanded the power and flexibility of ICC as an increasingly general platform for small-molecule synthesis. Moreover, because this building block-based synthesis of **1** is efficient, convergent, completely stereocontrolled, modular, and involves stable intermediates, it stands to enable systematic dissection of the structure/function relationships that underlie the very promising activities of this natural antioxidant.

## Supplementary material

Detailed facts of importance to specialist readers are published as ”Supporting Information”. Such documents are peer-reviewed, but not copy-edited or typeset. They are made available as submitted by the authors.
