# Supplementary material for: Total Synthesis of Synechoxanthin through Iterative Cross-Coupling
Source: Angew Chem Int Ed Engl. 2011 Jun 16;50(34):7862–4. doi: 10.1002/anie.201102688 (PMC3433251; doi:10.1002/anie.201102688)
Supplement: Supplementary file 1 [file anie0050-7862-SD1.pdf]

Angewandte Chemie
Eine Zeitschrift der Gesellschaft Deutscher Chemiker

Supporting Information
© Wiley-VCH 2011
69451 Weinheim, Germany

Total Synthesis of Synechoxanthin through Iterative Cross-Coupling**

*Seiko Fujii, Stephanie Y. Chang, and Martin D. Burke**

anie_201102688_sm_miscellaneous_information.pdf

SUPPORTING INFORMATION

Part A
I. General Methods S1-S2
II. Synthesis of **2** S3-S8
III. Synthesis of **3** S9-S12
IV. Total synthesis of **1** S13-S22
Part B
NMR Spectra (PDF file) S23-S52

I. General methods

Materials. Commercial reagents were purchased from Sigma-Aldrich, TCI America, Alfa Aesar, Strem Chemicals Inc. or Fisher Scientific and used without further purification unless otherwise noted. Solvents were purified via passage through packed columns as described by Pangborn and coworkers[1] (THF, Et_2O, CH_3CN, CH_2Cl_2: dry neutral alumina; hexane, benzene, and toluene: dry neutral alumina and Q5 reactant; DMSO, DMF: activated molecular sieves). All water was deionized prior to use. The following compounds were prepared by their literature methods: **SI-1**[2] and **4**.[3] The following compounds were prepared as previously described: MIDA boronate **5**,[4] and vinyl stannane **7**.[5]

General Experimental Procedures. All reactions were performed in flame- or oven (125 °C)-dried glassware equipped with a stir bar under an atmosphere of dry argon unless otherwise stated. Organic solutions were concentrated via rotary evaporation under reduced pressure with a bath temperature of 30-40 °C. Reactions were monitored by analytical thin layer chromatography (TLC) on Merck silica gel 60 F254 plates (0.25 mm) using the indicated solvent system. Compounds were visualized by exposure to UV light (254 nm) or potassium permanganate ($KMnO_4$) followed by brief heating with a Varitemp heat gun. MIDA boronates are compatible with standard silica gel chromatography, including standard loading techniques. Column chromatography was performed using standard methods[6] or on a Teledyne- Isco CombiFlash Rf purification system using Merck silica gel grade 9385 60Å (230-400 mesh). For loading, compounds were adsorbed onto non acid-washed Celite *in vacuo* from an acetone solution. Specifically, for a 1 g mixture of crude material the sample is dissolved in reagent grade acetone (25 to 50 mL) and to the flask is added Celite 545 Filter Aid (5 to 15 g). The mixture is then concentrated *in vacuo* to afford a powder, which is then loaded on top of a silica gel column. Flash column chromatography was performed as described by Still and coworkers[1] using Merck silica gel 60 (230-400 mesh). RP-HPLC purification was performed on Agilent 1100 series HPLC system equipped with a Sunfire™ C_{18} 5 micron 10 x 250 mm column (Waters Corp. Milford, MA) with UV detection at 478 nm and the indicated eluent and flow rate.

---

[1] A. B. Pangborn, M. A. Giardello, R. H. Grubbs, R. K. Rosen, F. J. Timmers, *Organometallics* **1996**, *15*, 1518.
[2] F. Kaiser, L. Schwink, J. Velder, H.-G. Schmalz, *Tetrahedron* **2003**, *59*, 3201-3217.
[3] E. Negishi, A. Alimardanov, C. Xu, *Org. Lett.* **2000**, *2*, 65-67.
[4] J. R. Struble, S. J. Lee, M. D. Burke, *Tetrahedron* **2010**, *66*, 4710-4718.
[5] E. M. Woerly, A. H. Cherney, E. K. Davis, M. D. Burke, *J. Am. Chem. Soc.* **2010**, *132*, 6941-6943.
[6] W. C. Still, M. Kahn, A. Mitra, *J. Org. Chem.* **1978**, *43*, 2923.

Structural Analysis. ^{1}H NMR spectra were recorded at 23 °C using one of the following instruments: Varian Unity Inova 500 (500 MHz), Varian VXR 500 (500 MHz), and Varian Unity Inova 500NB (500 MHz). Chemical shifts are reported in parts per million (ppm) downfield from tetramethylsilane and referenced to residual protium in the NMR solvent ($CDCl_3$, δ = 7.26; acetone-d_6, δ = 2.04; CD_2Cl_2, δ = 5.32, center line). When solvent mixtures were used, spectra were referenced to an internal standard of tetramethylsilane (δ = 0.00). Spectral data are presented as follows: chemical shift, multiplicity (s = singlet, d = doublet, t = triplet, q = quartet, quint = quintet, sext = sextet, m = multiplet, b = broad), coupling constant (*J*), and integration. ^{13}C NMR spectra were recorded at 23 °C using one of the following instruments: Varian Unity Inova 500 (500 MHz), Varian VXR 500 (500 MHz), or Varian Unity Inova 600 (600 MHz). Chemical shifts are reported in parts per million (ppm) downfield from tetramethylsilane and referenced to carbon resonances in the NMR solvent ($CDCl_3$, δ = 77.0; acetone-d_6, δ = 29.8; CD_2Cl_2, δ = 53.8; DMSO-d_6, δ = 49.0; CD_3CN, δ = 118.2, center line) or to added tetramethylsilane (δ = 0.00). Many of the carbon bearing boron substituents were not observed (quadrupole relaxation). ^{11}B NMR were recorded at 23 °C on a Varian Unity Inova 400 instrument and referenced to an external standard of BF_3•Et_2O. High-resolution mass spectra (HRMS) were performed by Furong Sun, Elizabeth Eves, and Dr. Haijun Yao at the University of Illinois School of Chemical Sciences Mass Spectrometry Laboratory. Data are reported in the form of m/z. Infrared spectra were collected from a thin film on NaCl plates on a Mattson Galaxy Series FT-IR 5000 spectrometer. Absorption maxima ($\#_{max}$) are reported in wavenumbers (cm^{-1}). X-ray crystallographic analysis was carried out by Dr. Danielle Gray at the University of Illinois George L. Clark X-Ray facility.

II. Synthesis of 2

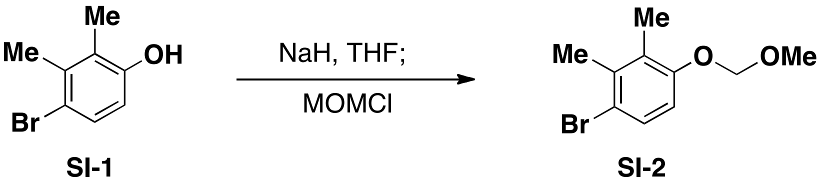

Aryl bromide, SI-2. A 500 mL round bottom flask was charged with NaH (60 wt% in mineral oil, 2.911 g, 72.8 mmol, 1.22 equiv.) and THF (200 mL, 0.24 M) and the reaction mixture was cooled to 0 °C for 30 min. A 100 mL pear-shaped flask was charged with **SI-1**[2] (12 g, 59.7 mmol, 1.0 equiv.) and THF (50 mL x 2) to afford a clear, pale yellow solution. The **SI-1** solution was transferred dropwise via cannula into the reaction flask over 20 min at 0 °C while vigorous H_2 evolution was observed. The reaction mixture was warmed to 23 °C over 30 min. The reaction mixture was cooled again to 0 °C and MOMCl (6.0 mL, 79.4 mmol, 1.33 equiv.) was added dropwise to the reaction mixture over 10 min. The reaction mixture was warmed to 23 °C with stirring over 2 h under positive Ar pressure. After 2 h, saturated aqueous NH_4Cl (500 mL) was added to the reaction mixture. The resulting biphasic mixture was transferred to a 2-L separatory funnel, rinsing with diethyl ether (500 mL) for quantitative transfer. The layers were separated and the aqueous layer was extracted with diethyl ether (500 mL). The combined organic layers were washed with 1M aqueous NaOH (2 x 200 mL), H_2O (200 mL), and brine (200 mL), dried over $MgSO_4$, filtered through a plug of Celite and silica gel, and concentrated *in vacuo* to afford **SI-2** as a clear yellow oil (12.37 g, 85%).

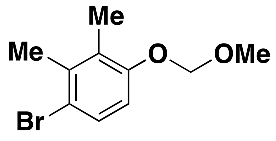

SI-2

TLC (hexanes:EtOAc 3:1)

R_f = 0.64, stained by $KMnO_4$

^{1}H-NMR (500 MHz, $CDCl_3$)

δ 7.32 (d, J = 9.0 Hz, 1H), 6.82 (d, J = 9.0 Hz, 1H), 5.16 (s, 2H), 3.47 (s, 3H), 2.37 (s, 3H), 2.24 (s, 3H).

^{13}C-NMR (125 MHz, $CDCl_3$)

δ 154.3, 137.2, 129.8, 128.0, 117.7, 113.3, 94.8, 56.0, 20.0, 13.1

HRMS (EI+)

Calculated for $C_{10}H_{13}O_2Br$: 244.00989

Found: 244.00851

IR (thin film, cm^{-1})

2992, 2927, 2854, 2825, 2073, 1641, 1573, 1461, 1403, 1382, 1307, 1253, 1205, 1157.08, 1099, 1066, 1002, 923, 892, 802.

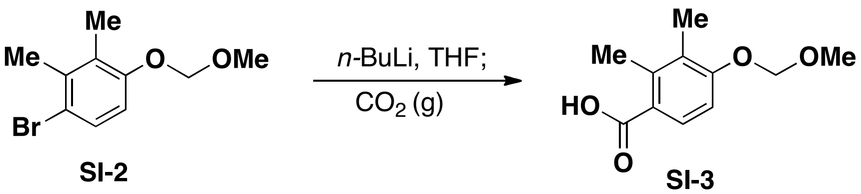

Benzoic acid, SI-3. A 100 mL round bottom flask was charged with **SI-2** (4.27 g, 17.4 mmol, 1.0 equiv). THF (58 mL, 0.3M) was added to afford a clear, pale yellow solution and the reaction mixture was cooled to -78 °C over 10 min. *n*-BuLi (2.5M, 7.45 mL, 18.63 mmol) was added dropwise to the reaction mixture, which was stirred for 10 min at -78 °C. CO_2 (g) was bubbled through the reaction mixture at -78 °C for 10 min while the reaction mixture turned from pale yellow to bright red, then back to pale yellow over 10 min. The reaction mixture was warmed to 23 °C over 30 min. The crude mixture was quenched by the addition of 1M HCl (50 mL) and transferred to a separatory funnel, diluting with EtOAc (50 mL). The layers were separated and the aqueous layer was extracted with EtOAc (50 mL). The combined organic layers were dried over $MgSO_4$, filtered, and concentrated *in vacuo*. The crude solid was washed with hexanes, filtered, and concentrated *in vacuo* to afford **SI-3** as a pale pink solid (2.37 g, 65%).

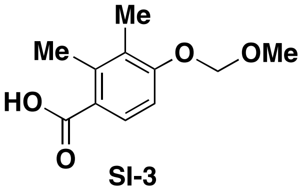

SI-3

TLC (hexanes:EtOAc 3:1)

R_f = 0.19, stained by $KMnO_4$

^{1}H-NMR (500 MHz, $CDCl_3$)

δ 7.87 (d, *J* = 9.0 Hz, 1H), 6.97 (d, *J* = 8.5 Hz, 1H), 5.26 (s, 2H), 3.49 (s, 3H), 2.58 (s, 3H), 2.23 (s, 3H).

^{13}C-NMR (125 MHz, $CDCl_3$)

δ 173.4, 158.4, 141.4, 130.5, 127.0, 122.5, 110.5, 94.2, 56.2, 17.2, 12.0

HRMS (ESI+)

Calculated for $C_{11}H_{14}O_4Na$:	233.0790
Found:	233.0793

IR (thin film, cm^{-1})

3438, 3330, 3060, 2962, 2923, 2865, 2854, 2362, 1681, 1579, 1479, 1432, 1384, 1257.36, 1189, 1174, 1151, 1097, 1066, 1027, 914.

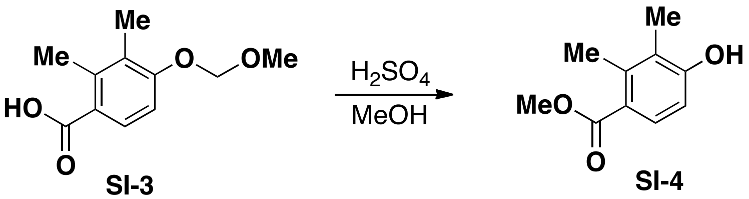

SI-3 → SI-4

Methyl Benzoate, SI-4. A 100 mL round bottom flask was charged with benzoic acid **SI-3** (2.49 g, 11.86 mmol, 1.0 equiv.), H_2SO_4 (1.0 mL, 1.0 equiv.) and MeOH (40 mL, 0.3 M). The flask was fitted with a reflux condenser and a gas inlet needle and flushed with N_2. The reaction mixture was refluxed in an oil bath for 13 h. The reaction mixture turned clear brown. After 13 h, the reaction mixture was cooled to 23 ^oC and concentrated *in vacuo*. The crude mixture was dissolved in diethyl ether (50 mL) and neutralized with K_2CO_3, dried over $MgSO_4$, filtered through a plug of silica gel, and concentrated *in vacuo* to afford **SI-4** as an off-white solid (1.91g, 89%).

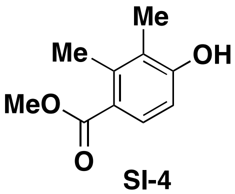

SI-4

TLC (hexanes:EtOAc 5:1)

R_f = 0.23, stained by $KMnO_4$

^{1}H-NMR (500 MHz, $CDCl_3$)

δ 7.64 (d, *J* = 8.5 Hz, 1H), 6.64 (d, *J* = 8.5 Hz, 1H), 5.02 (s, 1H), 3.85 (s, 3H), 2.52 (s, 3H), 2.20 (s, 3H).

^{13}C-NMR (125 MHz, $CDCl_3$)

δ 168.6, 156.4, 140.9, 129.4, 123.7, 123.2, 112, 51.7, 17.1, 11.7

HRMS (ESI+)

Calculated for $C_{10}H_{13}O_3$:	180.0865
Found:	180.0872

IR (thin film, cm^{-1})

3467, 3419, 3037, 2996, 2948, 2117, 1650, 1581, 1490, 1446, 1432, 1373, 1340, 1278, 1191, 1174, 1147, 1072.

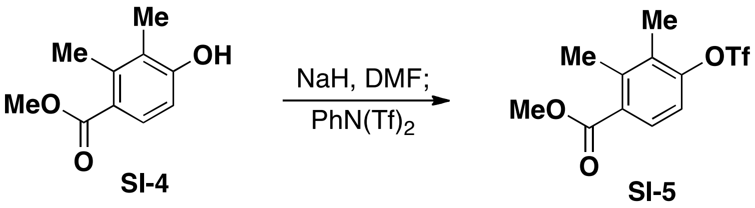

Triflate, SI-5. A 50 mL round bottom flask was charged with NaH (60 wt% in mineral oil, 231 mg, 5.78 mmol, 1.0 equiv.) and DMF (7.0 mL) and cooled to 0 °C. A 50-mL pear flask was charged with **SI-4** (1.042 g, 5.78 mmol, 1.0 equiv) and DMF (12 mL) to afford a clear yellow solution. The **SI-4** solution was added dropwise to the reaction flask at 0 °C. The reaction mixture turned greenish grey. The reaction mixture was warmed to 23 °C and stirred for 30 min. After 30 min, the reaction flask was once again cooled to 0 °C and $NPhTf_2$ (2.065 g, 5.78 mmol, 1.0 equiv.) was added in small portions. The reaction mixture was slowly warmed to 23 °C overnight with stirring. After 12 h, the yellow/brown reaction mixture was diluted with saturated aqueous NH_4Cl (60 mL) while stirring. The mixture was transferred to a separatory funnel, rinsing with diethyl ether (60 mL). The aqueous layer was extracted with ether (60 mL x 2). The combined organic layers were washed with water to remove residual DMF. The organic layer was dried over $MgSO_4$, filtered, and concentrated *in vacuo* to afford a yellow oil. The crude material was adsorbed onto Celite from an acetone solution and purified by MPLC (Hexanes → Hexanes: EtOAc 4:1) to afford **SI-5** as a clear, yellow oil (1.65 g, 89% yield).

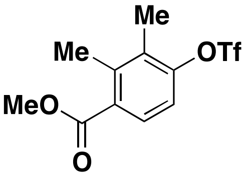

SI-5

TLC (hexanes:EtOAc 5:1)

R_f = 0.56, stained by $KMnO_4$

^{1}H-NMR (500 MHz, $CDCl_3$)

δ 7.69 (d, *J* =8.5 Hz, 1H), 7.16 (d, *J* = 8.5 Hz, 1H), 3.91 (s, 3H), 2.52 (s, 3H), 2.33 (s, 3H).

^{13}C-NMR (125 MHz, $CDCl_3$)

δ 167.7, 150.0, 141.3, 131.1, 131.0, 128.9, 119.8, 118.4, 52.3, 17.3, 13.4

HRMS (ESI+)

Calculated for $C_{11}H_{12}O_5SF_3$: 313.0358

Found: 313.0361

IR (thin film, cm^{-1})

3033, 3014, 2956, 2881, 2352, 2090, 1725, 1643, 1585, 1477, 1425, 1297, 1253, 1224.58, 1160, 1141, 1060, 1031, 931.

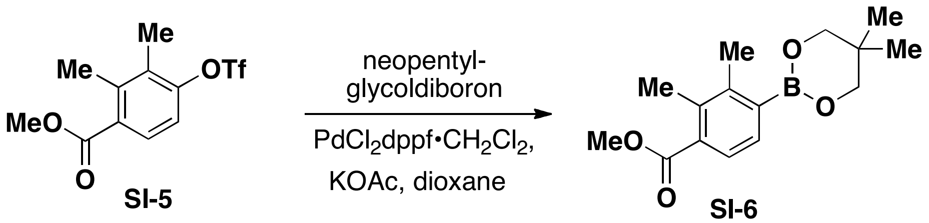

Neopentyl glycol boronic ester, SI-6. In a glove box, to a 40 mL vial charged with **SI-5** (949 mg, 3.04 mmol, 1.0 equiv.) was added neopentylglycolatodiboron (1.03 g, 4.56 mmol. 1.5 equiv.), $PdCl_2dppf•CH_2Cl_2$ (124 mg, 0.15 mmol, 5 mol%), and KOAc (895 mg, 9.11 mmol, 3.0 equiv.), followed by dioxane (15 mL, 0.2 M). The vial was sealed with a PTFE-lined cap under Ar and removed from the glovebox. The vial was placed in an 80 ^oC aluminum heat block and maintained at that temperature with stirring for 18 h. After 18 h, the reaction mixture was cooled to 23 ^oC and diluted with EtOAc and filtered through a plug of silica gel. The crude mixture was adsorbed onto Celite from an acetone solution and purified by MPLC (Hexanes→ 1:4 EtOAc:Hexanes) and the collected fractions were concentrated *in vacuo* to afford neopentyl glycol boronic ester **SI-6** as a white solid (555 mg, 66% yield).

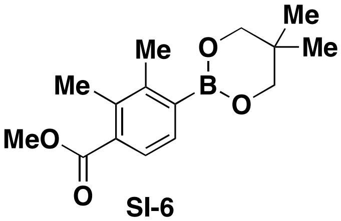

SI-6

TLC (hexanes:EtOAc 4:1)
R_f = 0.68, stained by $KMnO_4$

^{1}H-NMR (500 MHz, $CDCl_3$)
δ 7.52 (s, 2H), 3.88 (s, 3H), 3.79 (s, 4H), 2.47 (s, 3H), 2.43 (s, 3H), 1.05 (s, 6H).

^{13}C-NMR (125 MHz, $CDCl_3$)
δ 169.6, 142.9, 136.7, 132.4, 131.3, 126.0, 72.4, 51.9, 31.6, 21.9, 19.1, 16.8

^{11}B-NMR (128 MHz, acetone-d_6)
δ 27.8

HRMS (EI+)
Calculated for $C_{15}H_{21}O_4B$: 276.15330
Found: 276.15268

IR (thin film, cm^{-1})
3531, 3419, 2956, 2893, 1724, 1556, 1479, 1419, 1377, 1317, 1284, 1155, 1078, 1074.

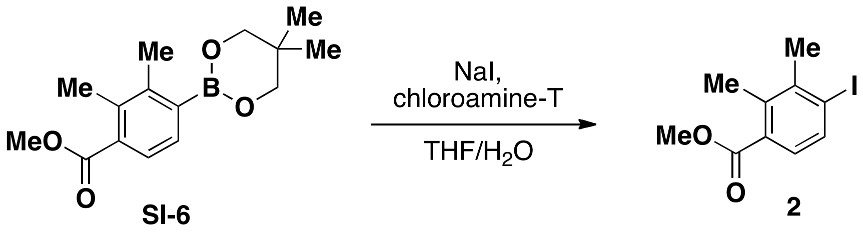

Aryl iodide, 2. A 20 mL vial was charged with neopentylglycol boronic ester **SI-6** (555 mg, 2.01 mmol, 1.0 equiv.) and THF:H_2O (1:1, 0.8 mL, 2.5 M) to afford a pale yellow solution and the vial was cooled to 0 °C. A solution of NaI (1.63 g, 2.51 mmol, 1.25 equiv.) in H_2O (2.5 mL) was added dropwise to the reaction vial followed by chloroamine-T (915 mg, 4.02 mmol, 2.0 equiv.) in THF:H_2O (1:1, 8.04 mL, 0.5 M). The reaction mixture was stirred under N_2 at 0 °C and warmed to 23 °C over 13 h. The reaction mixture turned from dark purple to orange over the course of the reaction. The mixture was quenched with saturated $Na_2S_2O_3$ solution (5 mL) and transferred to a separatory funnel, rinsing with diethyl ether (40 mL) and H_2O (40 mL). The layers were separated and the aqueous layer was extracted with diethyl ether (2 x 40 mL). The combined organic layers were dried over $MgSO_4$, filtered, and concentrated *in vacuo*. The resulting solid was adsorbed onto Celite from an acetone solution and purified by SiO_2 chromatography (100:1 petroleum ether:EtOAc) to afford **2** as a pale yellow solid (445 mg, 76%).

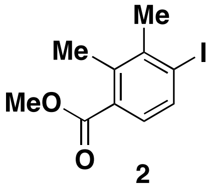

2

TLC (hexanes:EtOAc 4:1)
R_f = 0.63, stained by $KMnO_4$

^{1}H-NMR (500 MHz, acetone-d_6)
δ 7.75 (d, *J* = 8.5 Hz, 1H), 7.23 (d, *J* = 8.5 Hz, 1H), 3.84 (s, 3H), 2.47 (s, 3H), 2.45 (s, 3H).

^{13}C-NMR (125 MHz, acetone-d_6)
δ 168.7, 141.4, 138.7, 137.3, 132.5, 129.2, 106.9, 52.3, 26.0, 18.6

HRMS (EI+)
Calculated for $C_{10}H_{11}O_2I$: 289.98041
Found: 289.98135

IR (thin film, cm^{-1})
3347, 3261, 2926, 2357, 2341, 1722, 1567, 1527, 1433, 1398, 1230, 1248, 1159, 1097, 1051, 903, 816, 766, 669.

III. Synthesis of 3

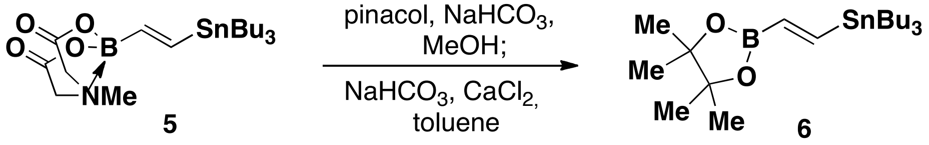

Pinacol boronic ester, 6. A 500 mL round bottom flask was charged with MIDA boronate **5**[4] (17.2 g, 36.4 mmol, 1.0 equiv.), pinacol (8.61 g, 72.9 mmol, 2,0 equiv), solid $NaHCO_3$ (15.3 g, 182.2 mmol, 5.0 equiv), and MeOH (180 mL). The reaction flask was topped with a septum and a gas inlet needle and flushed with N_2. The reaction mixture was placed in a 45 ^oC oil bath and maintained at that temperature with stirring for 3 h. The reaction mixture was cooled to 23 ^oC and filtered through a pad of Celite, rinsing with Et_2O. The collected solution was concentrated *in vacuo* in a 500 mL round bottom flask. The resulting residue was azeotroped with toluene to afford a clear oil. To remove residual pinacol, finely ground $CaCl_2$ (40.0 g, 364.4 mmol, 10 equiv.), solid $NaHCO_3$ (15.3 g, 182.2 mmol, 5.0 equiv), and toluene (200 mL) were added to the flask containing the crude material. The resulting suspension was stirred at 23 ^oC for 1 h and filtered through a pad of silica gel, rinsing with Et_2O. The collected solution was concentrated *in vacuo* to afford **6** as a clear oil (15.8 g, 98%).

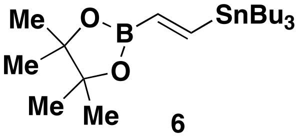

6

TLC (hexanes)

R_f = 0.38, stained by $KMnO_4$

^{1}H-NMR (500 MHz, acetone-d_6)

δ 7.52 (d, J = 22.0 Hz, 1H), 6.28 (d, J = 22.0 Hz, 1H), 1.54 (quint, J = 7.5 Hz, 6H), 1.33 (sext, J = 7.5 Hz, 6H), 1.23 (s, 12 H), 0.95 (t, J = 7.5 Hz, 6H), 0.89 (t, J = 7.5 Hz, 9 H).

^{13}C-NMR (125 MHz, acetone-d_6)

δ 159.1, 83.7, 27.9, 25.2, 13.9, 11.9, 9.9

^{11}B-NMR (128 MHz, acetone-d_6)

δ 28.0

HRMS (ESI+)

Calculated for $C_{20}H_{41}BO_2SnNa$:	467.2119
Found:	467.2108

IR (thin film, cm^{-1})

3205, 2977, 2958, 2927, 2871, 2854, 2362, 2341, 2100, 1641, 1573, 1369, 1328, 1265, 1145, 1018, 970, 848.

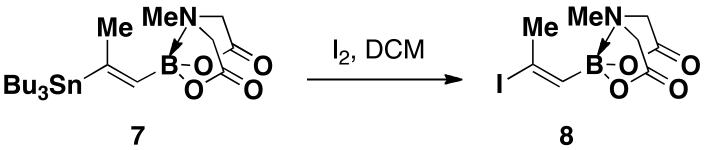

Vinyl iodide, 8. A 100 mL round bottom flask was charged with stannane **7**[5] (1.5 g, 3.0 mmol, 1 equiv.) and topped with a septum and a gas inlet needle. Dichloromethane (6 mL) was added to afford a pale yellow solution, and the flask was cooled to 0 °C. A 50 mL pear-shaped flask was charged with I_2 (822 mg, 3.24 mmol, 1.05 equiv.) and dichloromethane (29 mL). The I_2 solution was added dropwise to the reaction flask over the course of 1 h and stirred at 0 °C for an additional 15 min. The reaction mixture was quenched by the addition of saturated $Na_2S_2O_3$ solution (20 mL) and stirred at 10 °C until the reaction mixture became clear. The mixture was transferred to a separatory funnel, rinsing with EtOAc (70 mL) and brine (30 mL). The aqueous layer was extracted with EtOAc (3 x 50 mL) and the combined organic layers were dried over $MgSO_4$, filtered, and concentrated *in vacuo* to afford a white solid. The crude material was adsorbed onto Celite from an acetone solution and purified by SiO_2 chromatography (Et_2O → Et_2O:MeCN 10:1 → 7:1 → 5:1) to yield **8** as a white solid (725 mg, 75%).

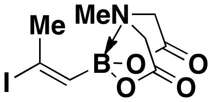

8

TLC (Et_2O:MeCN 4:1)
R_f = 0.4, stained by $KMnO_4$

^{1}H-NMR (500 MHz, acetone-d$_6$)
δ 6.22 (s, 1H), 4.25 (d, *J* = 17.0 Hz, 2H), 4.11 (d, *J* = 17.0 Hz, 2H), 3.12 (s, 3H), 2.61 (s, 3H).

^{13}C-NMR (125 MHz, acetone-d$_6$)
δ 168.6, 110.9, 62.5, 47.2, 33.2

^{11}B-NMR (128 MHz, acetone-d$_6$)
δ 9.58

HRMS (EI+)
Calculated for $C_8H_{11}BINO_4$: 321.98625
Found: 321.98653

IR (thin film, cm^{-1})
3008, 2972, 2951, 2916, 1757, 1620, 1466, 1450, 1429, 1340, 1290, 1124, 1090, 1066, 1001, 964, 887

X-ray quality crystals were grown by layering pentane on top of a solution of **8** in acetone.

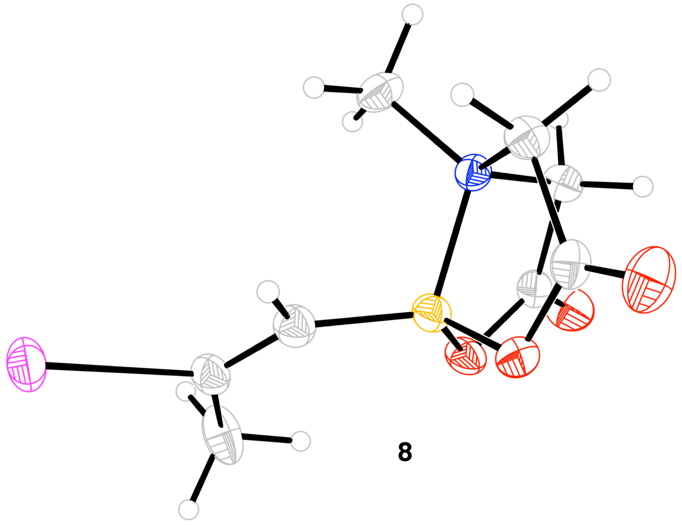

8

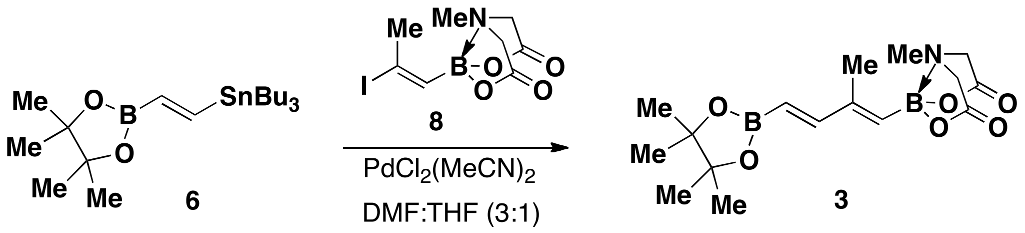

Bisborylated diene, 3. In a glovebox, to a 20 mL vial charged with stannane **6** (280.5 mg, 0.63 mmol, 1.3 equiv.) and iodide **8** (157 mg, 0.49 mmol, 1.0 equiv) was added *trans*-bis(acetonitrile)palladium dichloride (6.3 mg, 0.024 mmol, 5 mol%), DMF (7 mL) and THF (2.4 mL). The vial was sealed with a PTFE-lined cap and removed from the glovebox. The vial was placed in a 45 °C aluminum heat block and maintained at that temperature with stirring for 15 h. The reaction mixture was transferred into a separatory funnel containing brine (50 mL), rinsing with EtOAc (20 mL), and the layers were separated. The aqueous layer was extracted with EtOAc (2 x 30 mL). The combined organic layers were washed with brine:H_2O (1:1, 50 mL), dried over $MgSO_4$, filtered, and concentrated *in vacuo* to afford an off-white solid. The crude material was adsorbed onto Celite from an acetone solution and purified by SiO_2 chromatography (hexanes:EtOAc 1:1 → EtOAc) to afford **3** as a white solid (135 mg, 80%).

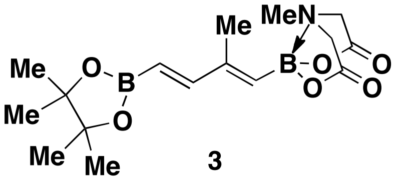

3

TLC (EtOAc)

R_f = 0.33, stained by $KMnO_4$

^{1}H-NMR (500 MHz, acetone-d_6)

δ 7.06 (d, *J* = 18.5 Hz, 1H), 5.64 (s, 1H), 5.54 (d, *J* = 18.0 Hz, 1H), 4.26 (d, *J* = 16.5 Hz, 2H), 4.09 (d, *J* = 17.0 Hz, 2H), 3.08 (s, 3H), 1.97 (s, 3H), 1.27 (s, 12H).

^{13}C-NMR (125 MHz, acetone-d_6)

δ 168.9, 157.3, 149.1, 83.7, 62.4, 47.1, 25.1, 14.8

^{11}B-NMR (128 MHz, acetone-d_6)

δ 30.3, 10.7

HRMS (ESI+)

Calculated for $C_{16}H_{25}$ B_2NO_6Na:	372.1766
Found:	372.1777

IR (thin film, cm^{-1})

2977, 2929, 2871, 2732, 2142.54, 1754, 1710, 1596, 1454, 1336, 1186, 1143, 995, 890, 850, 728, 651.

X-ray quality crystals were grown by layering ether on top of a solution of **3** in MeCN.

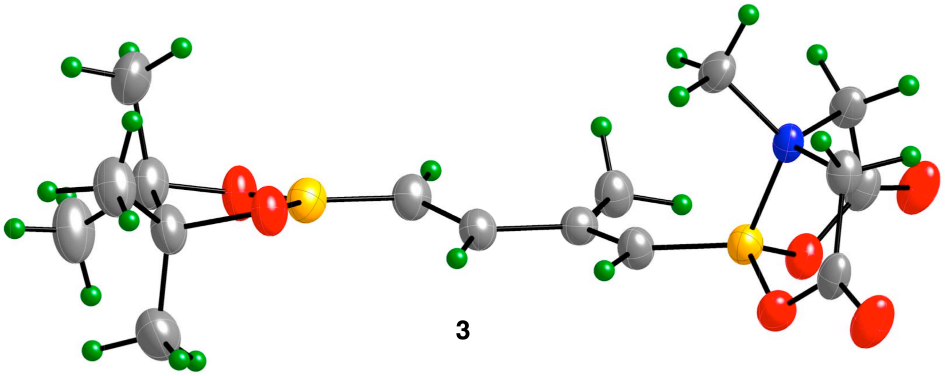

3

IV. Total synthesis of 1

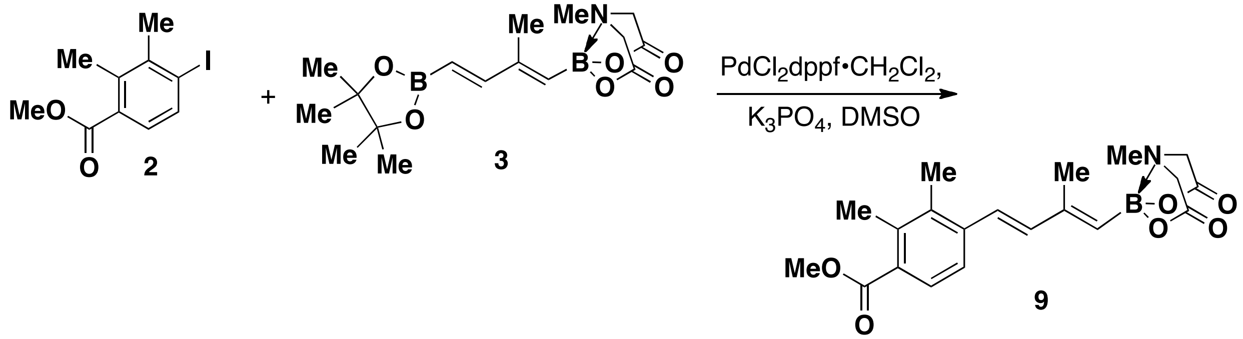

Dienyl MIDA boronate, 9. In a glovebox, to a 20 mL vial charged with **2** (240 mg, 0.83 mmol, 1.3 equiv.) and **3** (222.3 mg, 0.637 mmol, 1.0 equiv.) was added $PdCl_2dppf•CH_2Cl_2$ (26 mg, 0.032 mmol, 5 mol%), finely ground anhydrous K_3PO_4 (811 mg, 3.8 mmol, 6.0 equiv.), and DMSO (9 mL). The vial was sealed with a PTFE-lined cap and removed from the glove box. The vial was placed in a 45 °C aluminum heat block and maintained at that temperature with stirring for 24 h. The reaction was cooled to 23 °C and transferred to a separatory funnel, diluting with EtOAc (20 mL). The organic layer was washed with brine:H_2O (1:1, 2 x 40 mL) to remove DMSO, dried over $MgSO_4$, filtered, and concentrated *in vacuo* to afford a yellow solid. The crude material was adsorbed onto Celite from an acetone solution and purified by SiO_2 chromatography (hexanes:EtOAc 1:1 → EtOAc) to afford **9** as a pale yellow solid (171 mg, 70%).

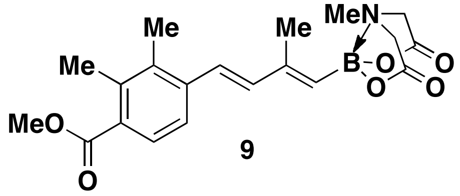

9

TLC (EtOAc)

R_f = 0.39, stained by $KMnO_4$

^{1}H-NMR (500 MHz, acetone-d_6)

δ 7.55 (d, *J* = 8.0 Hz, 1H), 7.41 (d, *J* = 8.0 Hz, 1H), 6.94 (d, *J* = 16.0 Hz, 1H), 6.82 (d, *J* = 15.5 Hz, 1H), 5.62, (s, 1H), 4.24 (d, *J* = 17.0 Hz, 2H), 4.07 (d, *J* = 17.0 Hz, 2H), 3.83 (s, 3H), 3.01 (s, 3H), 2.45 (s, 3H), 2.33 (s, 3H), 2.10 (s, 3H).

^{13}C-NMR (125 MHz, acetone-d_6)

δ 169.3, 169.0, 148.5, 141.0, 140.4, 138.3, 136.4, 128.0, 126.6, 123.8, 117.5, 62.4, 52.1, 47.1, 17.2, 15.8, 15.6

^{11}B-NMR (128 MHz, acetone-d_6)

δ 10.9

HRMS (ESI+)

Calculated for $C_{20}H_{25}BNO_6$: 386.1775

Found: 386.1779

IR (thin film, cm^{-1})

3220, 3178, 3139, 3006, 2964, 2927, 2127, 1760, 1710, 1637, 1600, 1448, 1286, 1245, 1151, 983, 889.

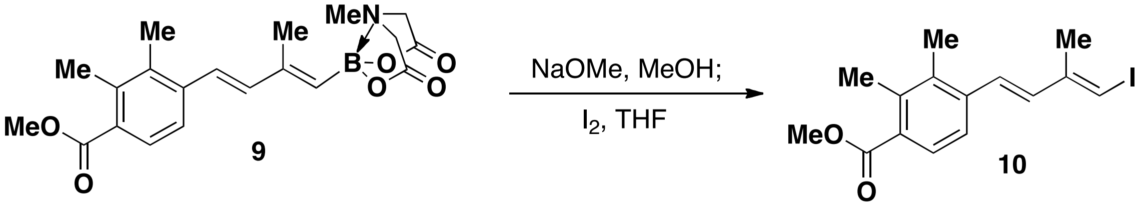

Dienyl iodide, 10. In a glovebox, a 7 mL vial was charged with NaOMe (67.9 mg, 1.26 mmol, 5.0 equiv.). The vial was sealed with a PTFE-lined septum cap and removed from the glove box, and MeOH (2.0 mL) and THF (1.0 mL) were added to afford a clear solution. A separate 7 mL vial was charged with I_2 (192 mg, 0.756 mmol, 3.0 equiv) and THF (3.8 mL, 0.2M with respect to I_2). A 20 mL vial was charged with **9** (96.9 mg, 0.252 mmol, 1.0 equiv.) and THF (2.0 mL). The NaOMe solution was added dropwise to the reaction vial over 5 min. The reaction mixture was vigorously stirred at 23 ^oC. TLC analysis indicated complete deprotection of the MIDA boronate to the boronic acid in 10 min. The I_2 solution was dropwise added to the vigorous stirring mixture and the resulting dark purple mixture was stirred at 23 ^oC for 20 min. The reaction mixture was quenched with pH7 phosphate buffer (20 mL) and transferred to a separatory funnel, rinsing with EtOAc (20 mL) and the layers were separated. The organic layer was washed with saturated $Na_2S_2O_3$ (2 x 20 mL) and the aqueous layer was back-extracted with EtOAc (10 mL). The combined organic layers were dried over $MgSO_4$, filtered, and concentrated *in vacuo* to afford **10** as a yellow solid (89 mg, 99%).

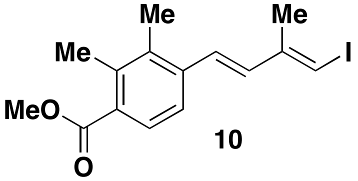

TLC (EtOAc)

R_f = 0.90, stained by $KMnO_4$

^{1}H-NMR (500 MHz, acetone-d_6)

δ 7.55 (d, J = 8.0 Hz, 1H), 7.42 (d, J = 8.0 Hz, 1H), 7.06 (d, J = 16.0 Hz, 1H), 6.94 (d, J = 15.5 Hz, 1H), 6.77, (s, 1H), 3.83 (s, 3H), 2.44 (s, 3H), 2.32 (s, 3H), 2.13 (s, 3H).

^{13}C-NMR (125 MHz, acetone-d_6)

δ 169.2, 146.3, 140.3, 138.4, 136.5, 133.6, 131.3, 128.0, 127.9, 123.7, 86.2, 52.1, 20.2, 17.2, 15.9

HRMS (ESI+)

Calculated for $C_{15}H_{18}O_2I$: 357.0352

Found: 357.0345

IR (thin film, cm^{-1})

3216, 3116, 2950, 2360, 2341, 1718, 1643, 1592, 1432, 1407, 1380, 1282, 1243, 1207, 1187, 1147, 1066, 1037, 960.

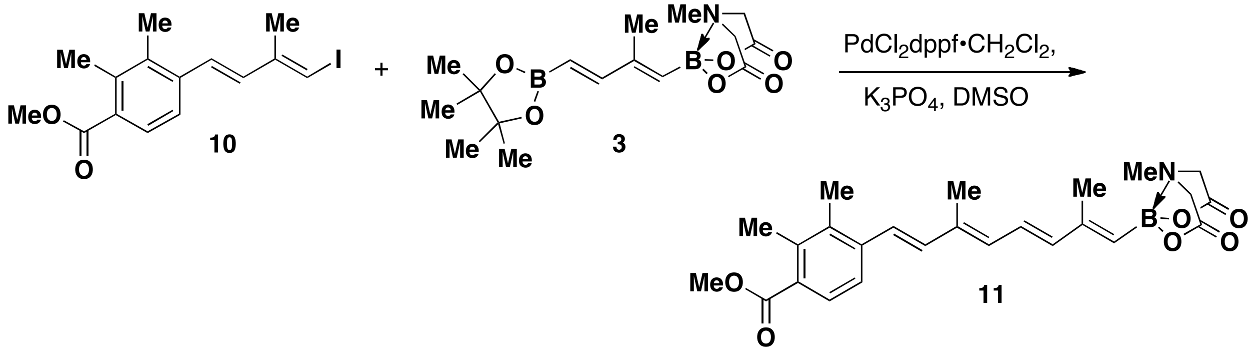

Tetraenyl MIDA boronate, 11. In a glovebox, to a 7 mL vial charged with **10** (125.4 mg, 0.35 mmol, 1.3 equiv.) and **3** (94.5 mg, 0.27 mmol, 1.0 equiv.) was added $PdCl_2dppf•CH_2Cl_2$ (11.1 mg, 0.014 mmol, 5 mol%), finely ground anhydrous K_3PO_4 (345 mg, 1.62 mmol, 6.0 equiv.), and DMSO (3.9 mL). The vial was sealed with a PTFE-lined cap and removed from the glove box. The vial was placed in a 45 ^oC aluminum heat block and maintained at that temperature with stirring for 24 h. The reaction was cooled to 23 ^oC and transferred to a separatory funnel, diluting with EtOAc (20 mL). The organic layer was washed with brine:H_2O (1:1, 2 x 40 mL) to remove DMSO, dried over $MgSO_4$, filtered, and concentrated *in vacuo* to afford a yellow-orange solid. The crude material was adsorbed onto Celite from an acetone solution and purified by SiO_2 chromatography (hexanes:EtOAc 1:1 → EtOAc) to afford **11** as a yellow solid (78.9 mg, 65%).

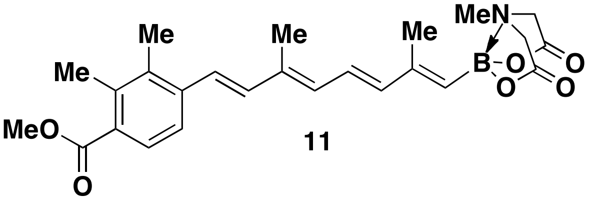

11

TLC (EtOAc)

R_f = 0.39, stained by $KMnO_4$

^{1}H-NMR (500 MHz, acetone-d_6)

δ 7.56 (d, *J* = 8.5 Hz, 1H), 7.46 (d, *J* = 8.5 Hz, 1H), 6.96 (d, *J* = 15.5 Hz, 1H), 6.90 (d, *J* = 16.0 Hz, 1H), 6.75, (dd, *J* = 15.0 Hz, 11.0 Hz, 1H), 6.46 (d, *J* = 14.5 Hz, 1H), 6.40 (d, *J* = 12.0 Hz, 1H), 5.49 (s, 1H), 4.22 (d, *J* = 17.0 Hz, 2H), 4.05 (d, *J* = 17.0 Hz, 2H), 3.83 (s, 3H), 3.04 (s, 3H) 2.45 (s, 3H), 2.34 (s, 3H), 2.08 (s, 3H), 2.03 (s, 3H).

^{13}C-NMR (125 MHz, acetone-d_6)

δ 168.7, 168.3, 148.4, 141.6, 140.4, 137.8, 137.2, 136.2, 135.5, 133.8, 130.2, 127.4, 125.5, 125.0, 122.9, 61.8, 51.4, 46.5, 16.7, 15.2, 15.0, 12.3

^{11}B-NMR (128 MHz, acetone-d_6)

δ 11.1

HRMS (ESI+)

Calculated for $C_{25}H_{31}BNO_6$: 452.2244
Found: 452.2263

IR (thin film, cm^{-1})

3012, 2962, 2595, 2098, 1760, 1710, 1643, 1450, 1340, 1288, 1243, 1189, 1151, 1093, 1066, 1024, 985, 964, 889.

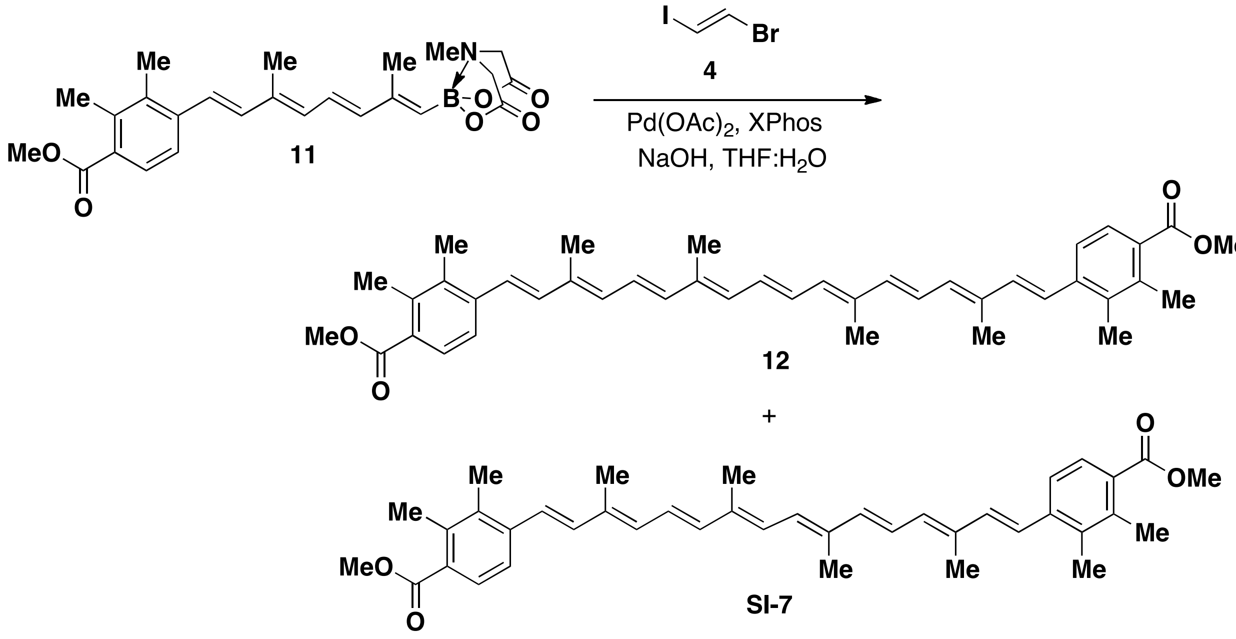

Synechoxanthin dimethyl ester, 12.

Preparation of stock solutions. In a glovebox, a 7 mL vial was added XPhos and THF (8.6 mg/mL) to afford a clear solution. To another 7 mL vial was added $Pd(OAc)_2$ and THF (2.2 mg/mL) to afford a pale yellow-orange solution. To a separate 7 mL vial was added **4**[3] and THF (21.1 mg/mL).

The freshly prepared stock solutions were used in the following reaction:

In a glovebox, to a 7 mL vial charged with MIDA boronate **11** (100 mg, 0.22 mmol, 2.2 equiv.) was added solid NaOH (56 mg, 1.4 mmol, 14.0 equiv.) and THF (0.56 mL). The stock solution of **4** in THF (1.11 mL) was added to the reaction vial followed by the XPhos stock solution (0.56 mL, which contains 4.7 mg, 0.01 mmol, 10 mol%) and the $Pd(OAc)_2$ stock solution (0.56 mL, which contains 1.2 mg, 0.005 mmol, 5 mol%). The vial was sealed with a PTFE-lined septum cap,

removed from the glovebox, and stirred at 23 °C for 5 min. Degassed DI H_2O (0.66 mL, 0.15 M) was added dropwise. The solution was stirred in a subdued light environment at 23 °C for 1 h. The reaction mixture was quenched with pH 7 phosphate buffer (10 mL) and transferred to a separatory funnel, rinsing with Et_2O (10 mL). The layers were separated and the aqueous layer was extracted with Et_2O (2 x 10 mL), dried over $MgSO_4$, filtered, and concentrated *in vacuo* to afford a bright orange-red solid. The resulting residue was adsorbed onto Celite from an acetone solution and purified by SiO_2 chromatography (hexanes:EtOAc 20:1 → 10:1) to afford a mixture of synechoxanthin dimethyl ester **12** as a red solid (32.6 mg, 53%) and byproduct **SI-7** (13.1 mg, 21%). Due to difficulty in separating **SI-7** from **12**, the semi-purified mixture was carried forward to the deprotection step.

For characterization of **SI-7**, see page S21-22.

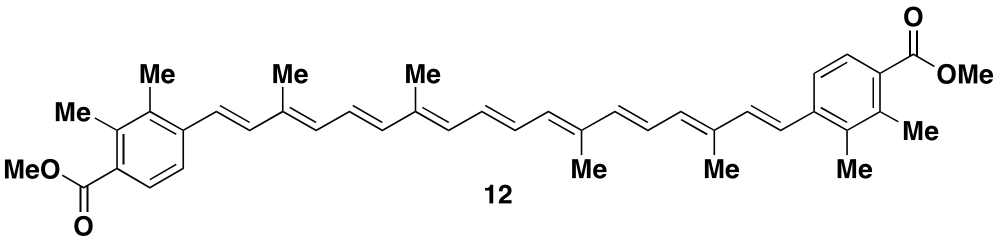

12

TLC (hexanes:EtOAc 4:1)

R_f = 0.57, visualized by visible light (orange)

^{1}H-NMR (500 MHz, CD_2Cl_2)

δ 7.57 (d, *J* = 8.5 Hz, 2H), 7.40 (d, *J* =8.5 Hz, 2H), 6.89 (d, *J* = 16.0 Hz, 2H), 6.83 (d, *J* = 16.0 Hz, 2H), 6.73-6.69 (m, 4H), 6.47 (d, *J* =15.0 Hz, 2H), 6.39 (d, *J* =11.5 Hz, 2H), 6.33 (d, *J* =10.0 Hz, 2H), 3.86 (s, 6H) 2.47 (s, 6H), 2.34 (s, 6H), 2.09 (s, 6H), 2.01 (s, 6H).

^{13}C-NMR (125 MHz, CD_2Cl_2)

169.3, 140.5, 139.0, 138.2, 137.2, 137.2, 136.2, 135.8, 134.3, 133.6, 130.8, 130.1, 127.6, 125.7, 125.4, 122.9, 52.2, 17.4, 15.9, 13.1, 12.9

HRMS (ESI+)

Calculated for $C_{42}H_{49}O_4$:	617.3631
Found:	617.3625

IR (thin film, cm^{-1})

3280, 3174, 3066, 2960, 2237, 1643 (broad), 1413

^{13}C NMR data for synechoxanthin dimethyl ester δ_C/ppm

Natural synechoxanthin dimethyl ester **15** (literature reference, 600 MHz, CD_2Cl_2)[7]	Synthetic synechoxanthin dimethyl ester **15** (500 MHz, CD_2Cl_2)
169.3	169.3
140.7	140.5
139.2	139.0
138.2	138.2
137.2	137.2
137.2	137.2
136.4	136.2
135.7	135.8
134.3	134.3
133.7	133.6
130.9	130.8
130.1	130.1
127.6	127.6
125.7	125.7
125.4	125.4
123.0	122.9
52.2	52.2
17.5	17.4
16.0	15.9
13.2	13.1
13.1	12.9

[7] J. E. Graham, J. T. Lecomte, D. A. Bryant, *J. Nat. Prod.* **2008**, *71*, 1647-1650.

^1H NMR data for synechoxanthin dimethyl ester δ_H/ppm

Natural synechoxanthin dimethyl ester **15** (literature reference, 600 MHz, CD_2Cl_2)[7]	Synthetic synechoxanthin dimethylester **15** (500 MHz, CD_2Cl_2)
7.57	7.57
7.40	7.40
6.89	6.89
6.83	6.83
6.73	6.73-6.69
6.69	6.73-6.69
6.47	6.47
6.39	6.39
6.33	6.33
3.86	3.86
2.47	2.47
2.33	2.34
2.08	2.09
2.01	2.01

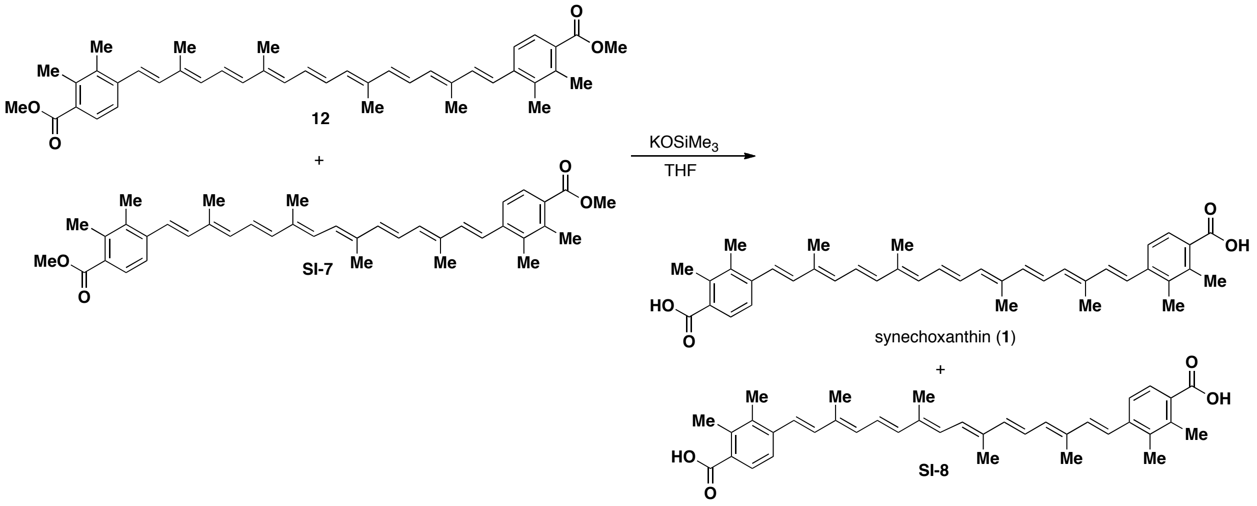

Synechoxanthin, (1). In a glovebox, to a 20 mL vial charged with a mixture of synechoxanthin dimethyl ester **12** and byproduct **SI-7** (45.7 mg, 0.073 mmol, 1.0 equiv.) was added $KOSiMe_3$ (43 mg, 1.46 mmol, 20 equiv.) and THF (7.3 mL, 0.01 M). The vial was sealed with a PTFE-lined cap and removed from the glove box. The vial was placed in a 65 °C aluminum heat block and maintained at that temperature with stirring for 1 h. The reaction mixture was cooled to 23 °C and concentrated *in vacuo* to afford a bright red solid. The crude material was filtered through a small pad of celite with MeOH and DMSO and purified by preparatory HPLC (70% MeOH in 25

mM NH_4OAc buffer → 95% MeOH in 25 mM NH_4OAc buffer) to afford **SI-8** as a bright red solid (7.6 mg, 64%) and synechoxanthin (**1**) as a bright red solid (16.6 mg, 53%).

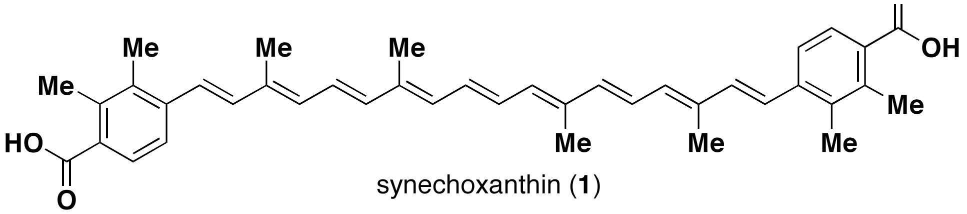

synechoxanthin (**1**)

TLC (hexanes:EtOAc:AcOH 4:1:0.5)

R_f = 0.43, visualized by visible light (orange)

HPLC

tR = 10.2 min; flow rate = 25 mL/min, gradient of 70% → 95% MeOH in 25 mM NH_4OAc buffer over 10 min followed by 95% MeOH in 25 mM NH_4OAc buffer over 10 min.

^{1}H-NMR (500 MHz, CD_3OD:DMSO-d_6, 1:1)

δ 7.37 (d, J = 8.0 Hz, 2H), 7.28 (d, J = 7.5 Hz, 2H), 6.91 (d, J = 16.0 Hz, 2H), 6.84 (d, J = 16.0 Hz, 2H), 6.78-6.73 (m, 4H), 6.49 (d, J = 15.0 Hz, 2H), 6.49 (d, J = 11.5 Hz, 2H), 6.39 (d, J = 8.0 Hz, 2H), 2.37 (s, 6H), 2.30 (s, 6H), 2.07 (s, 6H), 2.00 (s, 6H). The proton signal for the carboxylic acid (2H) was not observed.

^{13}C-NMR (150 MHz, DMSO-d_6)

δ 16.9, 15.3, 12.7, 12.5 (partial assignment due to low solubility of **1** in DMSO)

HRMS (ESI+)

Calculated for $C_{40}H_{44}O_4Na$:	611.3137
Found:	611.3130

IR (thin film, cm^{-1})

3461, 3419, 3365, 3234, 3089, 2919, 2360, 2132, 1751, 1644, 1047, 1025, 991.

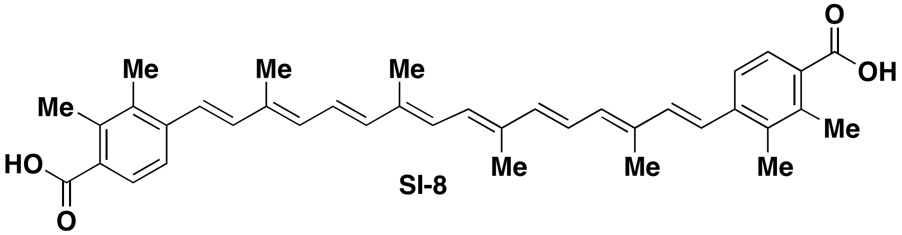

SI-8

TLC (hexanes:EtOAc:AcOH 4:1:0.5)

R_f = 0.43, visualized by visible light (orange)

HPLC

tR = 9.4 min; flow rate = 25 mL/min, gradient of 70% → 95% MeOH in 25 mM NH_4OAc buffer over 10 min followed by 95% MeOH in 25 mM NH_4OAc buffer over 10 min.

^{1}H-NMR (500 MHz, CD_3OD: DMSO-d_6, 1:1)

δ 7.37 (d, *J* = 8.0 Hz, 2H), 7.27 (d, *J* = 8.0 Hz, 2H), 6.91 (d, *J* = 15.5 Hz, 2H), 6.85 (d, *J* = 15.5 Hz, 2H), 6.79-6.74 (m, 2H), 6.60 (s, 2H), 6.57 (d, *J* = 15.0 Hz, 2H), 6.43 (d, *J* = 11.0 Hz, 2H), 2.38 (s, 6H), 2.29 (s, 6H), 2.07 (s, 6H), 2.01 (s, 6H). The proton signal for the carboxylic acid (2H) was not observed.

HRMS (ESI+)

Calculated for $C_{38}H_{42}O_4Na$:	585.2981
Found:	585.2979

In order to verify the structure of byproduct **SI-7**, **SI-8** was subjected to methylation:

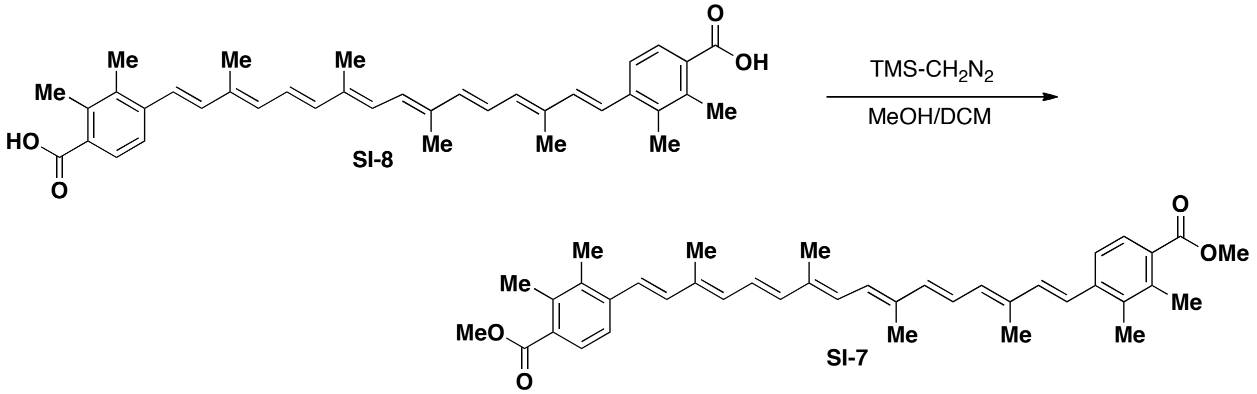

Byproduct methyl ester, SI-7. A 1.5-mL vial was charged with **SI-8** (3 mg, 0.005 mmol, 1.0 equiv.) and flushed with N_2. MeOH:DCM (1:1. 0.5 mL) was added and the reaction vial was cooled to 0 °C. TMS-diazomethane (2.0 M in ether, 0.2 mL) was added dropwise and stirred at 0 °C for 30 min. The reaction was warmed to 23 °C for 1 h. The reaction was quenched by the addition of a few drops of acetic acid and the mixture was concentrated *in vacuo* to afford a bright red solid. The crude material was dissolved in dichloromethane and filtered through a plug of Celite and silica gel and concentrated *in vacuo* to afford **SI-7** as a bright red solid (2 mg, 64%).

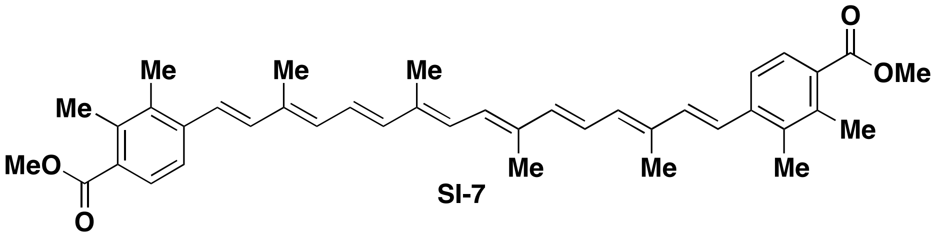

SI-7

TLC (hexanes:EtOAc 4:1)

R_f = 0.57, visualized by visible light (orange)

^{1}H-NMR (500 MHz, CD_2Cl_2)

δ 7.57 (d, *J* = 8.5 Hz, 2H), 7.40 (d, *J* =8.0 Hz, 2H), 6.89 (d, *J* = 16.0 Hz, 2H), 6.84 (d, *J* = 15.5 Hz, 2H), 6.74 (dd, *J* = 15.0 Hz, *J* = 11.5 Hz, 2H), 6.55 (s, 2H), 6.53 (d, *J* =15.0 Hz, 2H), 6.40 (d, *J* =11.5 Hz, 2H), 3.86 (s, 6H) 2.47 (s, 6H), 2.33 (s, 6H), 2.09 (s, 6H), 2.03 (s, 6H).

^{13}C-NMR (125 MHz, CD_2Cl_2)

169.3, 140.6, 139.5, 138.3, 137.5, 137.2, 136.2, 135.8, 134.3, 130.1, 129.4, 127.6, 125.7, 125.3, 122.9, 52.1, 17.4, 15.9, 13.1, 12.9

HRMS (ESI+)

Calculated for $C_{40}H_{46}O_4Na$:	613.3294
Found:	613.3291

IR (thin film, cm^{-1})

2920, 2848, 2364, 2341, 1714, 1589, 1554, 1431, 1236, 1146, 957.

sfIV98crude

exp1 s2pul

SAMPLE		DEC. & VT	
date	Nov 11 2009	dfrq	499.432
solvent	CDCl3	dn	H1
file	exp	dpwr	25
ACQUISITION		dof	0
sfrq	499.432	dm	nnn
tn	H1	dmm	c
at	4.096	dmf	200
np	65536	dseq	
sw	8000.0	dres	1.0
fb	4000	homo	n
bs	4	DEC2	
tpwr	62	dfrq2	0
pw	6.2	dn2	
d1	0	dpwr2	1
tof	0	dof2	0
nt	4	dm2	n
ct	4	dmm2	c
alock	n	dmf2	200
gain	not used	dseq2	
FLAGS		dres2	1.0
il	n	homo2	n
in	n	PROCESSING	
dp	y	lb	0.30
hs	nn	wtfile	
DISPLAY		proc	ft
sp	-499.1	fn	not used
wp	5497.6	math	f
vs	151		
sc	0	werr	
wc	250	wexp	
hzmm	21.99	wbs	
is	33.57	wnt	
rfl	5135.3		
rfp	3625.9		
th	2		
ins	1.000		
nm	ph		

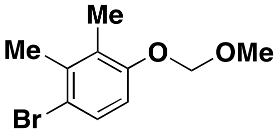

SI-2

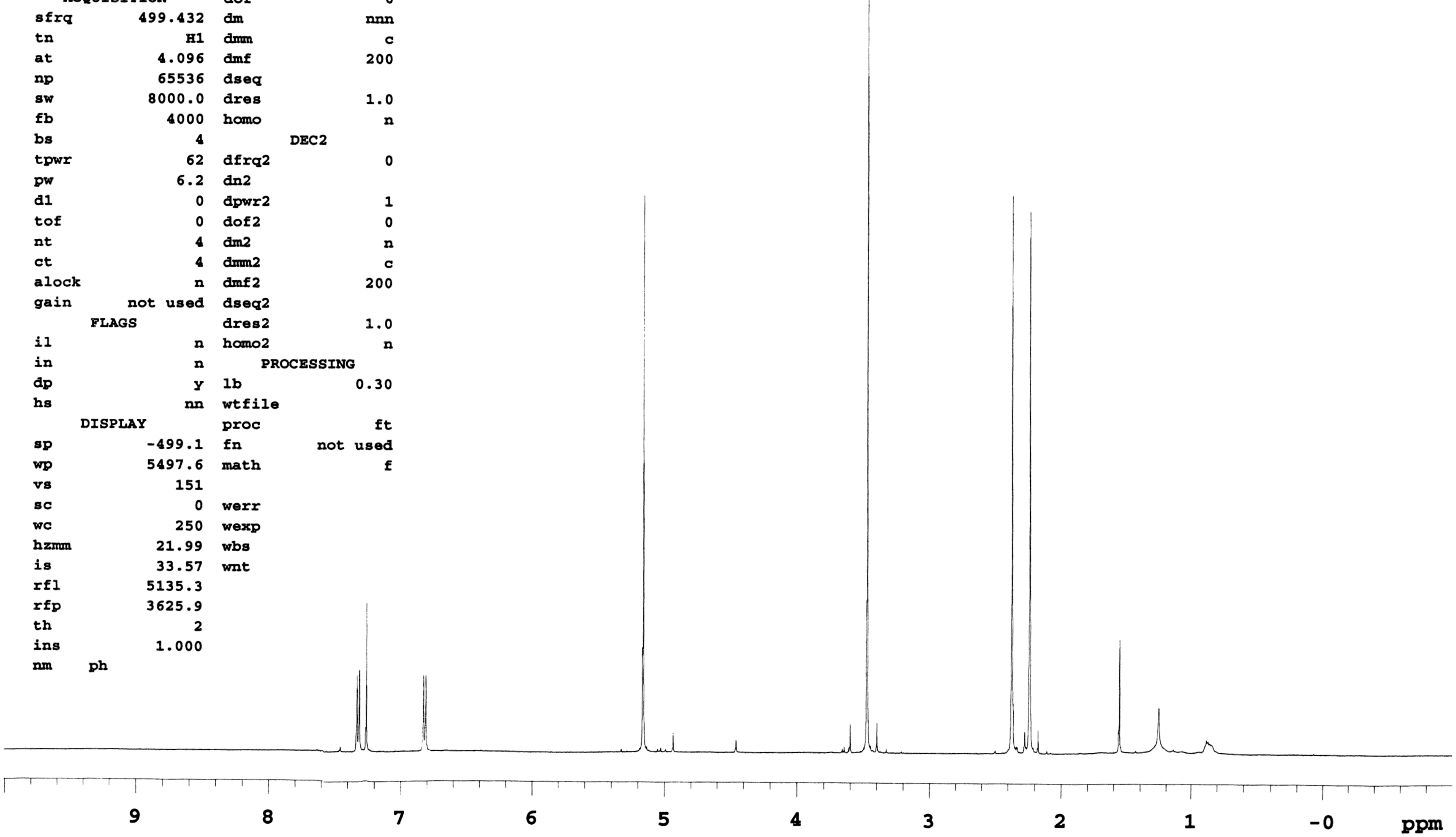

aryl bromide

Pulse Sequence: s2pul

Solvent: CDCl3
Ambient temperature
User: 1-14-87
File: SYC2NII31-C13
INOVA-500 "sunds1"

Relax. delay 1.000 sec
Pulse 65.9 degrees
Acq. time 1.086 sec
Width 30165.9 Hz
368 repetitions
OBSERVE C13, 125.6473175 MHz
DECOUPLE H1, 499.6923275 MHz
Power 44 dB
continuously on
WALTZ-16 modulated
DATA PROCESSING
Line broadening 1.0 Hz
FT size 65536
Total time 34 min, 57 sec

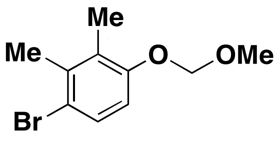

SI-2

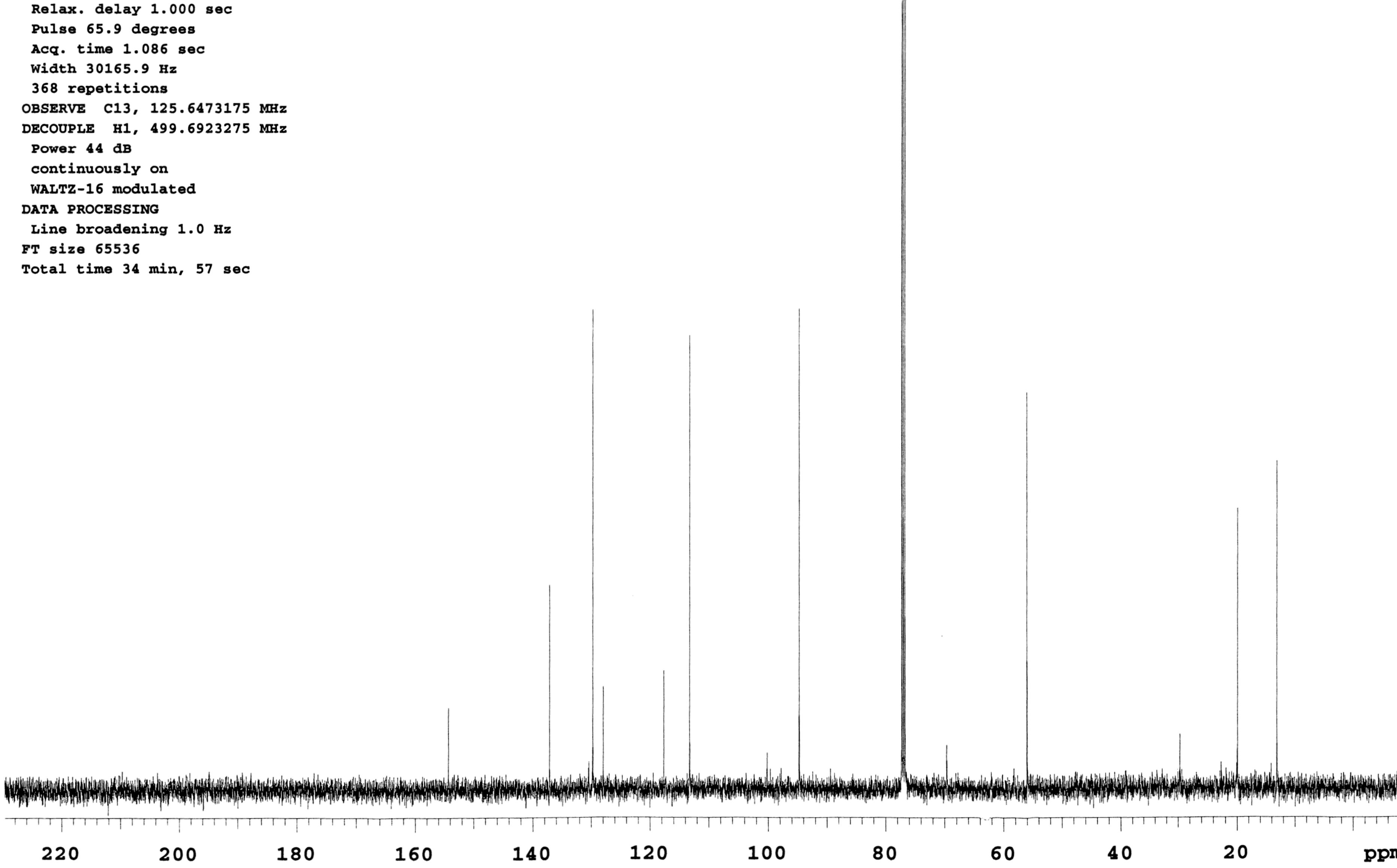

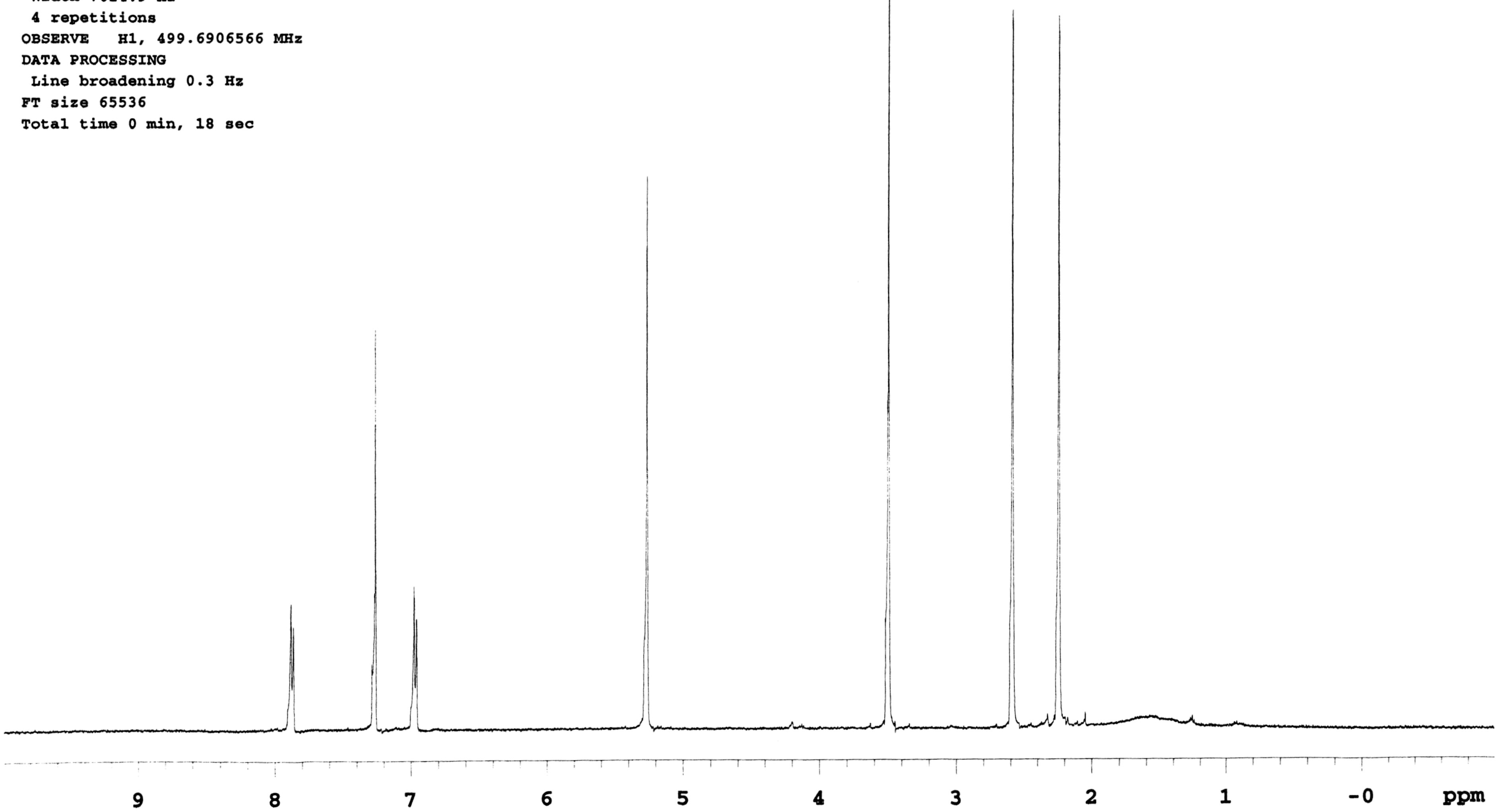
sf-acid-OMOM
Pulse Sequence: s2pul
Solvent: CDCl3
Ambient temperature
File: sf-IX-31-hexanewash
INOVA-500 "sunds1"
Pulse 61.6 degrees
Acq. time 4.665 sec
Width 7024.9 Hz
4 repetitions
OBSERVE H1, 499.6906566 MHz
DATA PROCESSING
Line broadening 0.3 Hz
FT size 65536
Total time 0 min, 18 sec
9
8
7
6
5
4
3
2
1
-0
ppm

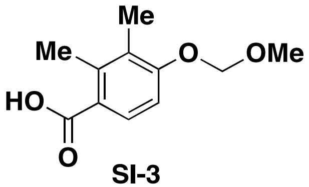
Me
Me
O
OMe
HO
O

SI-3

acid-OMOM

Pulse Sequence: s2pul

Solvent: CDCl3
Ambient temperature
User: 1-14-87
File: SYC3TII33-C13
INOVA-500 "sunds1"

Relax. delay 1.000 sec
Pulse 45.0 degrees
Acq. time 1.024 sec
Width 32000.0 Hz
416 repetitions
OBSERVE C13, 125.5817492 MHz
DECOUPLE H1, 499.4315638 MHz
Power 49 dB
continuously on
WALTZ-16 modulated
DATA PROCESSING
Line broadening 1.0 Hz
FT size 65536
Total time 33 min, 54 sec

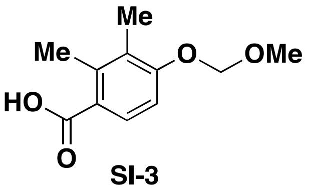

SI-3

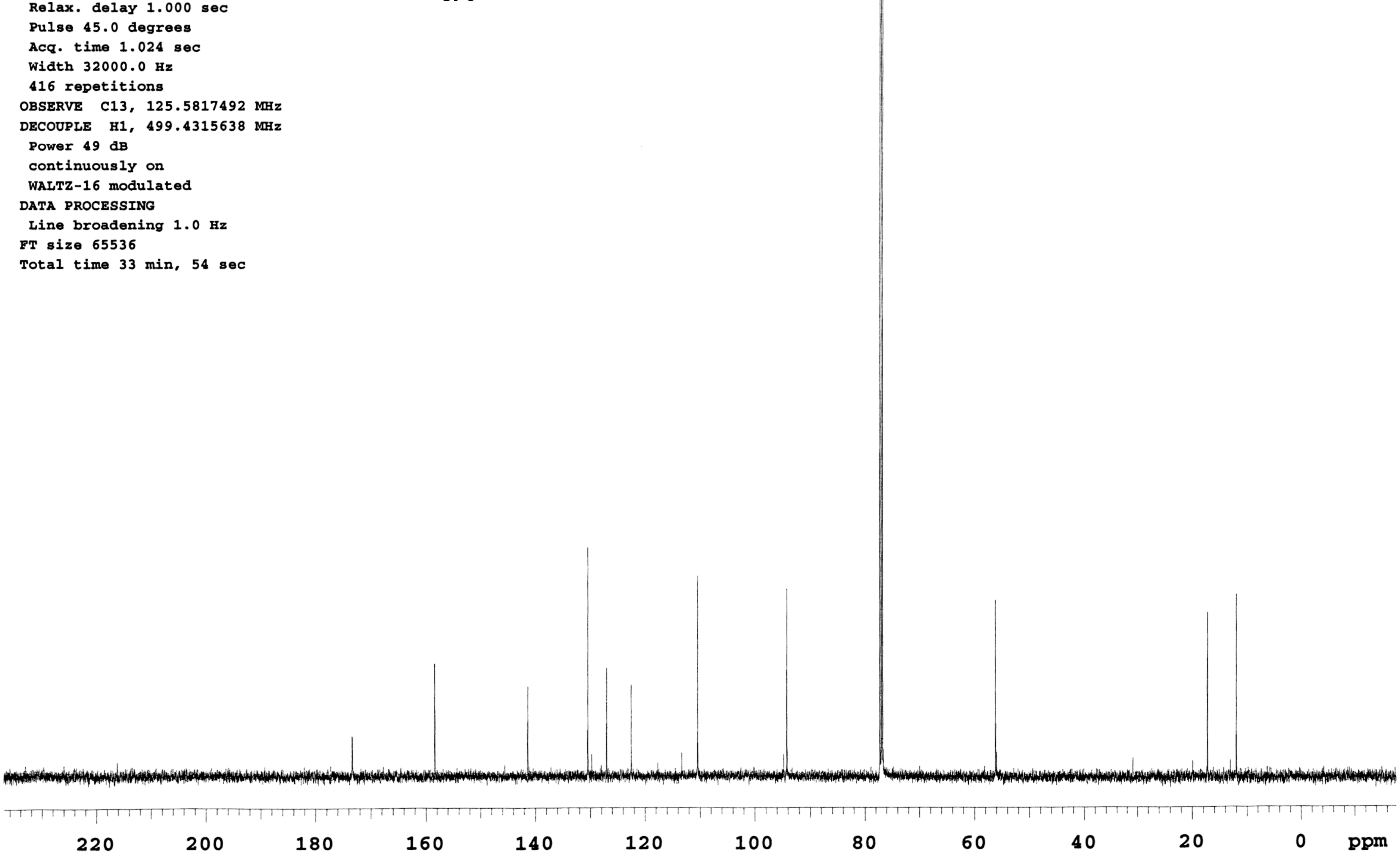

benzoate

Pulse Sequence: s2pul

Solvent: CDCl3
Ambient temperature
File: sfIV58crude
INOVA-500 "sunds1"

Pulse 45.0 degrees
Acq. time 4.096 sec
Width 8000.0 Hz
8 repetitions
OBSERVE H1, 499.4298999 MHz
DATA PROCESSING
Line broadening 0.3 Hz
FT size 65536
Total time 0 min, 32 sec

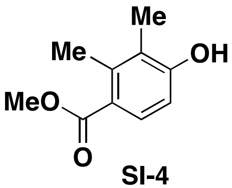

SI-4

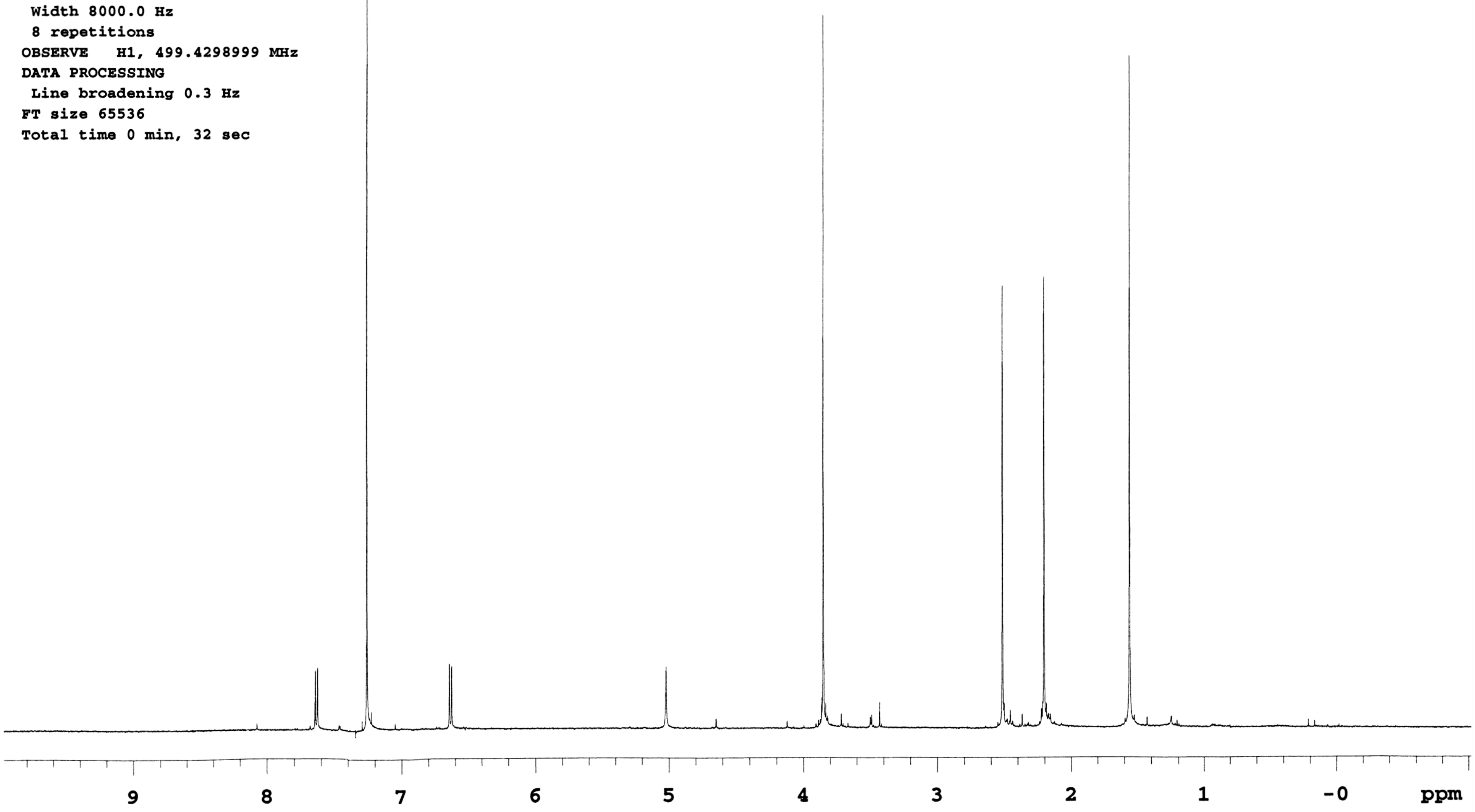

9/22/10,chang,mdb,SYC4II139-C13

Pulse Sequence: s2pul

Solvent: CDCl3
Ambient temperature
User: 1-14-87
File: SYC4II139-C13
INOVA-500 "sunds1"

Relax. delay 1.000 sec
Pulse 65.9 degrees
Acq. time 1.086 sec
Width 30165.9 Hz
384 repetitions
OBSERVE C13, 125.6472855 MHz
DECOUPLE H1, 499.6923275 MHz
Power 44 dB
continuously on
WALTZ-16 modulated
DATA PROCESSING
Line broadening 1.0 Hz
FT size 65536
Total time 34 min, 57 sec

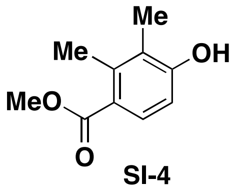

SI-4

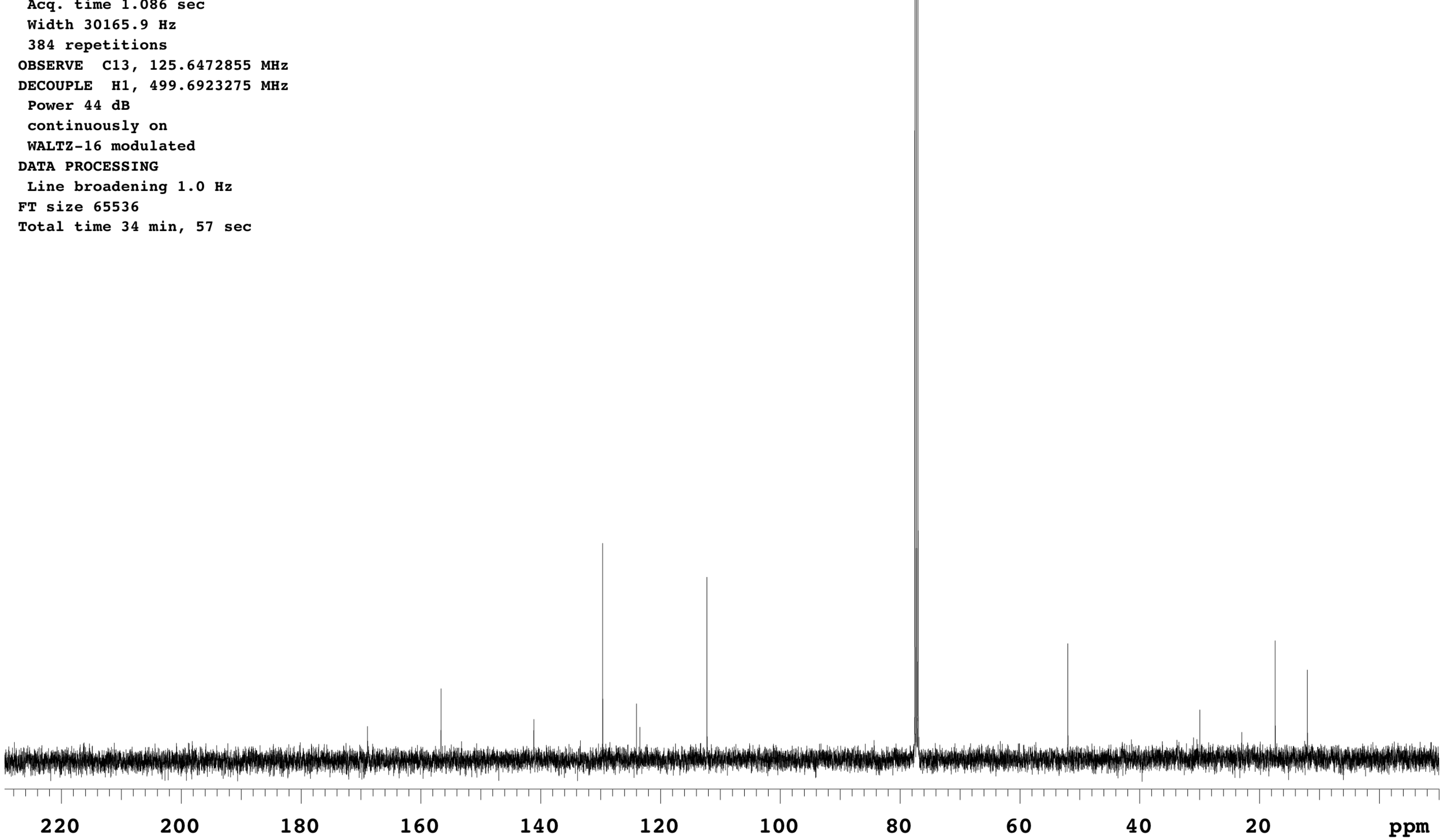

triflate bb1

Pulse Sequence: s2pul

Solvent: CDCl3
Ambient temperature
File: sf-vii-71-f15-18
INOVA-500 "sunds1"

Pulse 45.0 degrees
Acq. time 4.096 sec
Width 8000.0 Hz
4 repetitions
OBSERVE H1, 499.4298999 MHz
DATA PROCESSING
Line broadening 0.3 Hz
FT size 65536
Total time 0 min, 16 sec

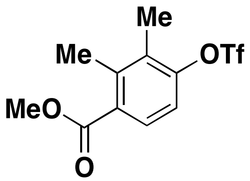

SI-5

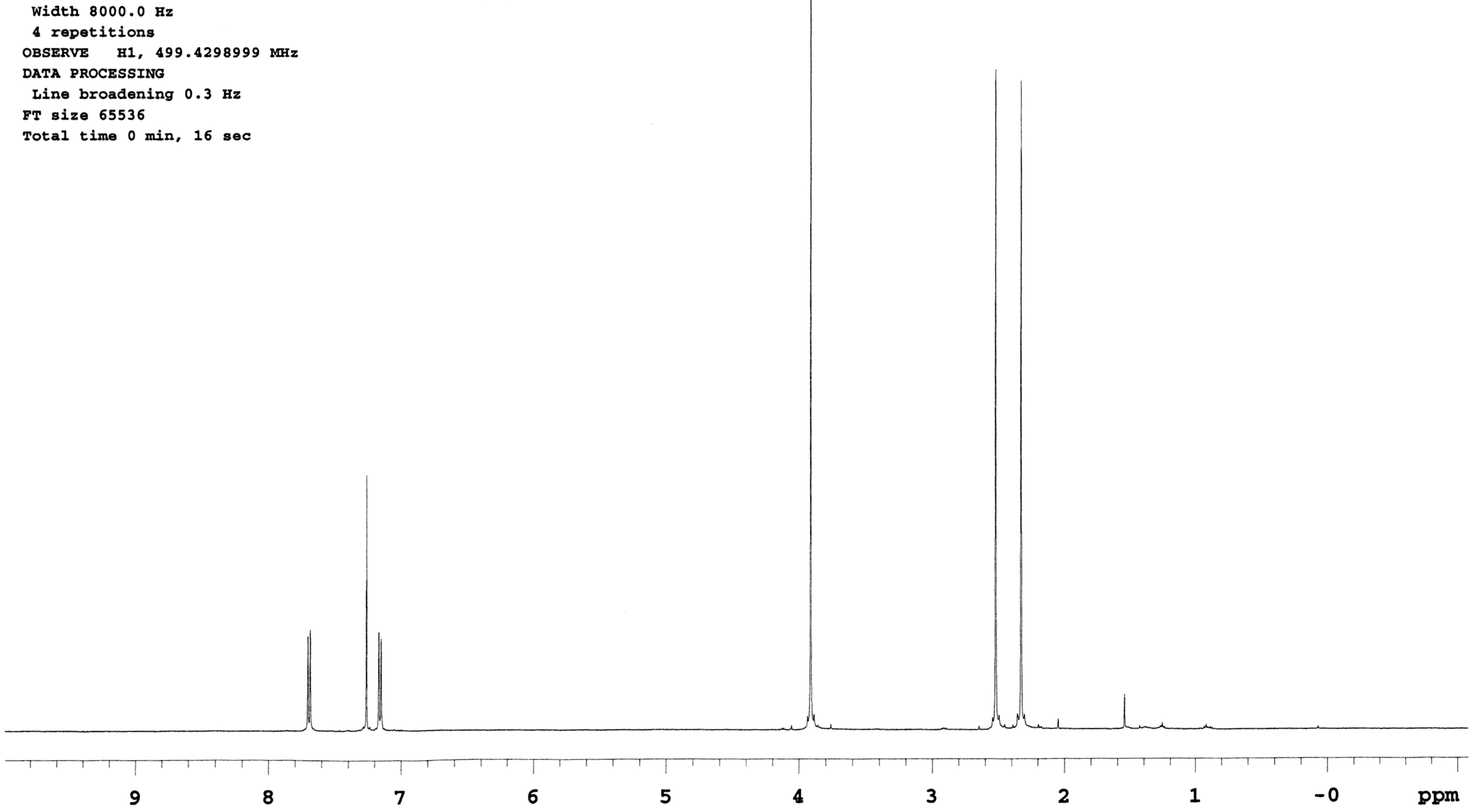

9/22/10,chang,mdb,SYC5KI143-C13

Pulse Sequence: s2pul

Solvent: CDCl3
Ambient temperature
User: 1-14-87
File: SYC5KI143-C13
INOVA-500 "sunds1"

Relax. delay 1.000 sec
Pulse 65.9 degrees
Acq. time 1.086 sec
Width 30165.9 Hz
272 repetitions
OBSERVE C13, 125.6472855 MHz
DECOUPLE H1, 499.6923275 MHz
Power 44 dB
continuously on
WALTZ-16 modulated
DATA PROCESSING
Line broadening 1.0 Hz
FT size 65536
Total time 34 min, 57 sec

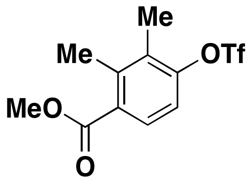

SI-5

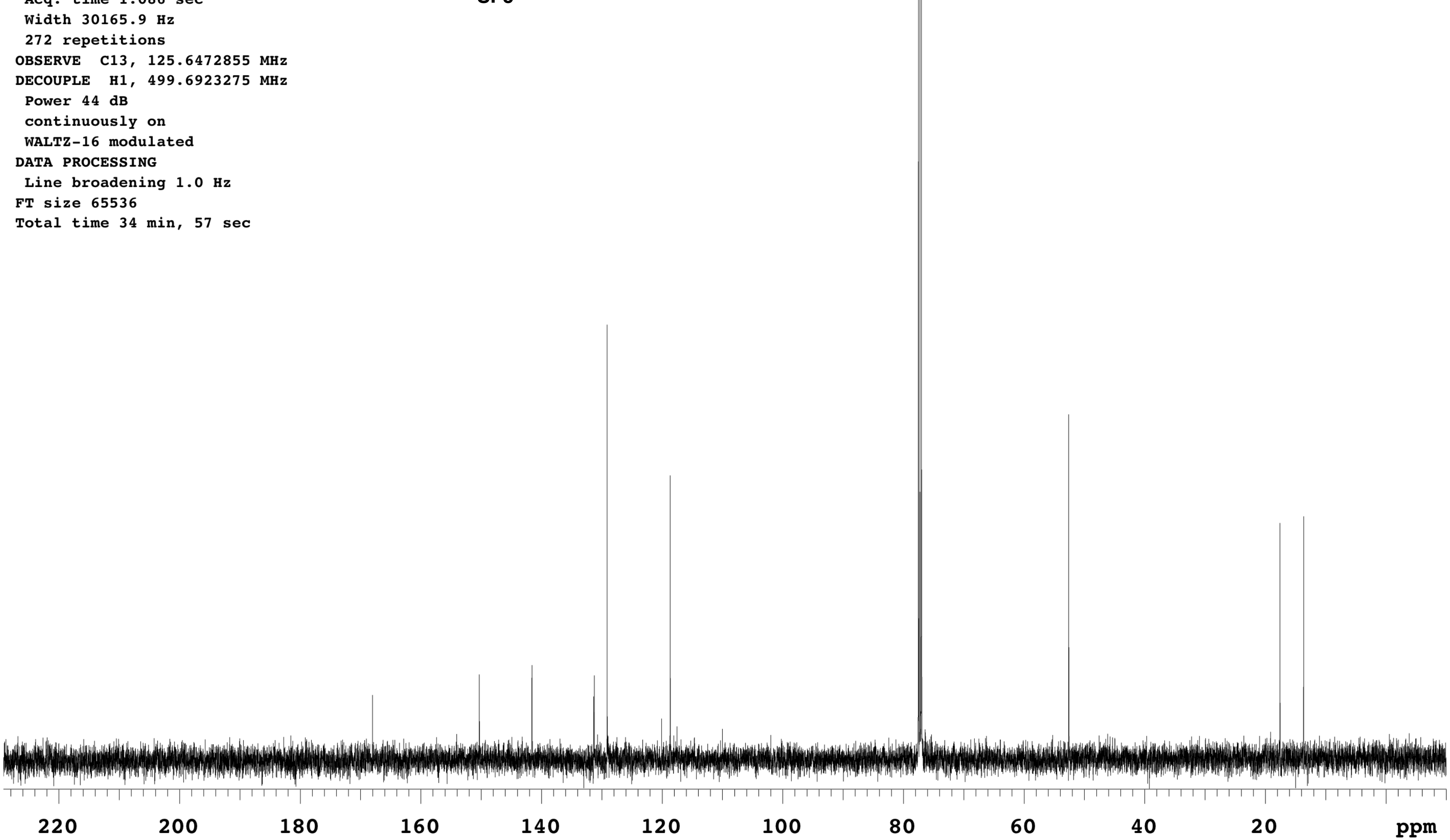

neopentylglycol_boronic_ester

Pulse Sequence: s2pul

Solvent: CDCl3
Ambient temperature
File: SYC11EII81mplc22-28
INOVA-500 "sunds1"

Pulse 61.6 degrees
Acq. time 4.665 sec
Width 7024.9 Hz
10 repetitions
OBSERVE H1, 499.6906579 MHz
DATA PROCESSING
Line broadening 0.3 Hz
FT size 65536
Total time 0 min, 46 sec

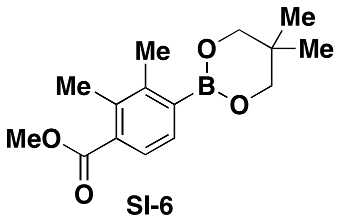

SI-6

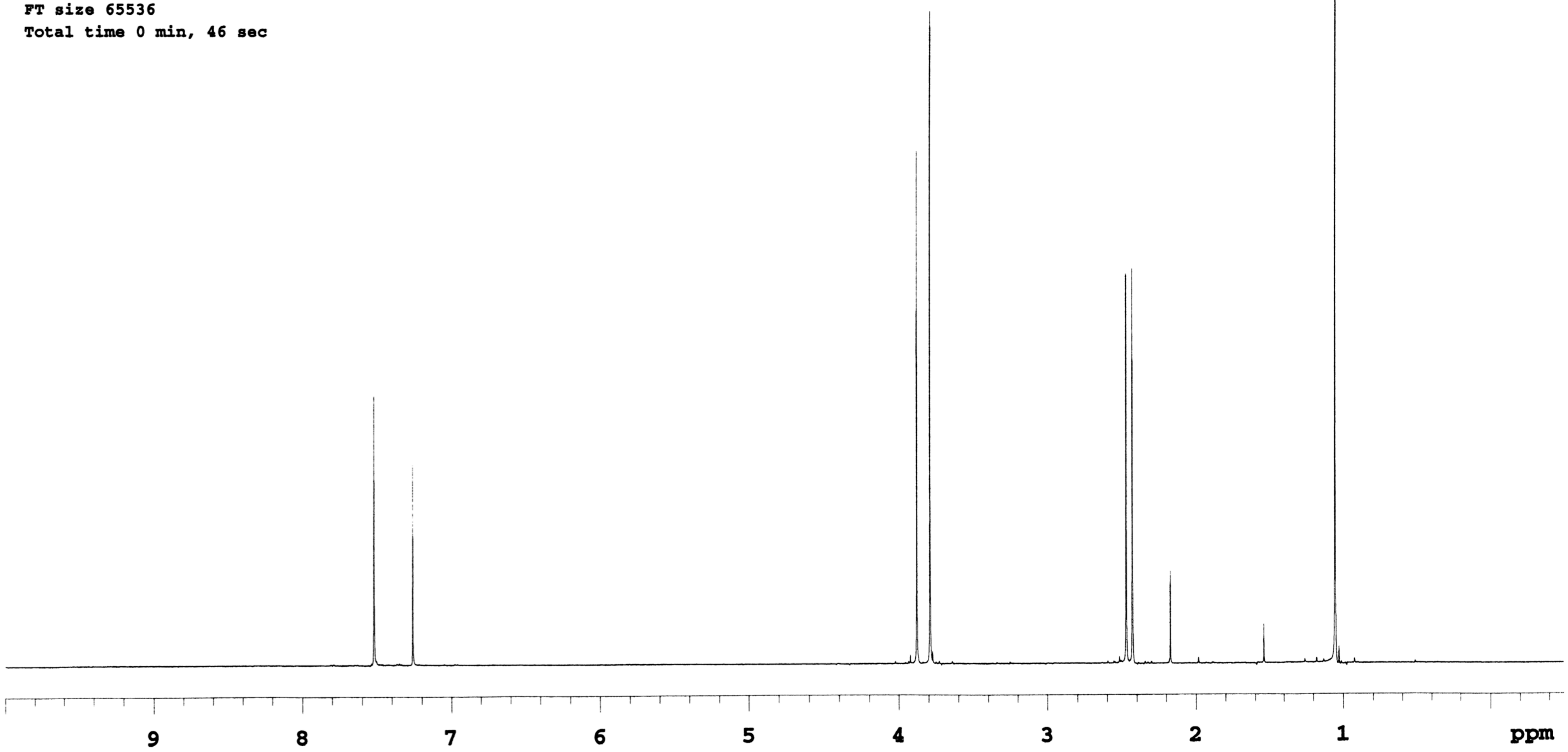

neopentylglycol_boronic_ester

Pulse Sequence: s2pul

Solvent: CDCl3
Ambient temperature
User: 1-14-87
File: SYC11EII81-13C
INOVA-500 "sunds1"

Relax. delay 1.000 sec
Pulse 65.9 degrees
Acq. time 1.086 sec
Width 30165.9 Hz
320 repetitions
OBSERVE C13, 125.6473083 MHz
DECOUPLE H1, 499.6923275 MHz
Power 44 dB
continuously on
WALTZ-16 modulated
DATA PROCESSING
Line broadening 1.0 Hz
FT size 65536
Total time 34 min, 57 sec

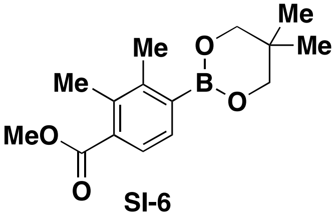

SI-6

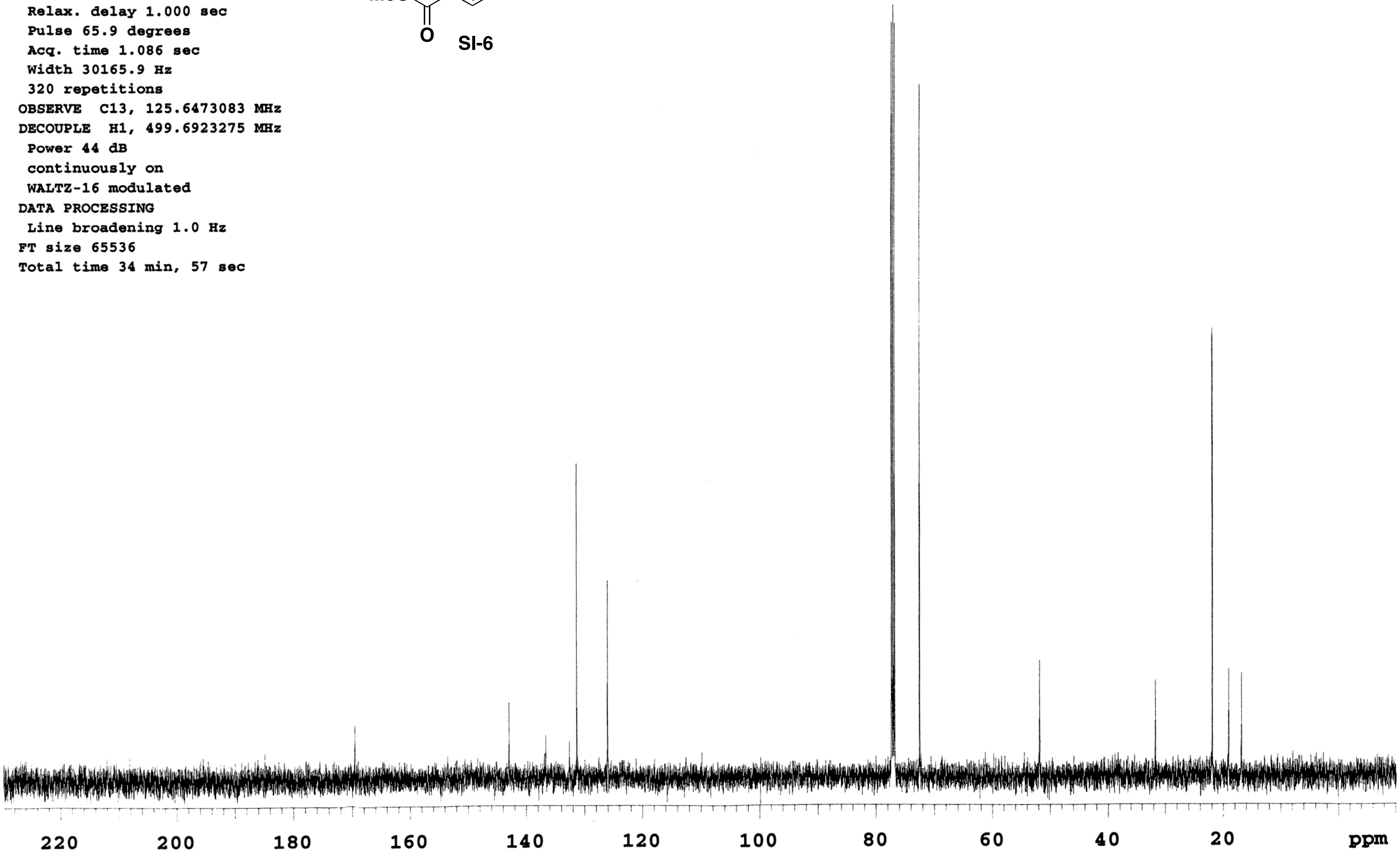

sf-xi-61-f14-27

Pulse Sequence: s2pul

Solvent: Acetone
Ambient temperature
INOVA-500 "vxr500"

Pulse 45.0 degrees
Acq. time 4.096 sec
Width 8000.0 Hz
4 repetitions
OBSERVE H1, 499.4324979 MHz
DATA PROCESSING
Line broadening 0.3 Hz
FT size 65536
Total time 0 min, 16 sec

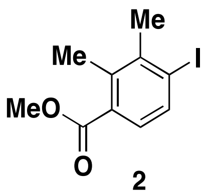

2

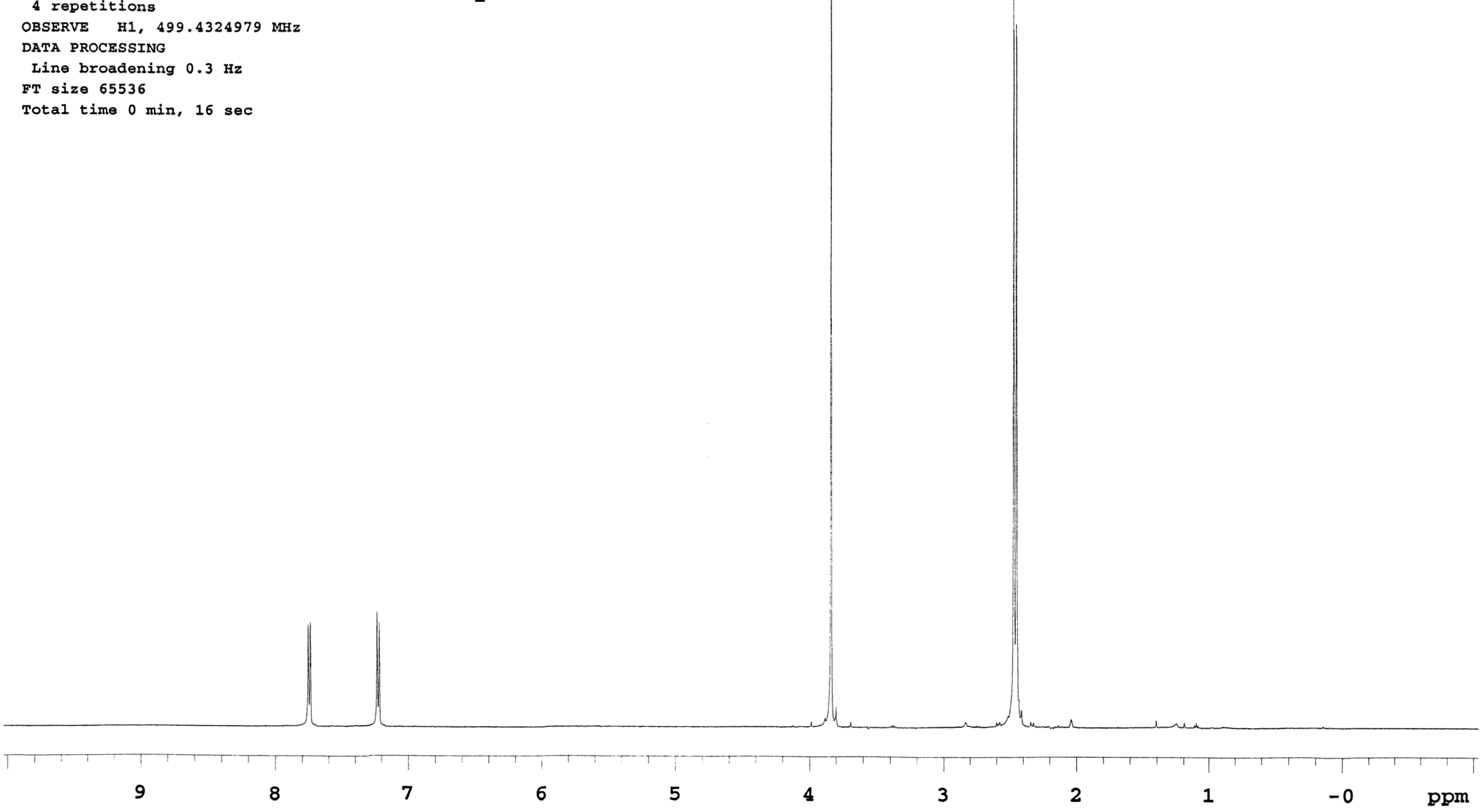

sf-xi-61-f14-27-13C

exp1 s2pul

SAMPLE		DEC. & VT	
date	Feb 4 2011	dfrq	499.434
solvent	Acetone	dn	H1
file	exp	dpwr	48
ACQUISITION		dof	-827.0
sfrq	125.596	dm	yyy
tn	C13	dmm	w
at	1.024	dmf	18519
np	65536	dseq	
sw	32000.0	dres	1.0
fb	18000	homo	n
bs	16	DEC2	
ss	1	dfrq2	0
tpwr	63	dn2	
pw	4.2	dpwr2	1
d1	1.000	dof2	0
tof	1880.0	dm2	n
nt	1000	dmm2	c
ct	160	dmf2	18519
alock	n	dseq2	
gain	not used	dres2	1.0
FLAGS		homo2	n
il	n	PROCESSING	
in	n	lb	1.00
dp	y	wtfile	
hs	nn	proc	ft
DISPLAY		fn	not used
sp	-2127.8	math	f
wp	31999.0		
vs	162	werr	
sc	0	wexp	
wc	250	wbs	
hzmm	6.37	wnt	
is	500.00		
rfl	5871.1		
rfp	3742.4		
th	25		
ins	100.000		
nm	ph		

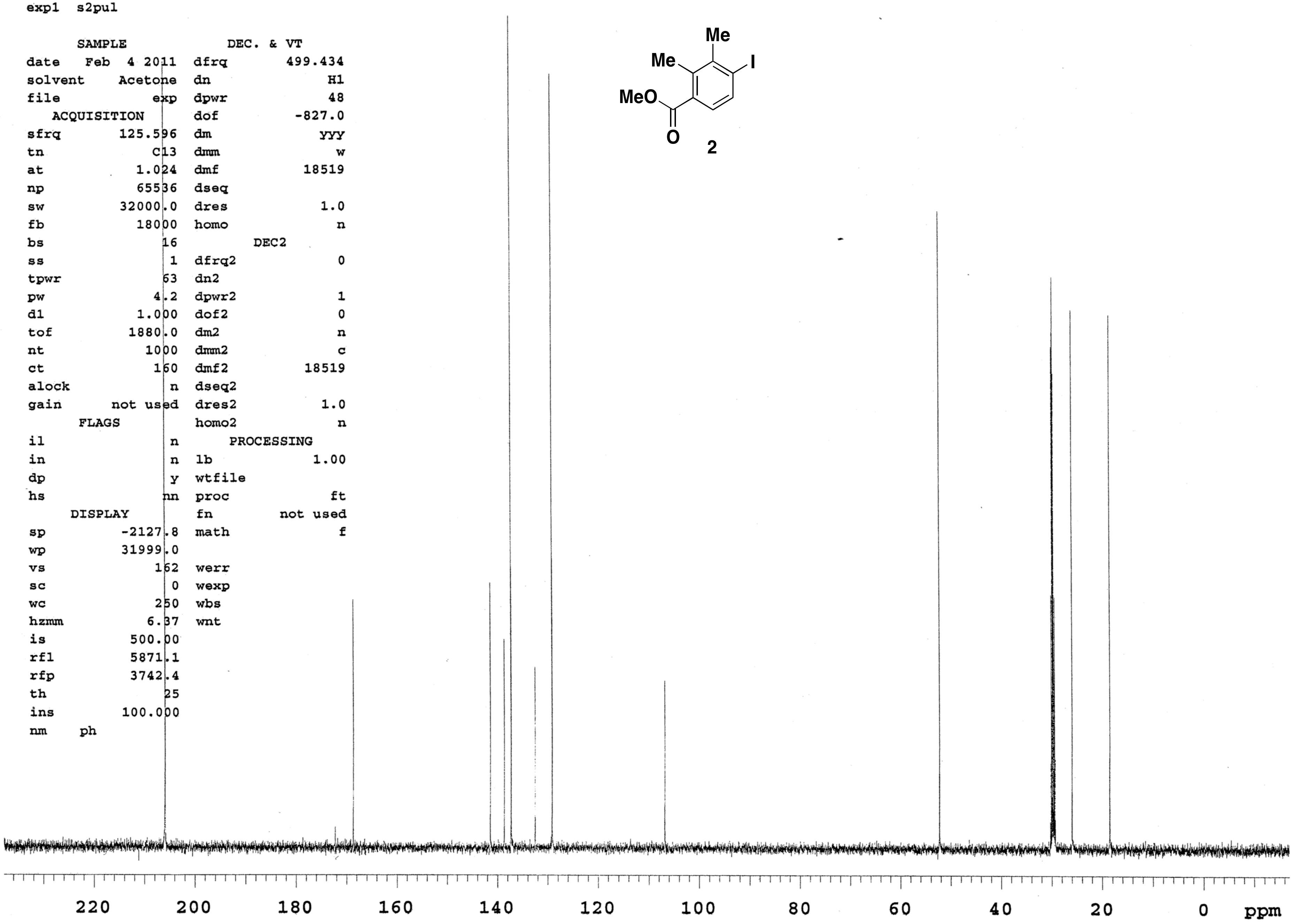

BPin-Sn-ethene

exp1 s2pul

SAMPLE		DEC. & VT	
date	Jun 24 2010	dfrq	500.073
solvent	Acetone	dn	H1
file /export/home/~		dpwr	18
data/ui500nb/Burke~		dof	0
/fujiis/sf-viii-73~		dm	nnn
-column2-f3-10.fid		dmm	c
ACQUISITION		dmf	200
sfrq	500.073	dseq	
tn	H1	dres	1.0
at	4.096	homo	n
np	65536	PROCESSING	
sw	8000.0	lb	0.30
fb	4000	wtfile	
bs	16	proc	ft
tpwr	55	fn	not used
pw	9.0	math	f
d1	0		
tof	0	werr	
nt	4	wexp	
ct	4	wbs	
alock	n	wnt	
gain	not used		
FLAGS			
il	n		
in	n		
dp	y		
hs	nn		
DISPLAY			
sp	-533.3		
wp	5551.8		
vs	56		
sc	0		
wc	250		
hzmm	22.21		
is	33.57		
rfl	2537.4		
rfp	1020.1		
th	1		
ins	1.000		
ai cdc ph			

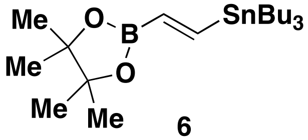

6

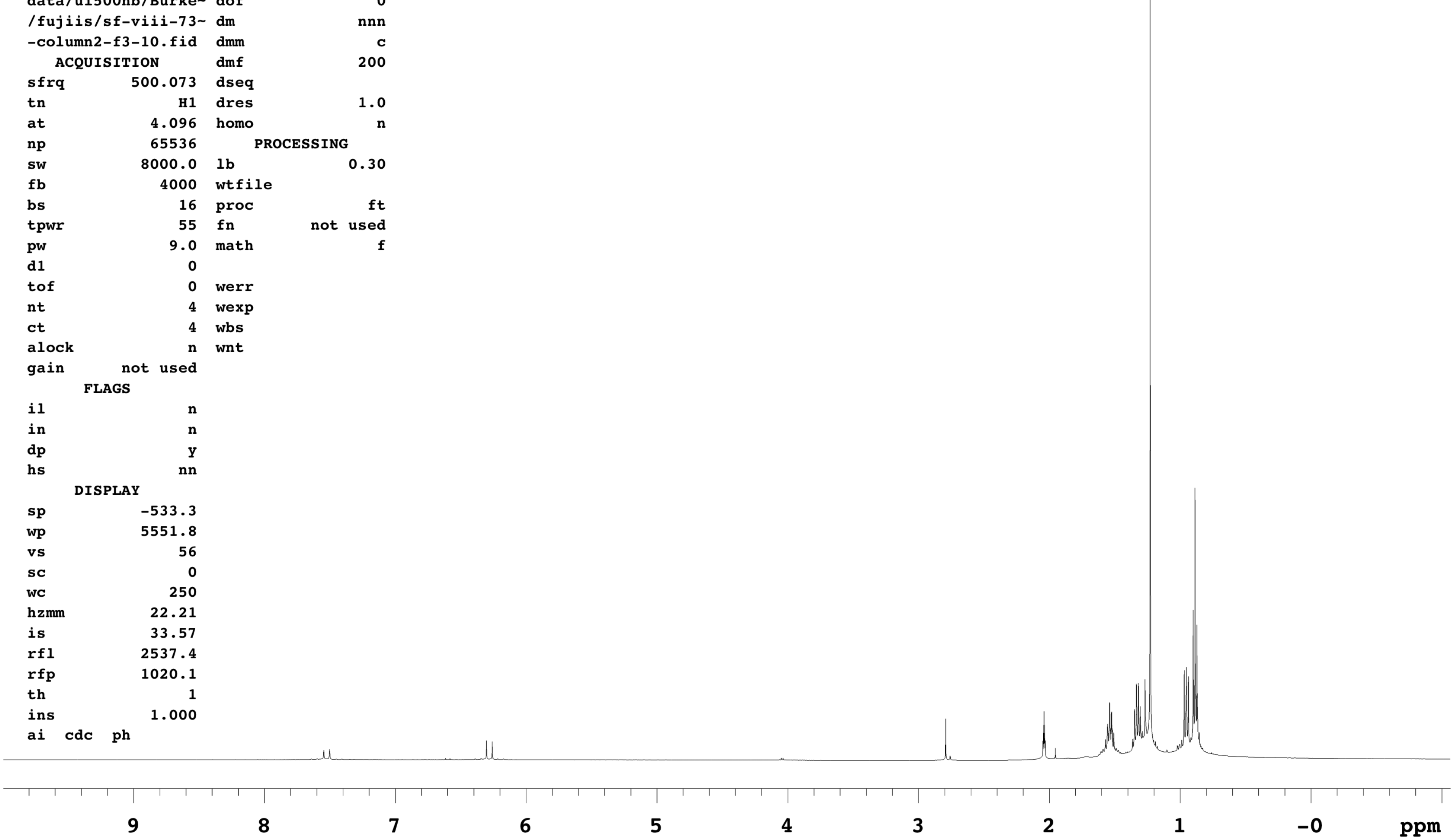

Bpin-Sn-ethene

Pulse Sequence: s2pul

Solvent: Acetone
Ambient temperature
User: 1-14-87
File: BPin-Sn-ethene
INOVA-500 "sunds1"

Relax. delay 1.000 sec
Pulse 45.0 degrees
Acq. time 1.024 sec
Width 32000.0 Hz
192 repetitions
OBSERVE C13, 125.5822914 MHz
DECOUPLE H1, 499.4341558 MHz
Power 49 dB
continuously on
WALTZ-16 modulated
DATA PROCESSING
Line broadening 1.0 Hz
FT size 65536
Total time 564 hr, 41 min, 45 sec

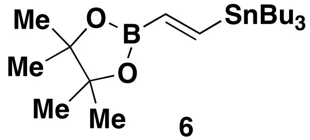

6

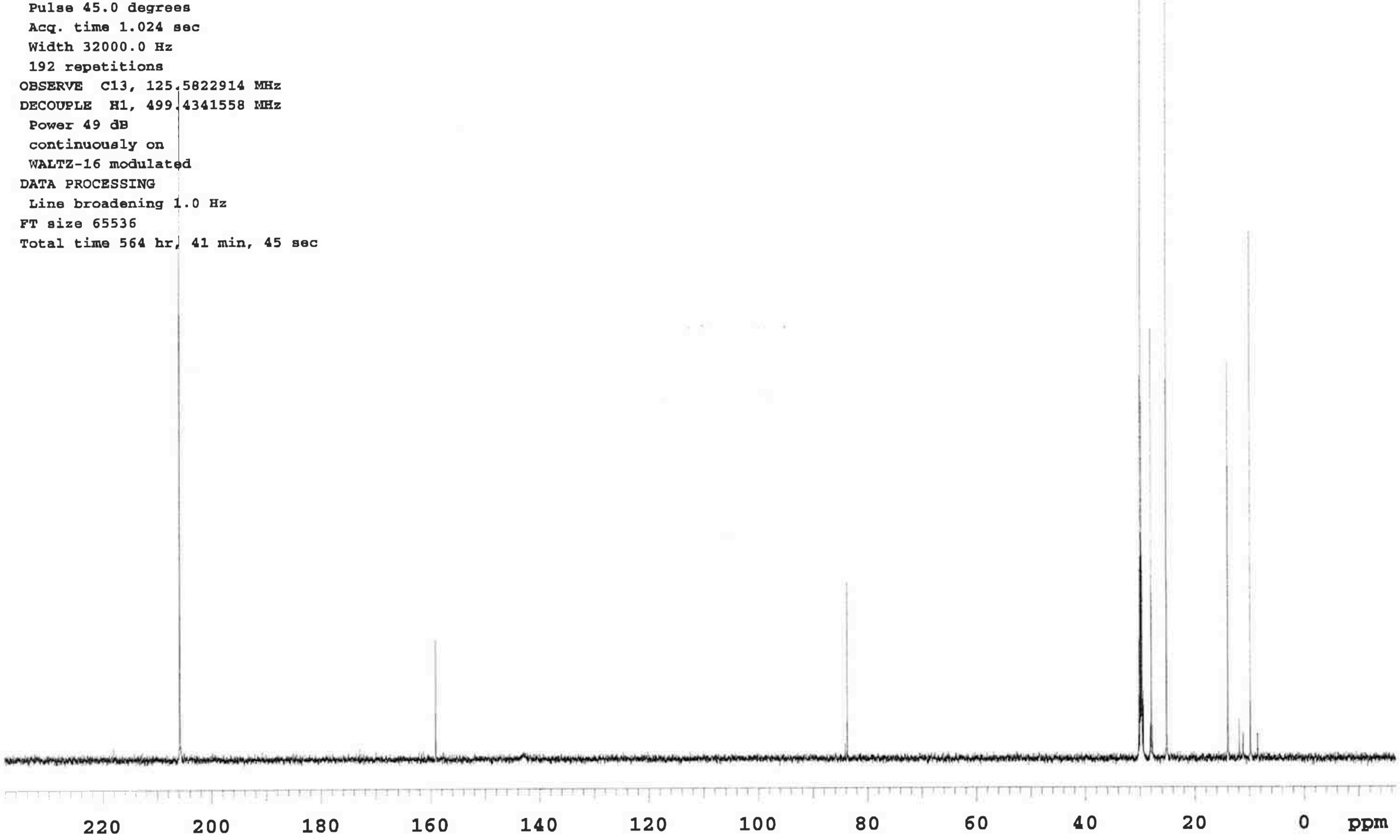

sf-Vinyl-iodo-MIDAboronate

Pulse Sequence: s2pul

Solvent: Acetone
Ambient temperature
INOVA-500 "u500"

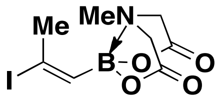

8

Pulse 61.6 degrees
Acq. time 4.665 sec
Width 7024.9 Hz
Single scan
OBSERVE H1, 499.6932568 MHz
DATA PROCESSING
Line broadening 0.3 Hz
FT size 65536
Total time 0 min, 4 sec

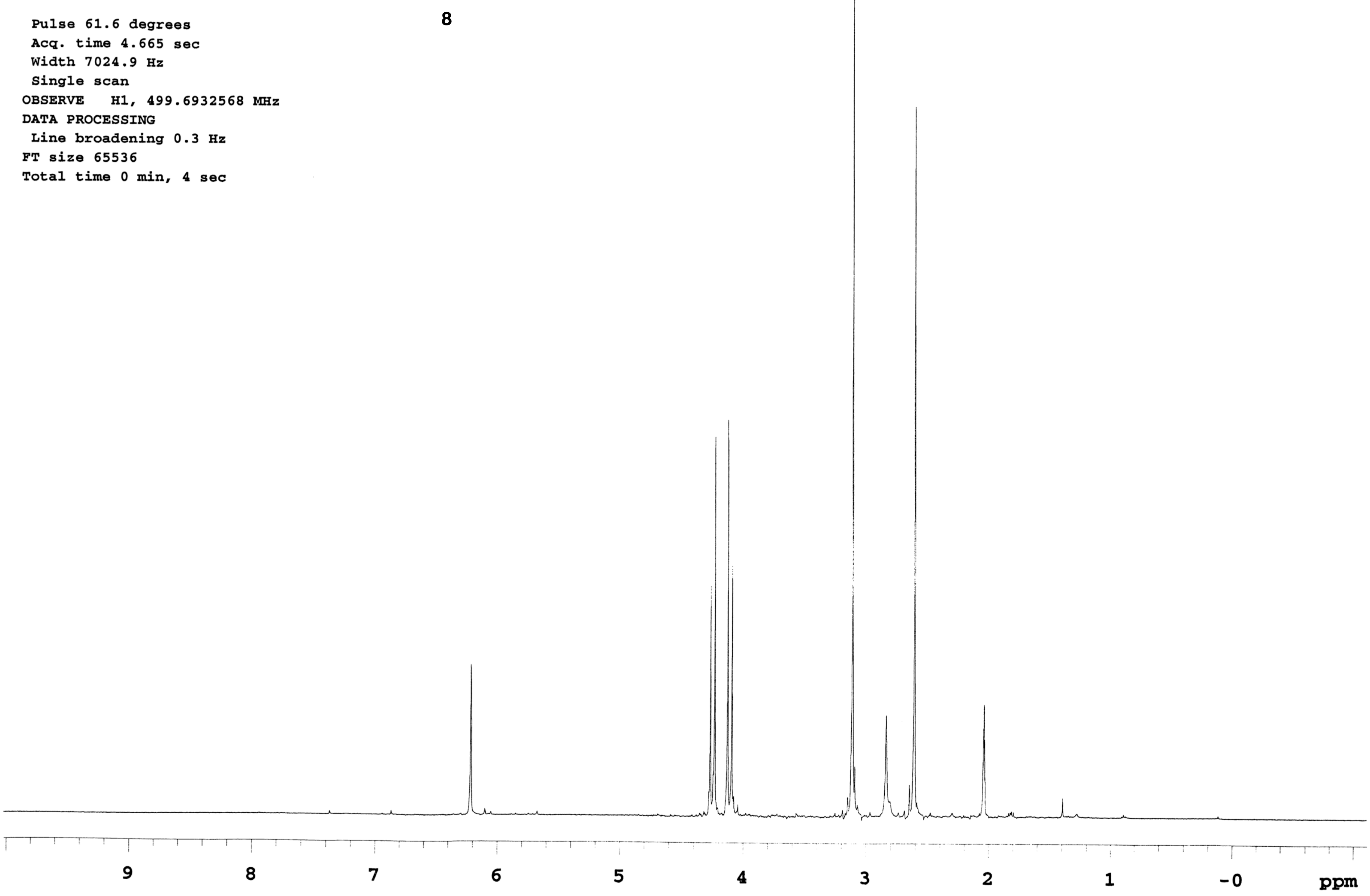

vinyl iodo MIDA boronate

Pulse Sequence: s2pul

Solvent: Acetone
Ambient temperature
User: 1-14-87
File: sf-iodo-vinyl-MIDAboronate
INOVA-500 "sunds1"

Relax. delay 1.000 sec
Pulse 65.9 degrees
Acq. time 1.086 sec
Width 30165.9 Hz
464 repetitions
OBSERVE C13, 125.6478629 MHz
DECOUPLE H1, 499.6949209 MHz
Power 44 dB
continuously on
WALTZ-16 modulated
DATA PROCESSING
Line broadening 1.0 Hz
FT size 65536
Total time 58 hr, 11 min, 58 sec

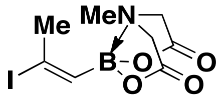

8

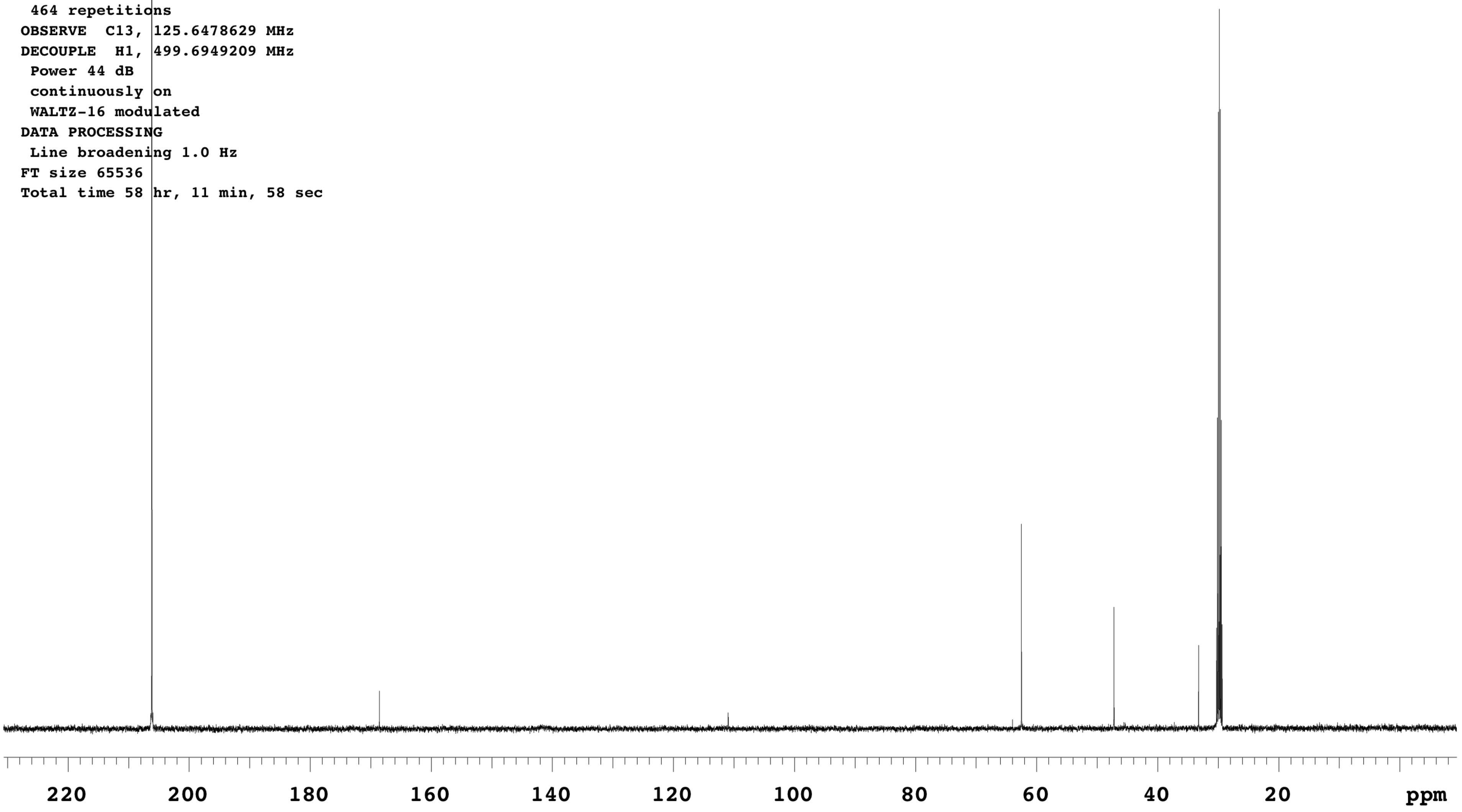

bifunctionaldiene-BB1

exp1 s2pul

SAMPLE		DEC. & VT	
date	Jul 30 2010	dfrq	500.073
solvent	Acetone	dn	H1
file	/export/home/~	dpwr	18
data/ui500nb/Burke~		dof	0
/fujiis/sf-viii-13~		dm	nnn
	3-f4-7.fid	dmm	c
ACQUISITION		dmf	200
sfrq	500.073	dseq	
tn	H1	dres	1.0
at	4.096	homo	n
np	65536	PROCESSING	
sw	8000.0	lb	0.30
fb	4000	wtfile	
bs	16	proc	ft
tpwr	55	fn	not used
pw	9.0	math	f
d1	0		
tof	0	werr	
nt	4	wexp	
ct	4	wbs	
alock	n	wnt	
gain	not used		
FLAGS			
il	n		
in	n		
dp	y		
hs	nn		
DISPLAY			
sp	-509.9		
wp	5493.2		
vs	29		
sc	0		
wc	250		
hzmm	21.97		
is	33.57		
rfl	2537.4		
rfp	1020.1		
th	2		
ins	1.000		
ai	ph		

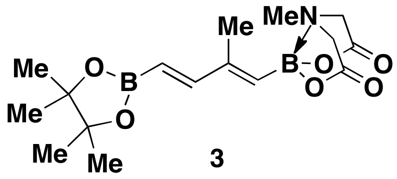

3

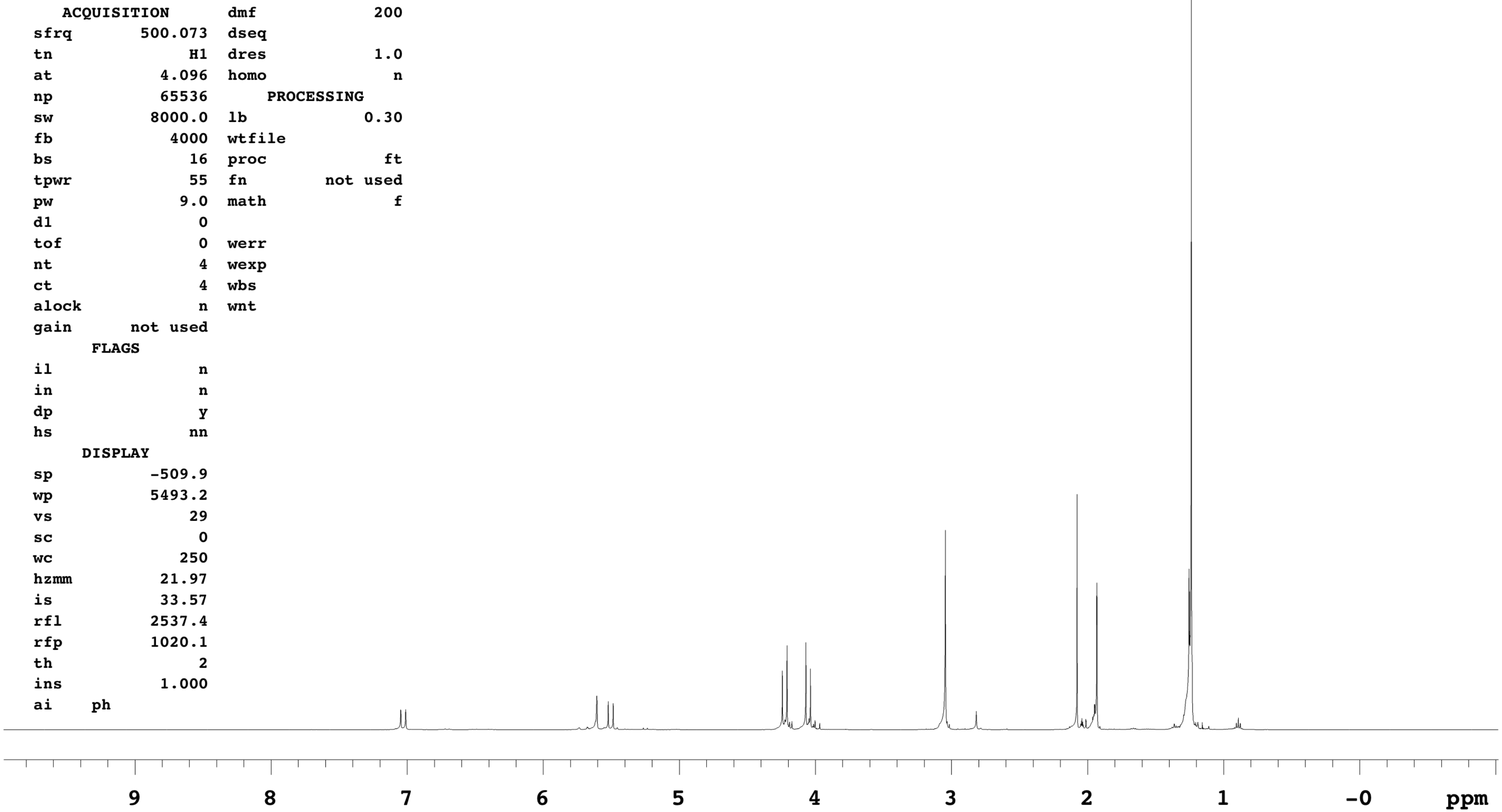

bifunctionaldiene-BB2

Pulse Sequence: s2pul

Solvent: Acetone
Ambient temperature
User: 1-14-87
File: bifunctionaldiene-BB2
INOVA-500 "sunds1"

Relax. delay 1.000 sec
Pulse 45.0 degrees
Acq. time 1.024 sec
Width 32000.0 Hz
240 repetitions
OBSERVE C13, 125.5822923 MHz
DECOUPLE H1, 499.4341558 MHz
Power 49 dB
continuously on
WALTZ-16 modulated
DATA PROCESSING
Line broadening 1.0 Hz
FT size 65536
Total time 564 hr, 41 min, 45 sec

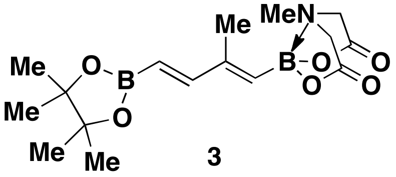

3

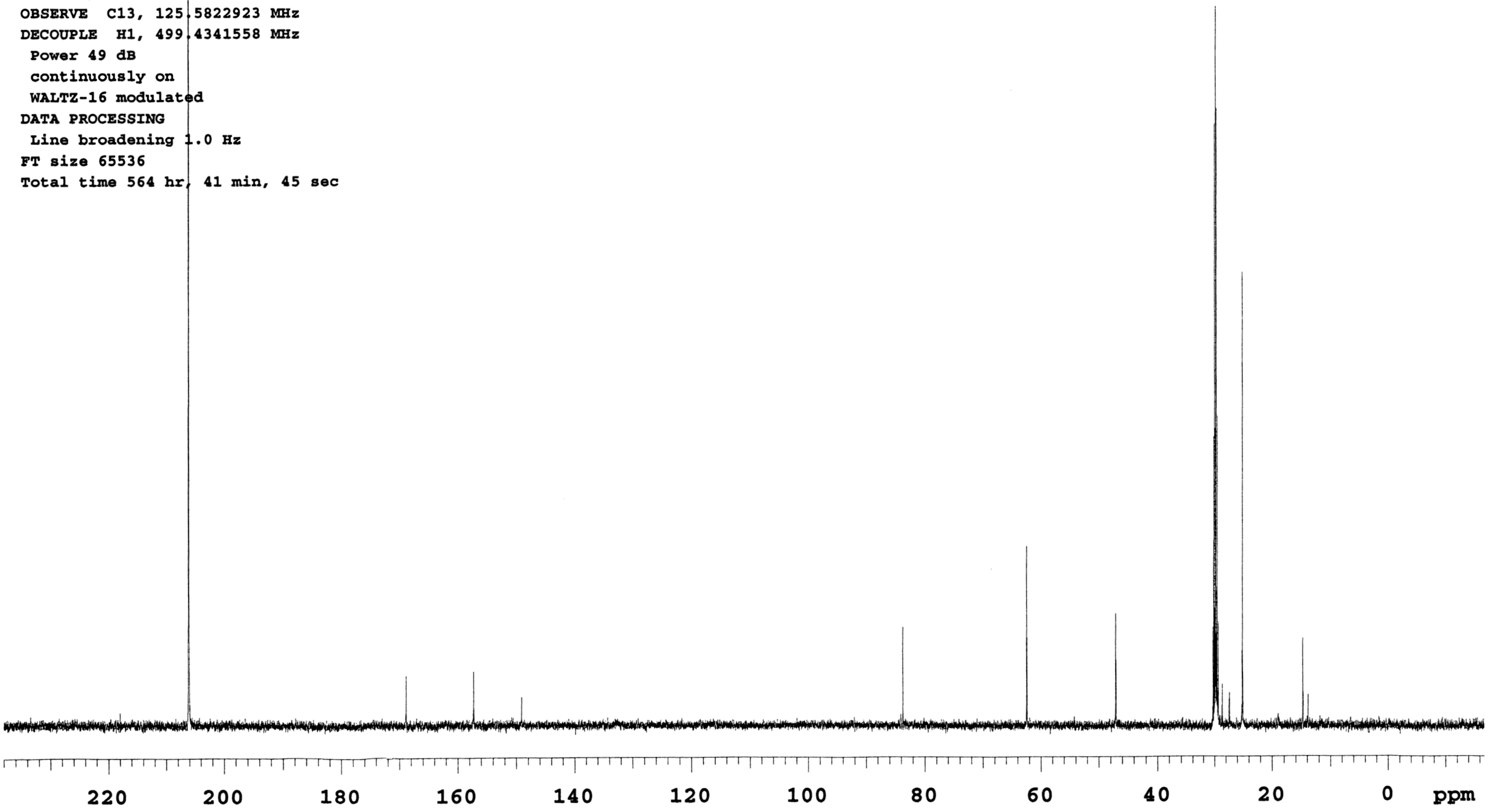

dienylMIDAboronate-1H

exp1 s2pul

SAMPLE		DEC. & VT	
date	Jul 30 2010	dfrq	500.073
solvent	Acetone	dn	H1
file /export/home/~		dpwr	18
data/ui500nb/Burke~		dof	0
/fujiis/sf-viii-10~		dm	nnn
3-np-4-12-pureest.~		dmm	c
	fid	dmf	200
ACQUISITION		dseq	
sfrq	500.073	dres	1.0
tn	H1	homo	n
at	4.096	PROCESSING	
np	65536	lb	0.30
sw	8000.0	wtfile	
fb	4000	proc	ft
bs	16	fn	not used
tpwr	55	math	f
pw	9.0		
d1	0	werr	
tof	0	wexp	
nt	4	wbs	
ct	4	wnt	
alock	n		
gain	not used		
FLAGS			
il	n		
in	n		
dp	y		
hs	nn		
DISPLAY			
sp	-509.9		
wp	5493.2		
vs	135		
sc	0		
wc	250		
hzmm	21.97		
is	33.57		
rfl	2537.4		
rfp	1020.1		
th	6		
ins	1.000		
ai	ph		

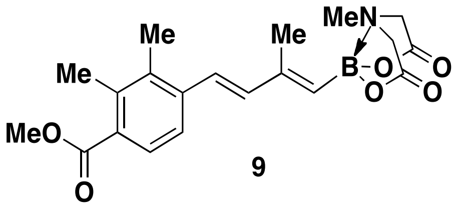

9

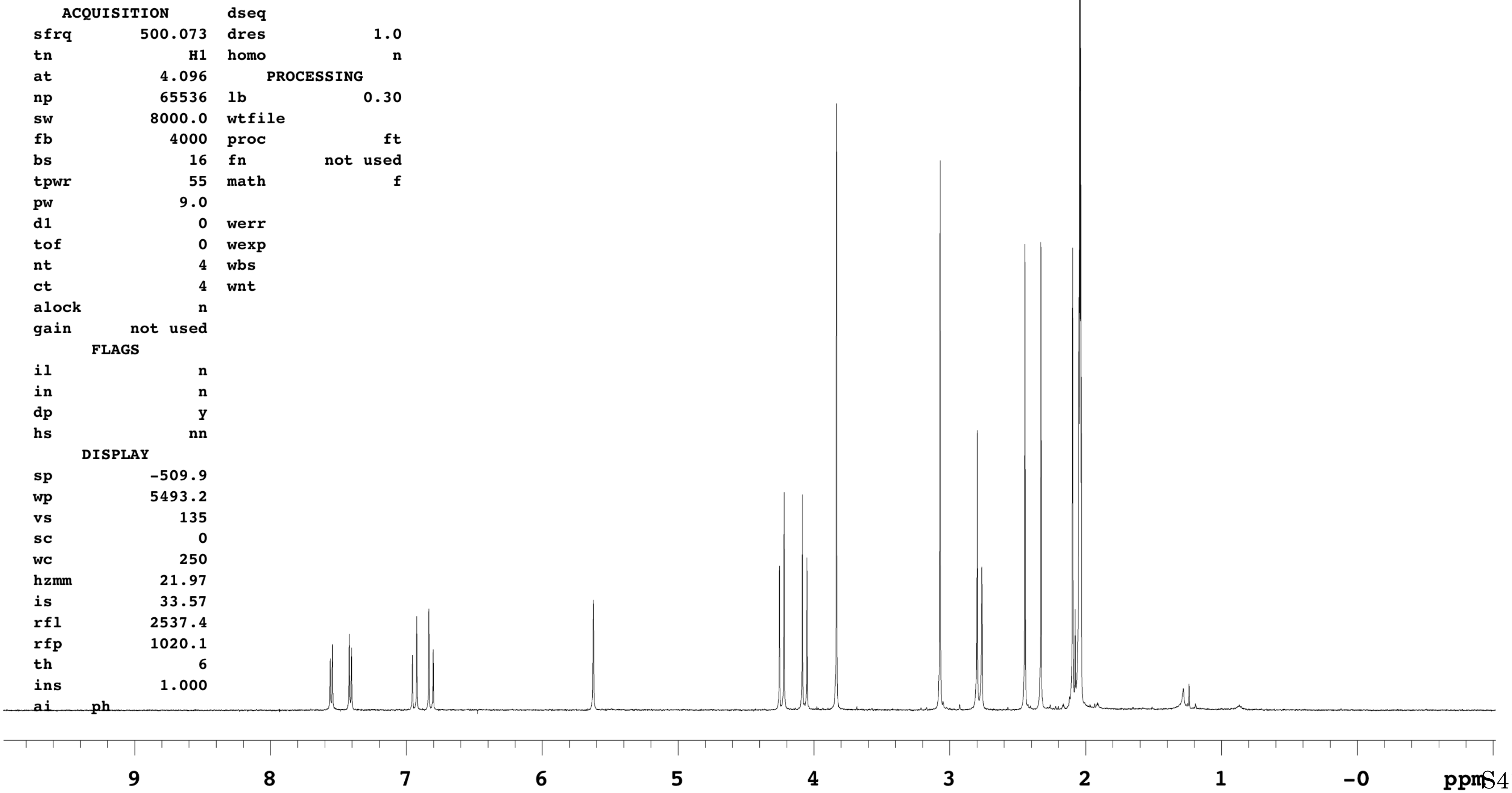

```
sf-IX-109-13C

exp1  s2pul

       SAMPLE                 DEC. & VT
date    Sep 17 2010    dfrq        499.695
solvent     Acetone    dn               H1
file            exp    dpwr             44
   ACQUISITION         dof          -827.6
sfrq        125.662    dm              yyy
tn              C13    dmm               w
at            1.086    dmf           19608
np            65536    dseq
sw          30165.9    dres           90.0
fb            17000    homo              n
bs               16           DEC2
ss                1    dfrq2             0
tpwr             54    dn2
pw              6.0    dpwr2             1
d1            1.000    dof2              0
tof          1884.7    dm2               n
nt             1000    dmm2              c
ct              320    dmf2          10000
alock             n    dseq2
gain       not used    dres2           1.0
      FLAGS            homo2             n
il                n        PROCESSING
in                n    lb             1.00
dp                y    wtfile
hs               nn    proc             ft
      DISPLAY          fn         not used
sp          -1184.5    math              f
wp          30165.0
vs              162    werr
sc                0    wexp
wc              250    wbs
hzmm          23.49    wnt
is           500.00
rfl          4929.8
rfp          3744.3
th               12
ins         100.000
nm     ph
```

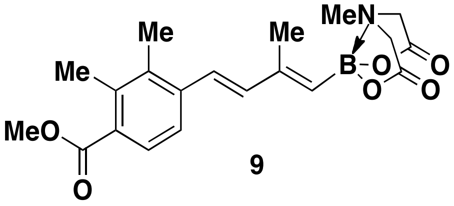

9

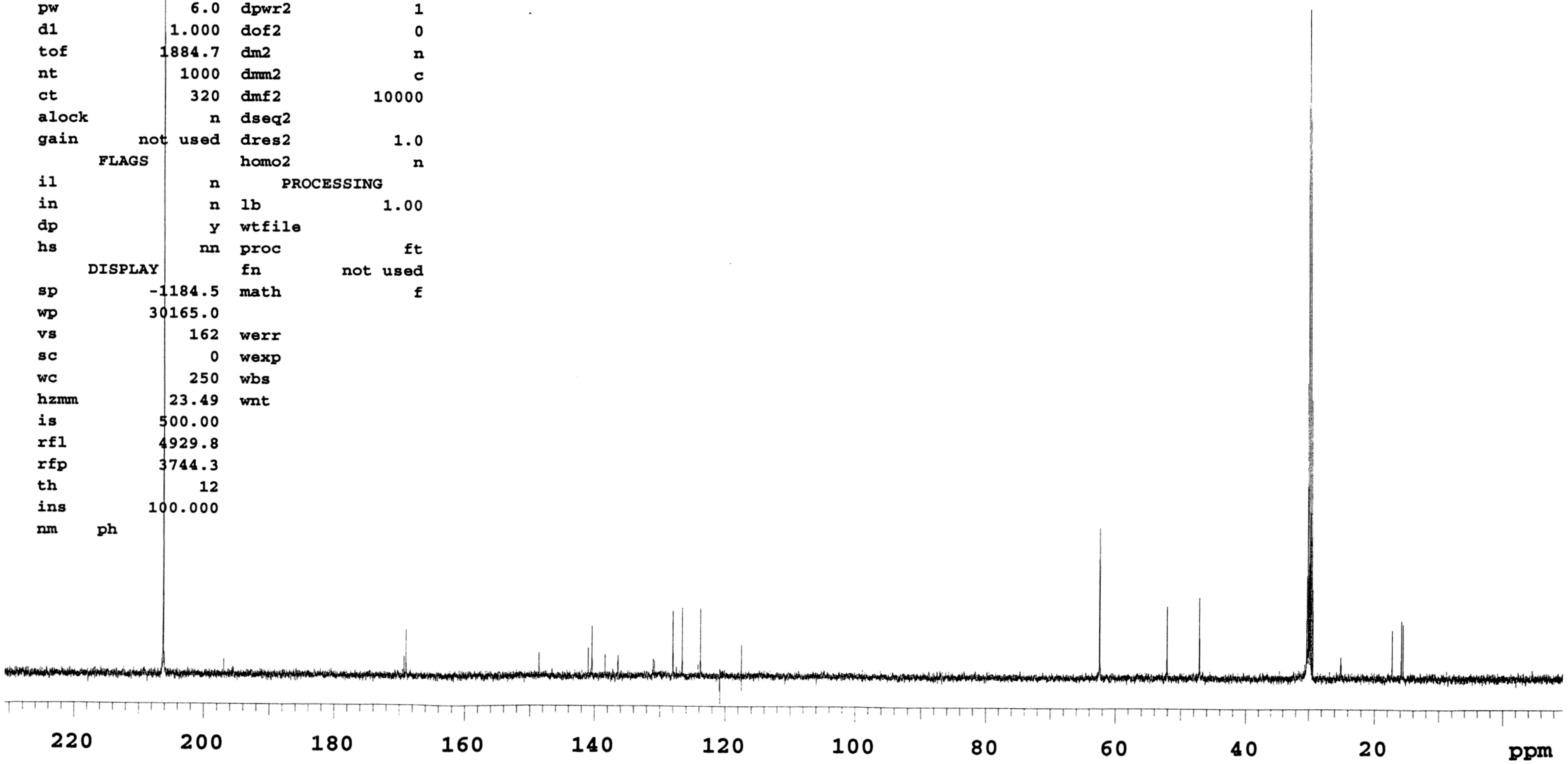

```
dienyliodide-1H

exp1  s2pul

      SAMPLE              DEC. & VT
date   Aug  3 2010  dfrq        500.073
solvent    Acetone  dn               H1
file /export/home/~ dpwr             18
data/ui500nb/Burke~ dof               0
/fujiis/sf-viii-13~ dm              nnn
       5-crude.fid  dmm               c
   ACQUISITION      dmf             200
sfrq       500.073  dseq
tn              H1  dres            1.0
at           4.096  homo              n
np           65536      PROCESSING
sw          8000.0  lb             0.30
fb            4000  wtfile
bs              16  proc             ft
tpwr            55  fn         not used
pw             9.0  math              f
d1               0
tof              0  werr
nt               4  wexp
ct               4  wbs
alock            n  wnt
gain      not used
      FLAGS
il               n
in               n
dp               y
hs              nn
     DISPLAY
sp          -509.9
wp          5504.9
vs             102
sc               0
wc             250
hzmm         22.02
is           33.57
rfl         2537.4
rfp         1020.1
th               5
ins          1.000
ai    ph
```

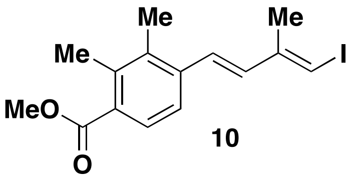

10

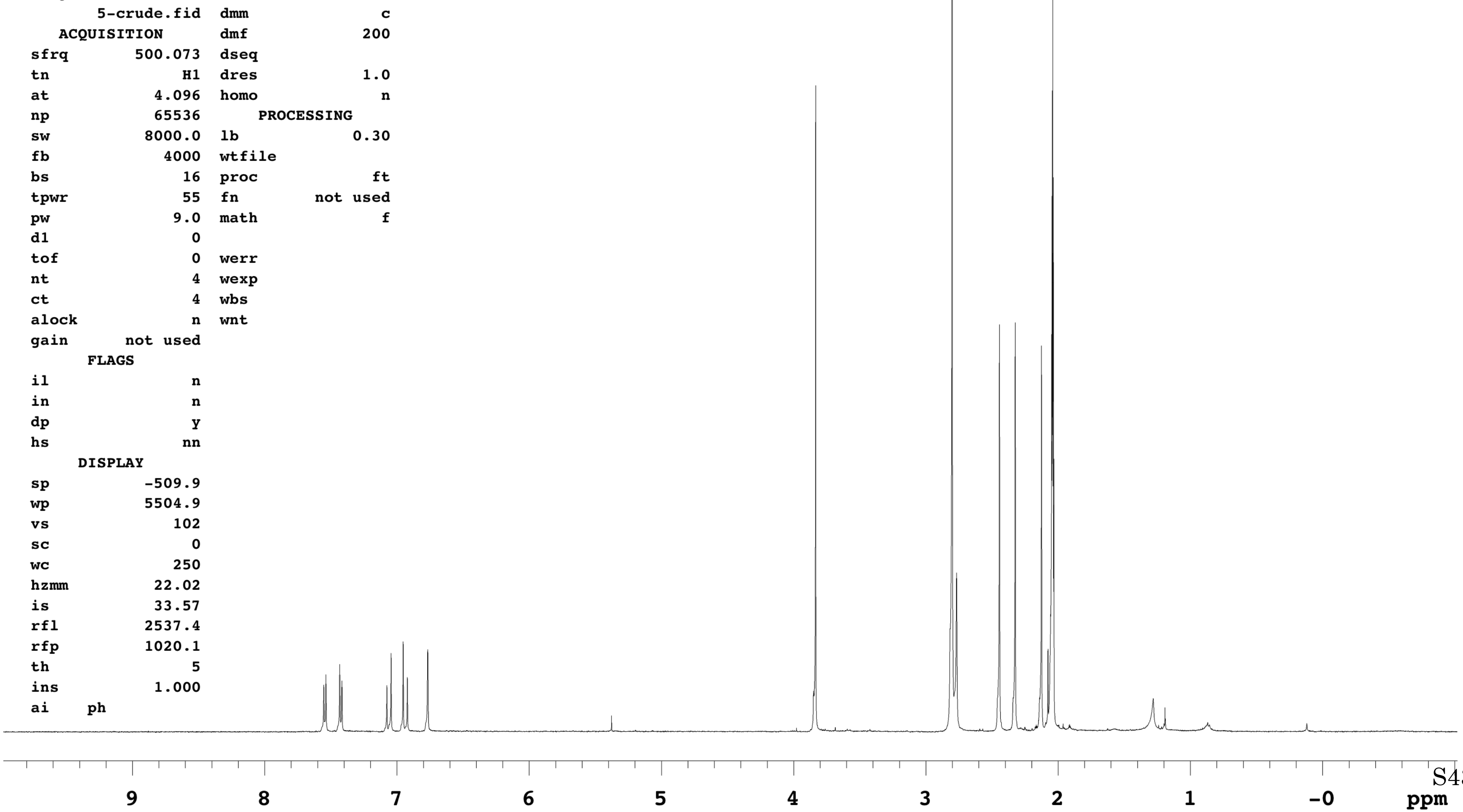

dienyl iodide

Pulse Sequence: s2pul

Solvent: Acetone
Ambient temperature
User: 1-14-87
File: sf-x-35-crude-13C
INOVA-500 "sunds1"

Relax. delay 1.000 sec
Pulse 65.9 degrees
Acq. time 1.086 sec
Width 30165.9 Hz
304 repetitions
OBSERVE C13, 125.6478693 MHz
DECOUPLE H1, 499.6949209 MHz
Power 44 dB
continuously on
WALTZ-16 modulated
DATA PROCESSING
Line broadening 1.0 Hz
FT size 65536
Total time 5 hr, 49 min, 13 sec

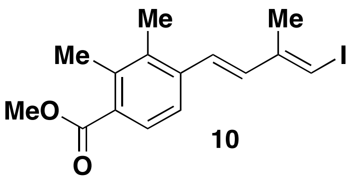

10

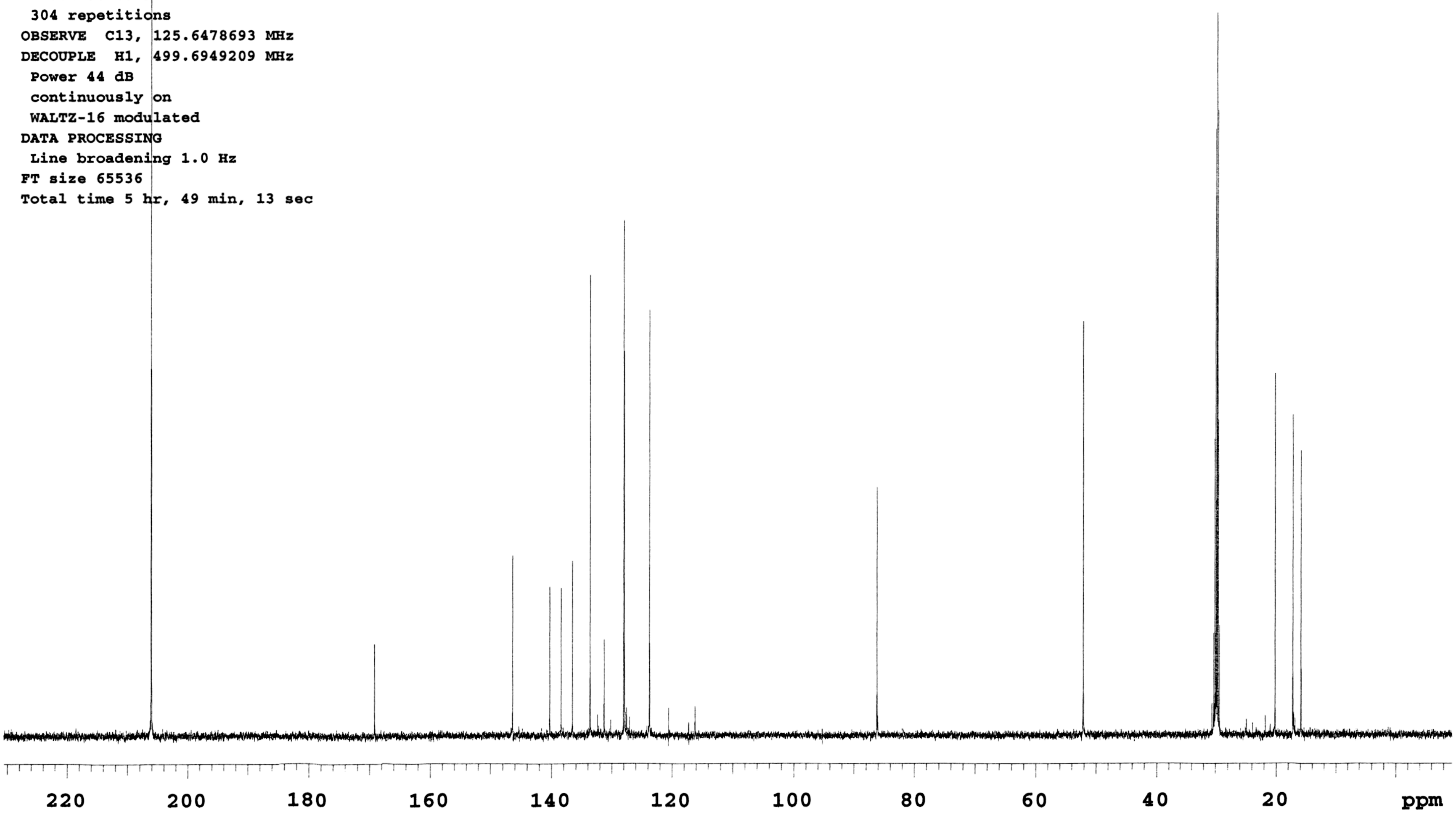

sf-xi-83-f6-24

exp1 s2pul

SAMPLE		DEC. & VT	
date	Feb 17 2011	dfrq	500.073
solvent	Acetone	dn	H1
file	exp	dpwr	18
ACQUISITION		dof	0
sfrq	500.073	dm	nnn
tn	H1	dmm	c
at	4.096	dmf	200
np	65536	dseq	
sw	8000.0	dres	1.0
fb	4000	homo	n
bs	16	DEC2	
tpwr	55	dfrq2	0
pw	9.0	dn2	
d1	0	dpwr2	1
tof	0	dof2	0
nt	4	dm2	n
ct	4	dmm2	c
alock	n	dmf2	200
gain	not used	dseq2	
FLAGS		dres2	1.0
il	n	homo2	n
in	n	PROCESSING	
dp	y	lb	0.30
hs	nn	wtfile	
DISPLAY		proc	ft
sp	-498.4	fn	not used
wp	5493.2	math	f
vs	69		
sc	0	werr	
wc	250	wexp	
hzmm	21.97	wbs	
is	33.57	wnt	
rfl	2537.6		
rfp	1020.1		
th	1		
ins	1.000		
ai cdc ph			

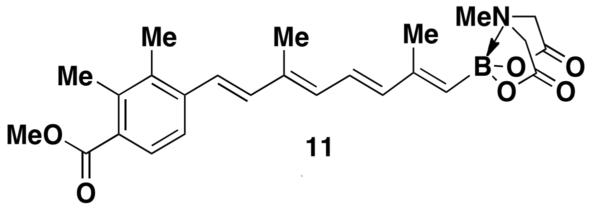

11

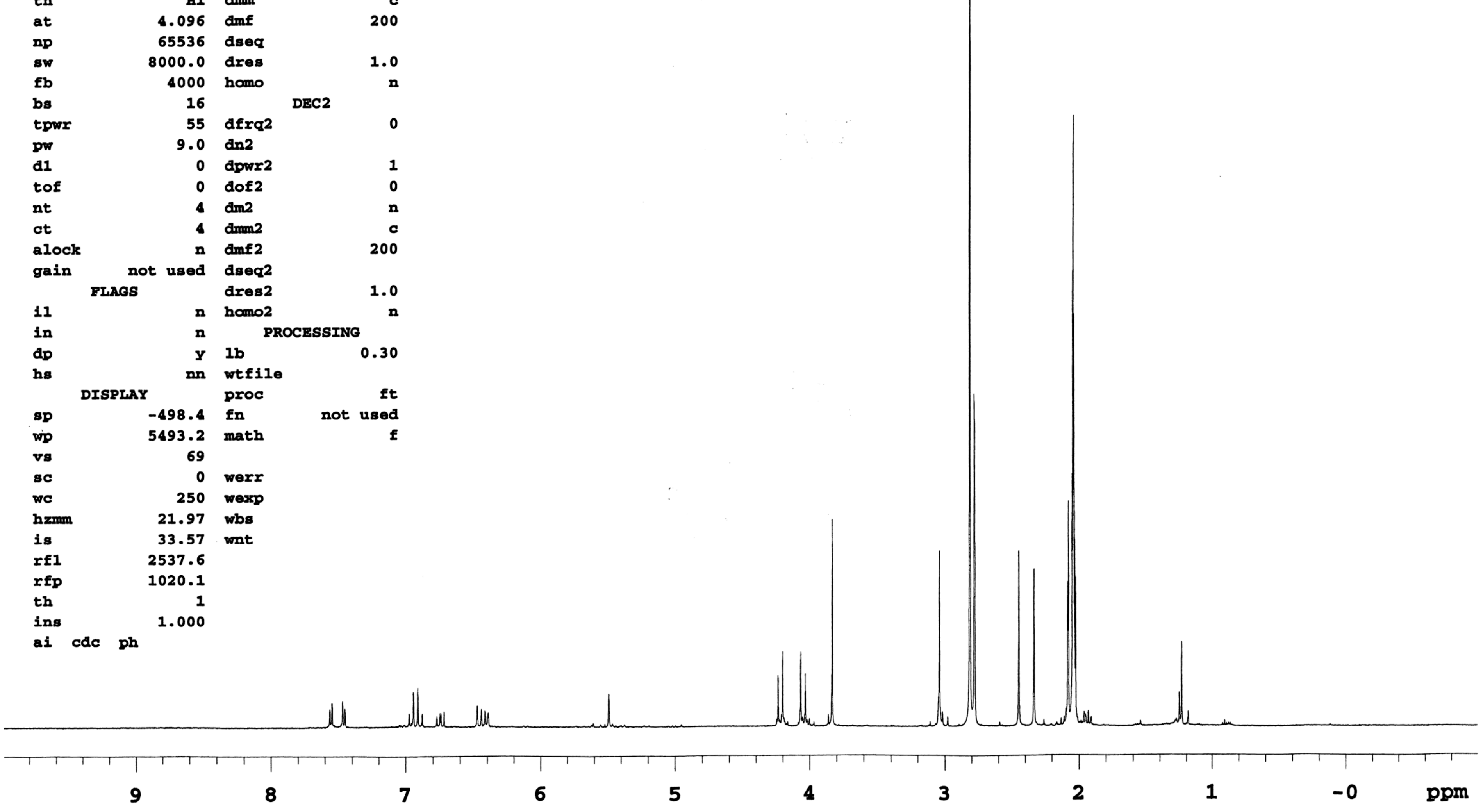

tetraenylMIDAboronate-13C

Pulse Sequence: s2pul

Solvent: Acetone
Ambient temperature
User: 1-14-87
File: sf-IX-57-13C
INOVA-500 "sunds1"

Relax. delay 1.000 sec
Pulse 45.0 degrees
Acq. time 1.024 sec
Width 32000.0 Hz
384 repetitions
OBSERVE C13, 125.5822904 MHz
DECOUPLE H1, 499.4341558 MHz
Power 49 dB
continuously on
WALTZ-16 modulated
DATA PROCESSING
Line broadening 1.0 Hz
FT size 65536
Total time 5646 hr, 57 min, 26 sec

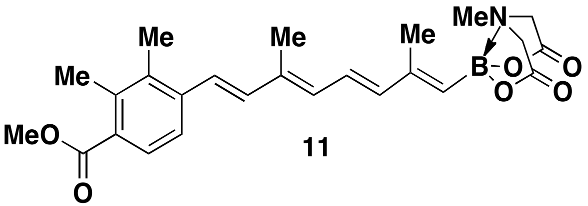

11

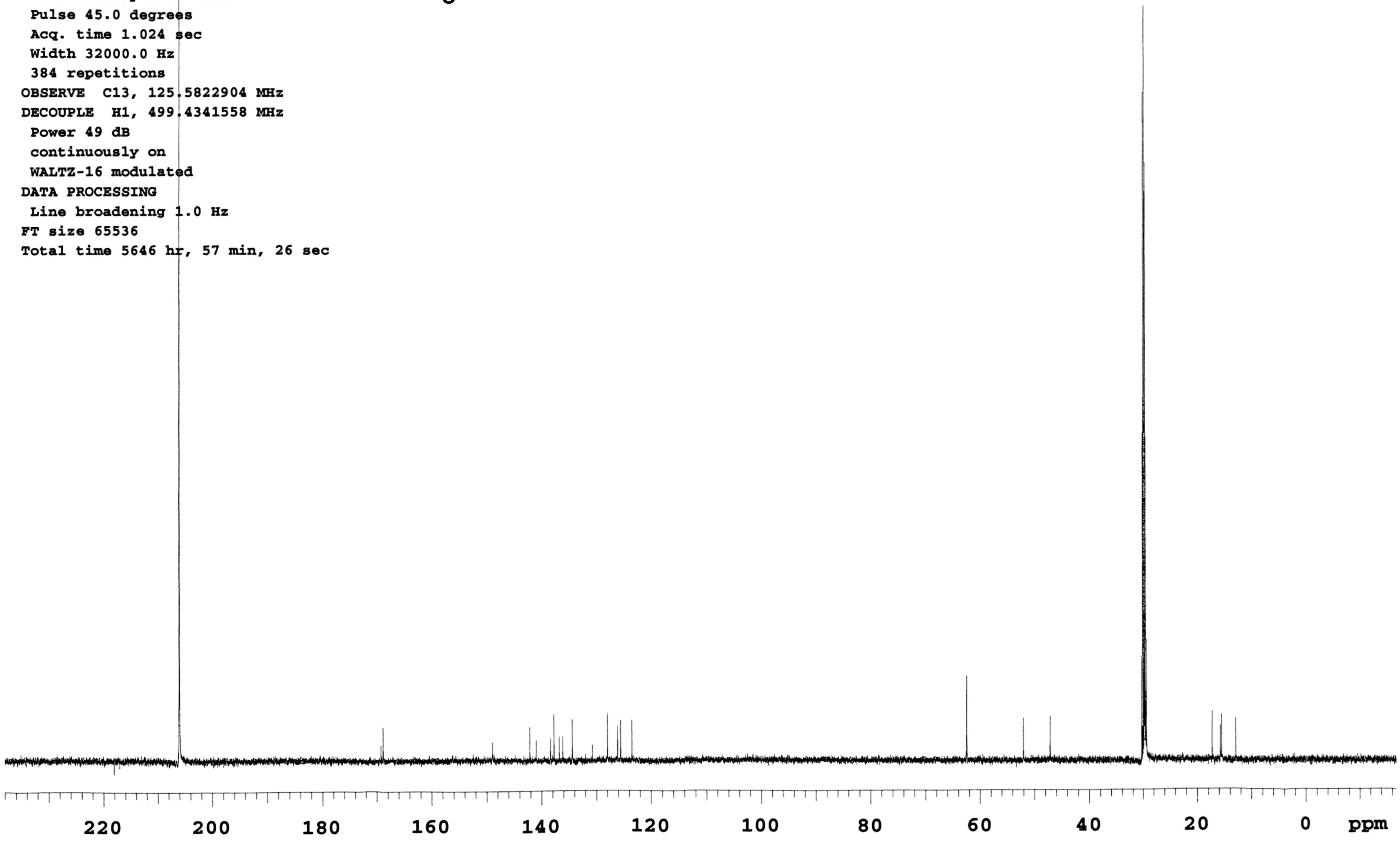

sf-xi-113-cd2cl2

Pulse Sequence: s2pul

Solvent: CD2Cl2
Ambient temperature
INOVA-500 "ui500nb"

Relax. delay 20.000 sec
Pulse 90.0 degrees
Acq. time 4.096 sec
Width 8000.0 Hz
4 repetitions
OBSERVE H1, 500.0687428 MHz
DATA PROCESSING
Line broadening 0.3 Hz
FT size 65536
Total time 1 min, 36 sec

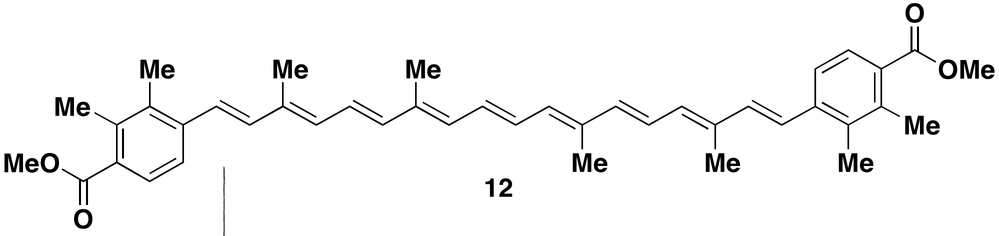

12

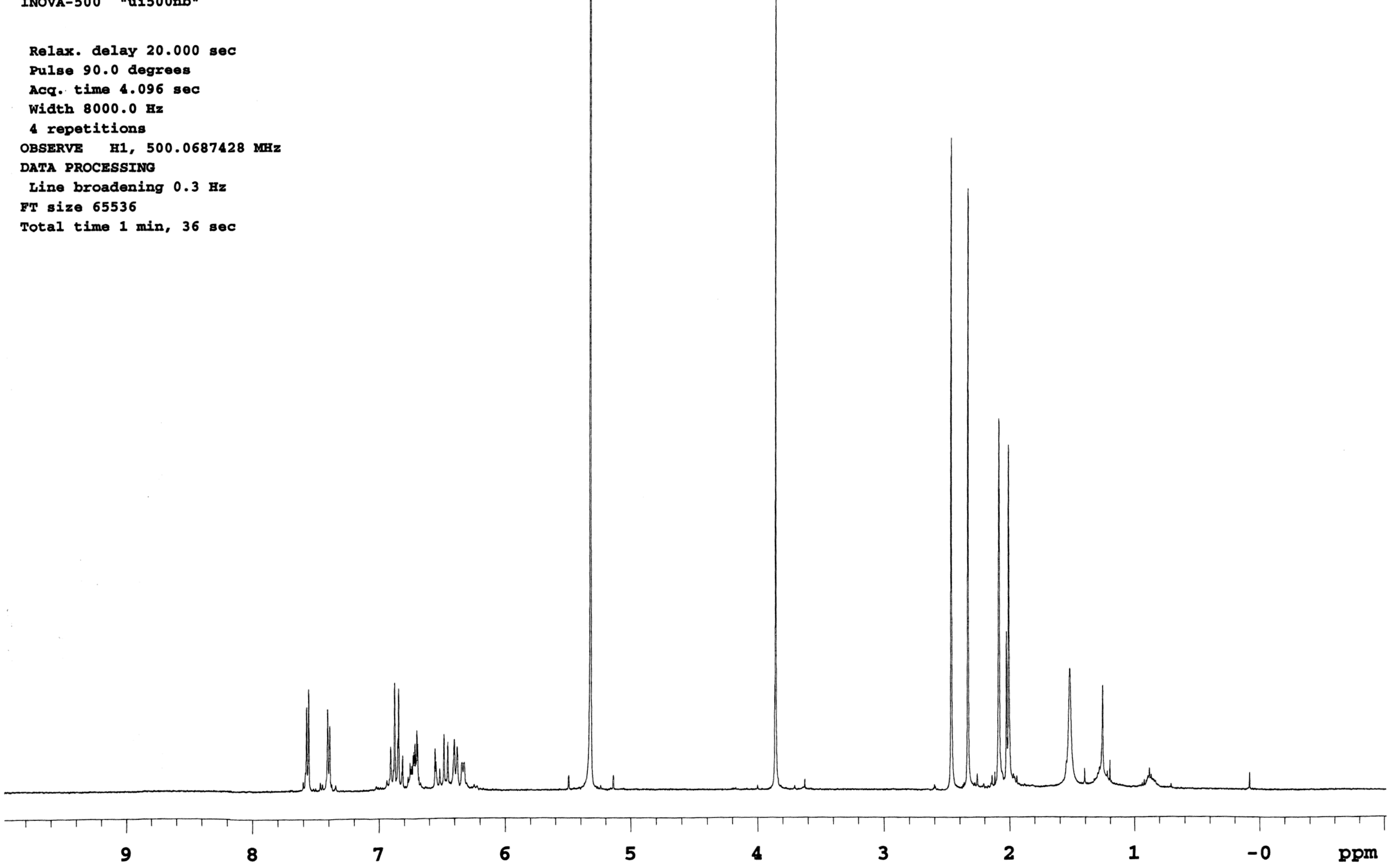

methylester synechoxanthin

Pulse Sequence: s2pul

Solvent: CD2Cl2
Ambient temperature
User: 1-14-87
File: sf-IX-101-f14-20-13C
INOVA-500 "sunds1"

Relax. delay 1.000 sec
Pulse 65.9 degrees
Acq. time 1.086 sec
Width 30165.9 Hz
2054 repetitions
OBSERVE C13, 125.6475085 MHz
DECOUPLE H1, 499.6932869 MHz
Power 44 dB
continuously on
WALTZ-16 modulated
DATA PROCESSING
Line broadening 1.0 Hz
FT size 65536
Total time 581990 hr, 19 min, 44 sec

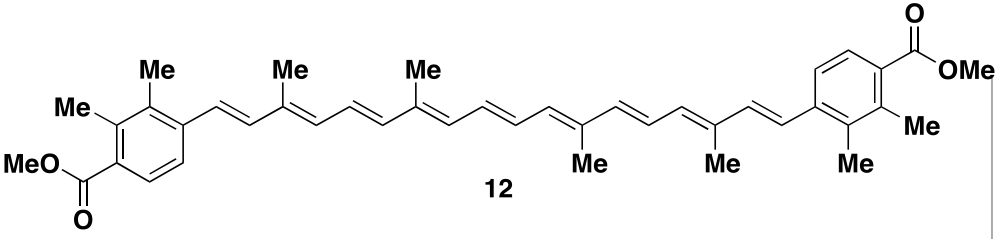

12

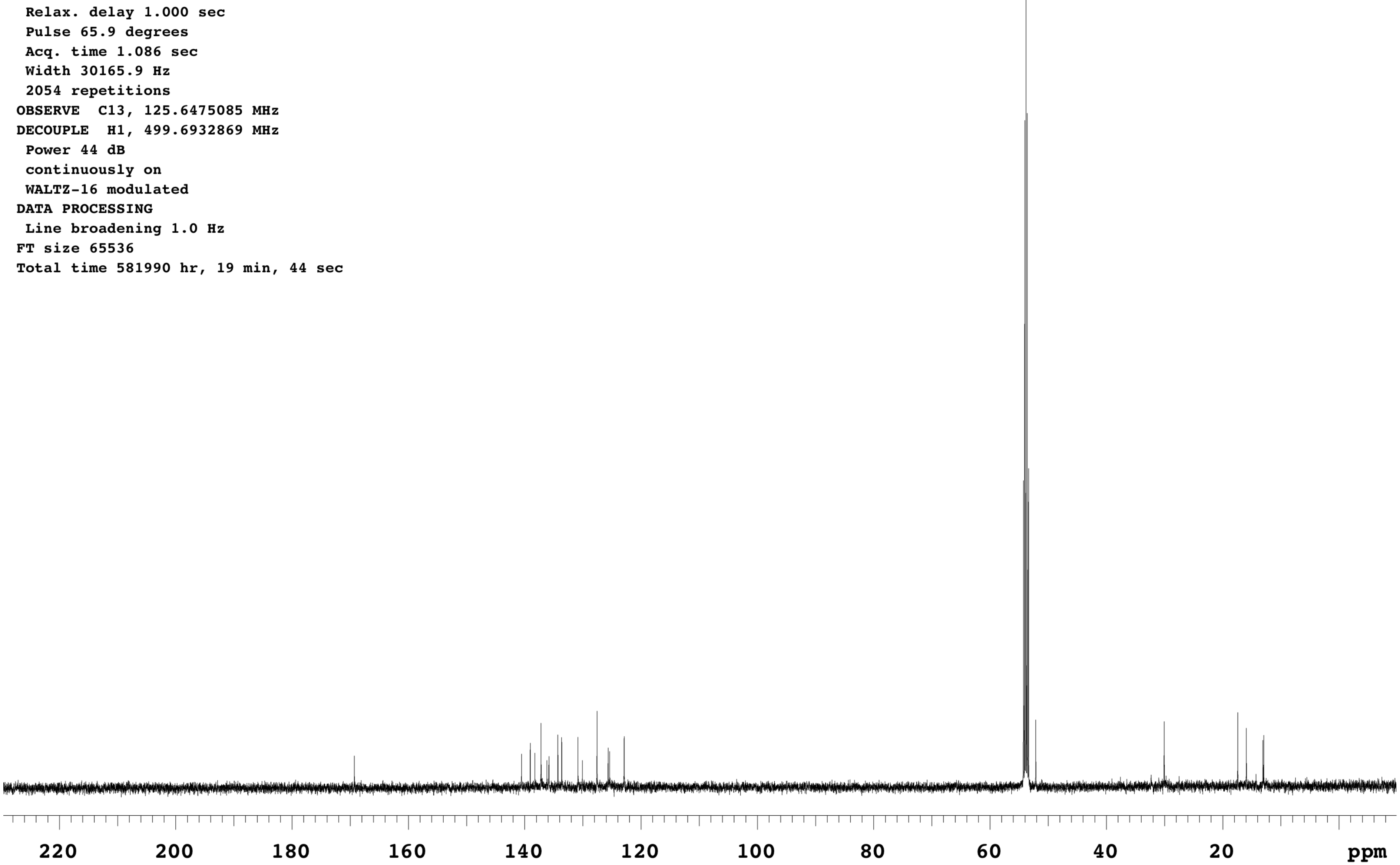

sf-xi-117-synechoxanthin

Pulse Sequence: s2pul

Solvent: CD3OD
Ambient temperature
INOVA-500 "ui500nb"

Pulse 90.0 degrees
Acq. time 4.096 sec
Width 8000.0 Hz
128 repetitions
OBSERVE H1, 500.0697967 MHz
DATA PROCESSING
Line broadening 0.3 Hz
FT size 65536
Total time 1 hr, 8 min, 24 sec

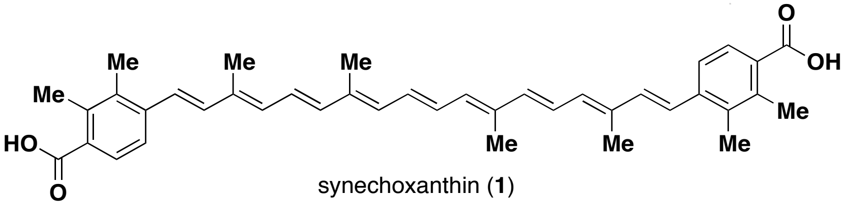

synechoxanthin (**1**)

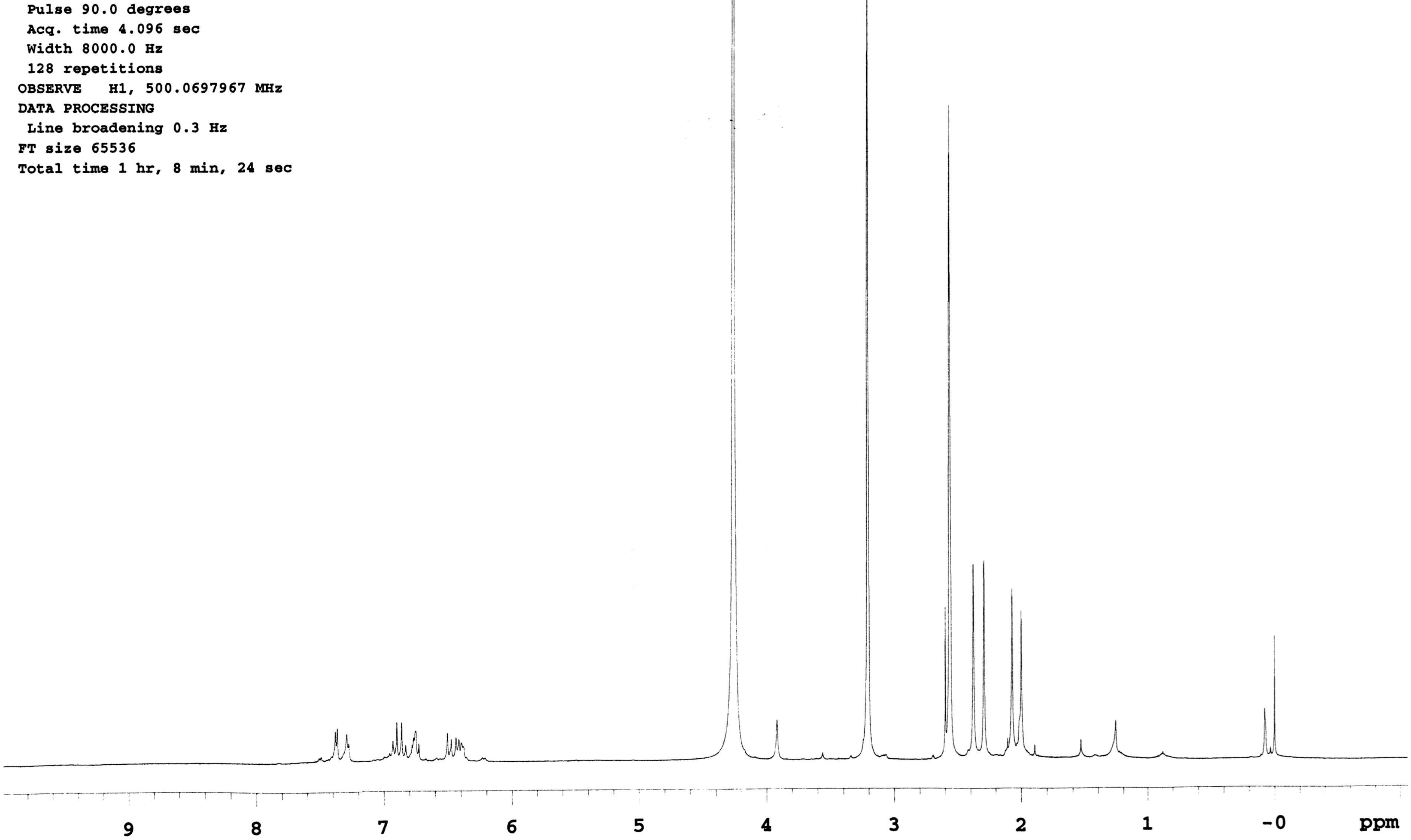

sf-xi-127-1H-U500

Pulse Sequence: s2pul

Solvent: CD2Cl2
Ambient temperature
INOVA-500 "u500"

Pulse 61.6 degrees
Acq. time 4.665 sec
Width 7024.9 Hz
56 repetitions
OBSERVE H1, 499.6916241 MHz
DATA PROCESSING
Line broadening 0.3 Hz
FT size 65536
Total time 7 min, 47 sec

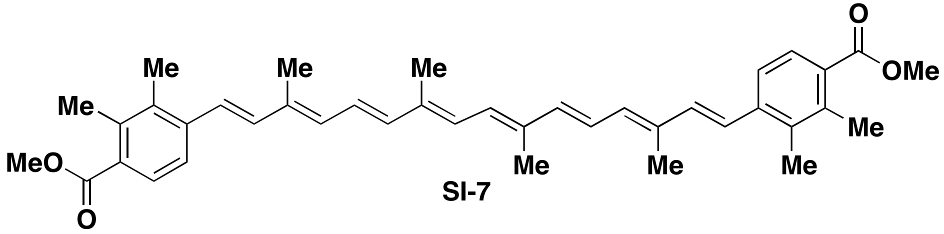

SI-7

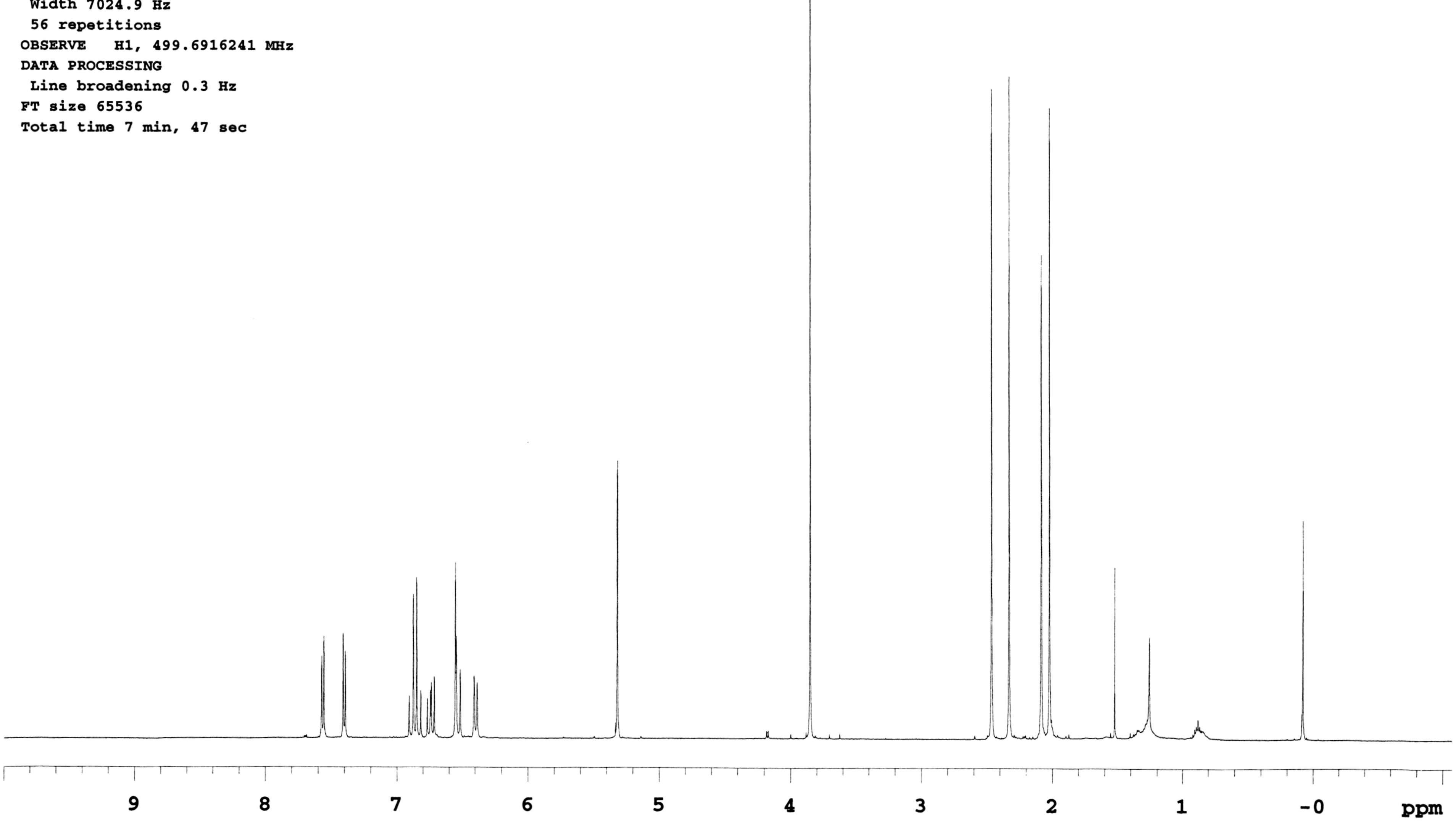

STANDARD CARBON PARAMETERS

Pulse Sequence: s2pul

Solvent: CD2Cl2
Ambient temperature
User: 1-14-87
INOVA-500 "vxr500"

Relax. delay 1.000 sec
Pulse 45.0 degrees
Acq. time 1.024 sec
Width 32000.0 Hz
22432 repetitions
OBSERVE C13, 125.5819370 MHz
DECOUPLE H1, 499.4325227 MHz
Power 48 dB
continuously on
WALTZ-16 modulated
DATA PROCESSING
Line broadening 1.0 Hz
FT size 65536
Total time 564695 hr, 36 min, 32 sec

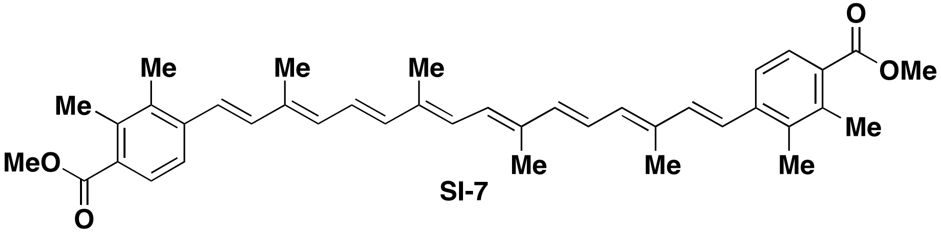

SI-7

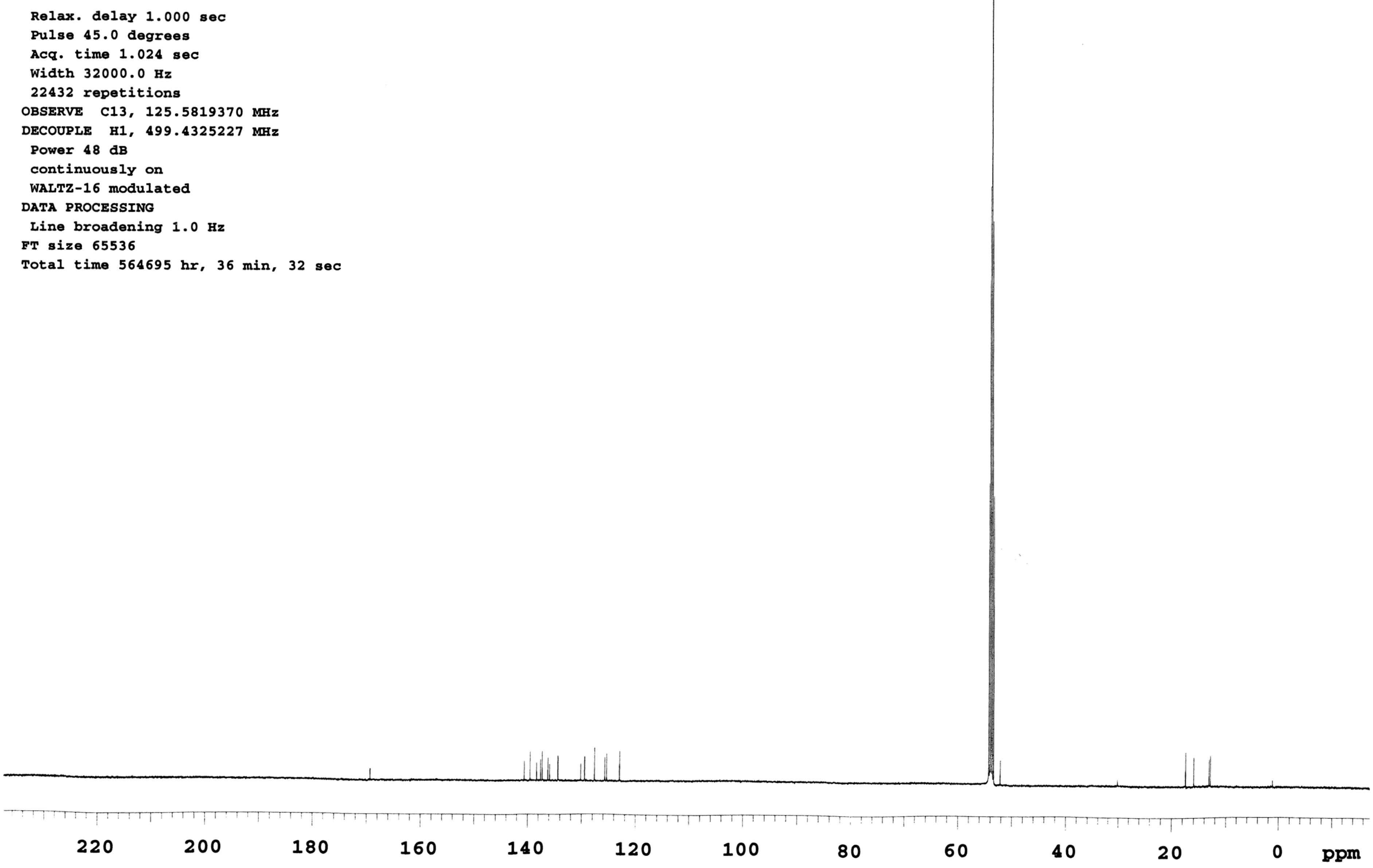

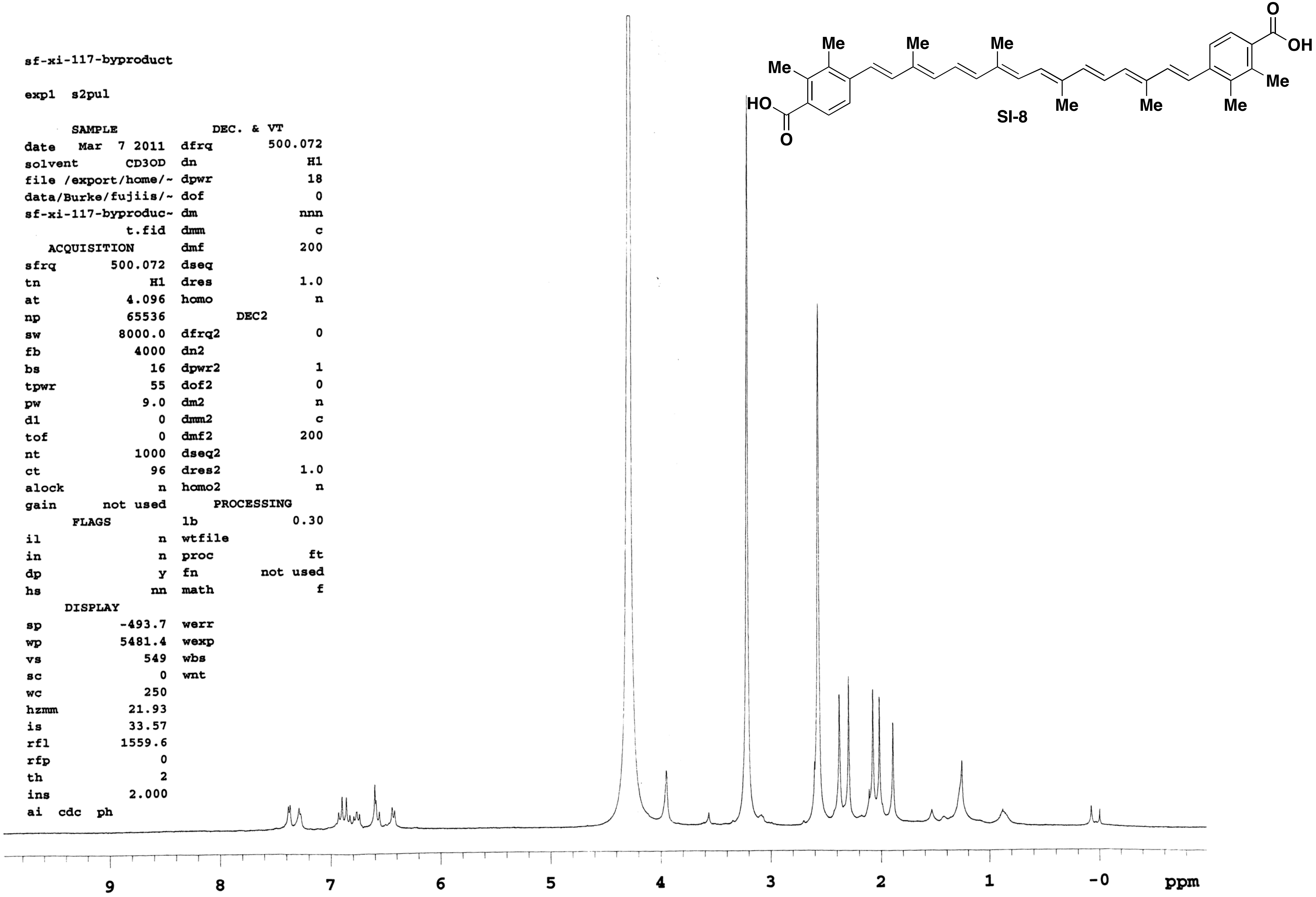
sf-xi-117-byproduct
exp1 s2pul
SAMPLE
date Mar 7 2011
solvent CD3OD
file /export/home/~
data/Burke/fujiis/~
sf-xi-117-byproduc~
t.fid
ACQUISITION
sfrq 500.072
tn H1
at 4.096
np 65536
sw 8000.0
fb 4000
bs 16
tpwr 55
pw 9.0
d1 0
tof 0
nt 1000
ct 96
alock n
gain not used
FLAGS
il n
in n
dp y
hs nn
DISPLAY
sp -493.7
wp 5481.4
vs 549
sc 0
wc 250
hzmm 21.93
is 33.57
rfl 1559.6
rfp 0
th 2
ins 2.000
ai cdc ph
DEC. & VT
dfrq 500.072
dn H1
dpwr 18
dof 0
dm nnn
dmm c
dmf 200
dseq
dres 1.0
homo n
DEC2
dfrq2 0
dn2
dpwr2 1
dof2 0
dm2 n
dmm2 c
dmf2 200
dseq2
dres2 1.0
homo2 n
PROCESSING
lb 0.30
wtfile
proc ft
fn not used
math f
werr
wexp
wbs
wnt
9
8
7
6
5
4
3
2
1
-0
ppm
Me
Me
Me
Me
Me
Me
Me
Me
O
OH
HO
O
SI-8
